# Comparative morphology refines the conventional model of spider reproduction

**DOI:** 10.1371/journal.pone.0218486

**Published:** 2019-07-05

**Authors:** Yongjia Zhan, He Jiang, Qingqing Wu, Huitao Zhang, Zishang Bai, Matjaž Kuntner, Lihong Tu

**Affiliations:** 1 College of Life Sciences, Capital Normal University, Beijing, P. R. China; 2 Lang Yue Campus of Beijing 12th High School, Beijing, P. R. China; 3 Beijing Advanced Innovation Center for Imaging Technology, Capital Normal University, Beijing, P. R. China; 4 College of Life Sciences, China Agricultural University, Beijing, P. R. China; 5 Evolutionary Zoology Laboratory, Department of Organisms and Ecosystems Research, National Institute of Biology, Ljubljana, Slovenia; 6 Evolutionary Zoology Laboratory, Biological Institute ZRC SAZU, Ljubljana, Slovenia; Laboratoire de Biologie du Développement de Villefranche-sur-Mer, FRANCE

## Abstract

Our understanding of spider reproductive biology is hampered by the vast anatomical diversity and difficulties associated with its study. Although authors agree on the two general types of female spider genitalia, haplogyne (plesiomorphic) and entelegyne (apomorphic), our understanding of variation within each group mostly concerns the external genital part, while the internal connections with the reproductive duct are largely unknown. Conventionally and simplistically, the spermathecae of haplogynes have simple two-way ducts, and those of entelegynes have separate copulatory and fertilization ducts for sperm to be transferred in and out of spermathecae, respectively. Sperm is discharged from the spermathecae directly into the *uterus externus* (a distal extension of the oviduct), which, commonly thought as homologous in both groups, is the purported location of internal fertilization in spiders. However, the structural evolution from haplo- to entelegyny remains unresolved, and thus the precise fertilization site in entelegynes is ambiguous. We aim to clarify this anatomical problem through a widely comparative morphological study of internal female genital system in entelegynes. Our survey of 147 epigyna (121 examined species in 97 genera, 34 families) surprisingly finds no direct connection between the fertilization ducts and the *uterus externus*, which, based on the homology with basal-most spider lineages, is a dead-end caecum in entelegynes. Instead, fertilization ducts usually connect with a *secondary uterus externus*, a novel feature taking over the functional role of the plesiomorphic *uterus externus*. We hypothesize that the transition from haplo- to entelegyny entailed not only the emergence of the two separate duct systems (copulatory, fertilization), but also involved substantial morphological changes in the distal part of the oviduct. Thus, the common oviduct may have shifted its distal connection from the *uterus externus* to the *secondary uterus externus*, perhaps facilitating discharge of larger eggs. Our findings suggest that the conventional model of entelegyne reproduction needs redefinition.

## Introduction

Animal genital systems responsible for internal fertilization are mostly well understood, their functional anatomy requiring coordinated structures (external and internal parts) in females. Spiders are believed to undertake internal fertilization, and their overall functional anatomic model seems to be understood [1]. However, the reality is that detailed direct functional anatomical investigations of spider genitalia are rare [2–5]. Our understanding of spider reproductive biology is largely hampered by the immense species richness of spiders, as well as the logical consequence of this richness: a huge diversity of their anatomies. Furthermore, anatomical legacy research has been limited by the available technology (for methodological overview, see S1 File). Most authors agree on the two general types of female spider genitalia. The plesiomorphic condition, found in basal-most spider clades Mesothelae, Mygalomorphae, and all the remaining clades of Araneomorphae excluding Entelegynae, is termed *haplogyne*. The apomorphic condition, found in the derived spider group Entelegynae that contains the majority of spider species, is termed *entelegyne* [1]. However, previous studies largely focus on the external genital anatomical variation within each group (for overview of female reproductive system in spiders, see S2 File). Consequently, the functional anatomies, as well as the hypothesized evolutionary transition from haplogyny to entelegyny remain vague.

The conventional model of spider reproduction predicts that sperm, initially deposited in the spermathecae (sperm storage organ), will be discharged into the *uterus externus*, a distal extension of the oviduct [1], when eggs are laid out to be fertilized (for literature overview on the relationships among fertilization ducts, *uterus externus* and oviduct, see S3 File). In haplogyne spiders the female genital opening, located within a ventral abdominal integumental fold termed the *epigastric furrow*, is used for both copulation and oviposition [1,6]. The spermathecae in haplogynes have single two-way ducts that bring sperm into and out of the spermathecae, and directly open to the *uterus externus* where internal fertilization takes place. On the other hand, the spermathecae in entelegyne spiders have two openings with copulatory and fertilization ducts for sperm entering and leaving the spermathecae, respectively; and the latter is also supposed to connect with the *uterus externus*. Forster [7] hypothesized that the entelegyne state evolved from the haplogyne one by the single spermathecal opening migrating from the epigastric furrow to the surface, and developing another duct to connect spermathecae with *uterus externus*. How this shift may have happened, and how the new connections established, remain unresolved [8].

If this textbook model of internal fertilization were true in entelegyne spiders, then one would expect to find a physical connection between the fertilization ducts and the *uterus externus* across species. Although a membranous column is often labeled as *uterus externus* in the scanning electronic microscopic (SEM) images [9–11], such connection has not been unequivocally demonstrated. The purported evidence largely comes from histological serial sections (HSS) [2–4], an approach riddled with difficulties (S1 File). The state of the art is that of an unclear anatomical correspondence of homologies, and as its consequence, a poor understanding of their function. Consequently, the anatomical site of internal fertilization in entelegyne spiders, if true, remains obscure [12,13].

We revisit this anatomical enigma within a widely comparative morphological study of entelegyne spider genitalia, specifically testing the existence of the hypothesized connection between the fertilization tract and the oviduct. Through combining computerized 3D-reconstruction based on semithin HSS images of the spider abdomen with light microscopic (LM) and SEM methods based on improved dissections, we examined the internal reproductive anatomy of 147 specimens of 121 spider species representing 97 genera from 34 families. Broadly speaking, the presence of a clear anatomical connection enabling sperm to enter the *uterus externus* would lend support for the conventional model of spider reproduction, whereas its absence would call for refinement of the classical model of spider internal fertilization.

## Results

**Fig 1:**
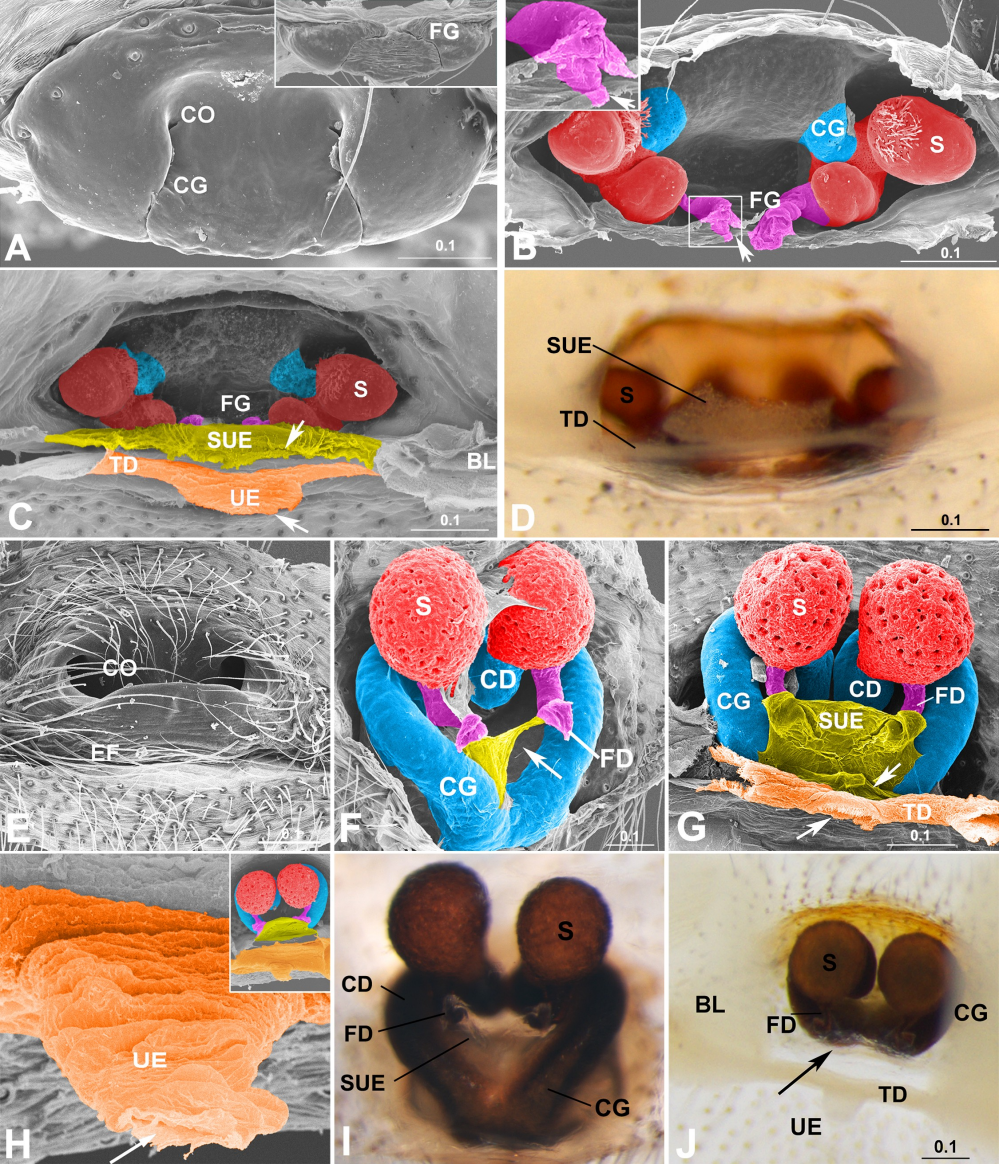
Epigynal tracts and epigastric furrow. (A–D) *Diphya wulingensis* (Tetragnathidae). (E–J) *Parasteatoda tepidariorum* (Theridiidae). (A) Ventral view, dorsal view in thumbnail, show slit-openings of tracts on epigynal plate. (B) Inner view, without EF, arrows to FG opening on dorsal surface. (C) Inner view, with EF, arrows to internal openings of UE and SUE respectively. (D) Inner view, note thickened TD and translucent SUE full of sperm-like granules. (E) Posterior view. (F) Latero-inner view, arrow to broken SUE. (G) Inner view with UE removed from TD, arrows to external openings of UE and SUE respectively. (H) UE column, arrow to internal opening of UE. (I) Inner view, without EF. (J) Inner view, with EF, shows thickened TD and UE, arrow to translucent SUE. Color in blue represents CG/CD; orange, TD and UE; pink, FG/FD; red, spermatheca; yellow-green, SUE. BL, book lung; CD, copulatory duct; CG, copulatory groove; CO, copulatory opening; EF, epigastric furrow; FD, fertilization duct; FG, fertilization groove; S, spermatheca; SUE, *secondary uterus externus*; TD, transversal duct; UE, *uterus externus*. Scale bars: mm.

**Fig 2:**
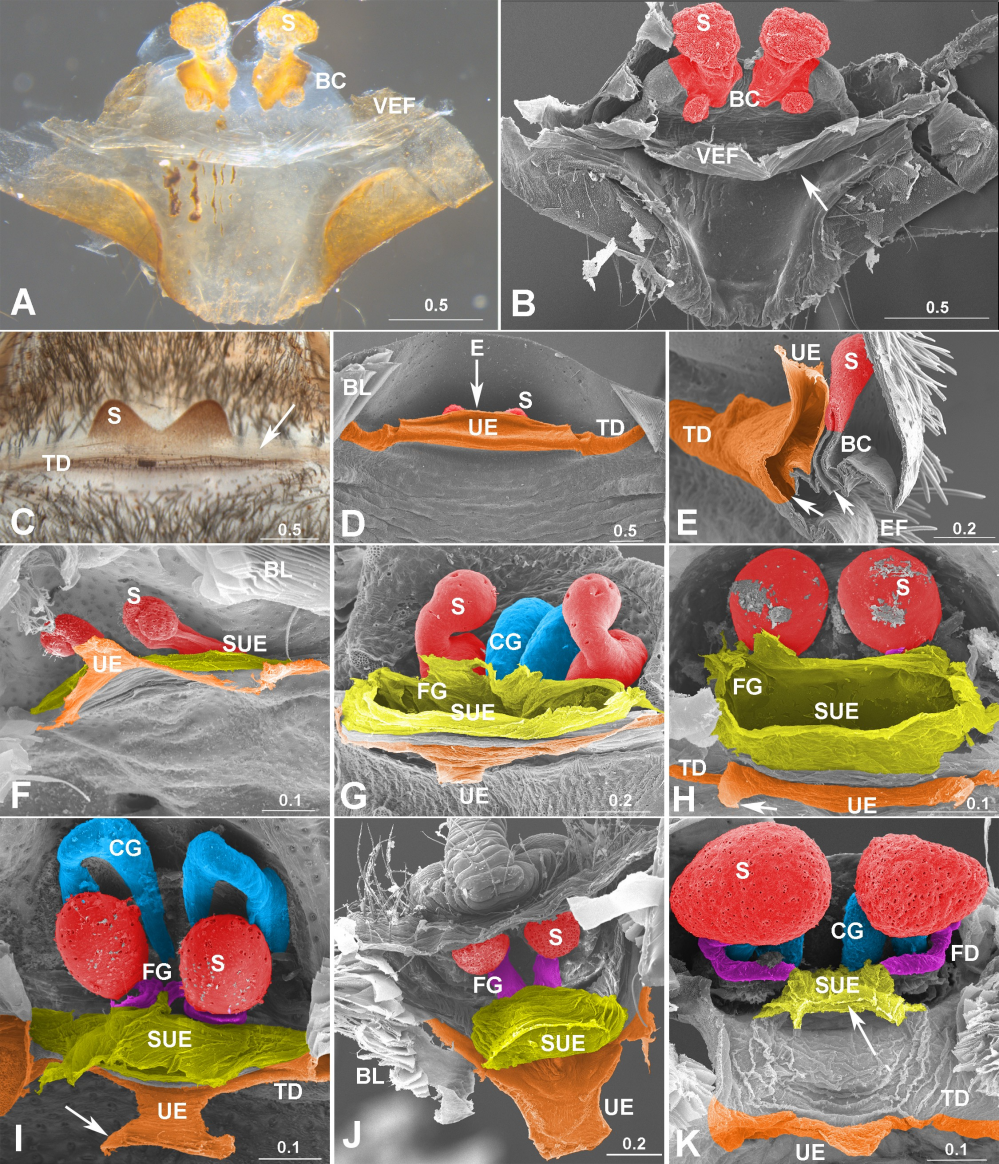
Variation in *uterus externus*. (A–B) *Songthela* sp. (Liphistiidae), EF removed, arrow in (B) to BC opening. (C–E) *Cyriopagopus* sp. (Theraphosidae). (C–D) Inner view, note wide UE arising from TD, arrow in (C) to translucent UE column, in (D) indicates section position of (E). (E) Longitudinal section, arrows to openings of UE and BC respectively in EF. (F) *Tricalamus* sp. (Filistatidae), haplogyne. (G) *Alopecosa licenti* (Lycosidae). (H) *Clubiona duoconcava* (Clubionidae). (I) *Zora* sp.1 (Miturgidae). (J) *Araneus diadematoides* (Araneidae). (K) *Nephila clavata* (Araneidae). Note UE column in (G–K) more or less reduced in entelegynes, arrows in (H) and (I) to muscle apodeme, in (K) to internal opening of SUE. Color in blue represents CG/CD; orange, TD and UE; pink, FG/FD; red, spermatheca; yellow-green, SUE. BC, bursa copulatrix; BL, book lung; CG, copulatory groove; EF, epigastric furrow; FD, fertilization duct; FG, fertilization groove; S, spermatheca; SUE, *secondary uterus externus*; TD, transversal duct; UE, *uterus externus*; VEF, ventral EF wall. Scale bars: mm.

**Fig 3:**
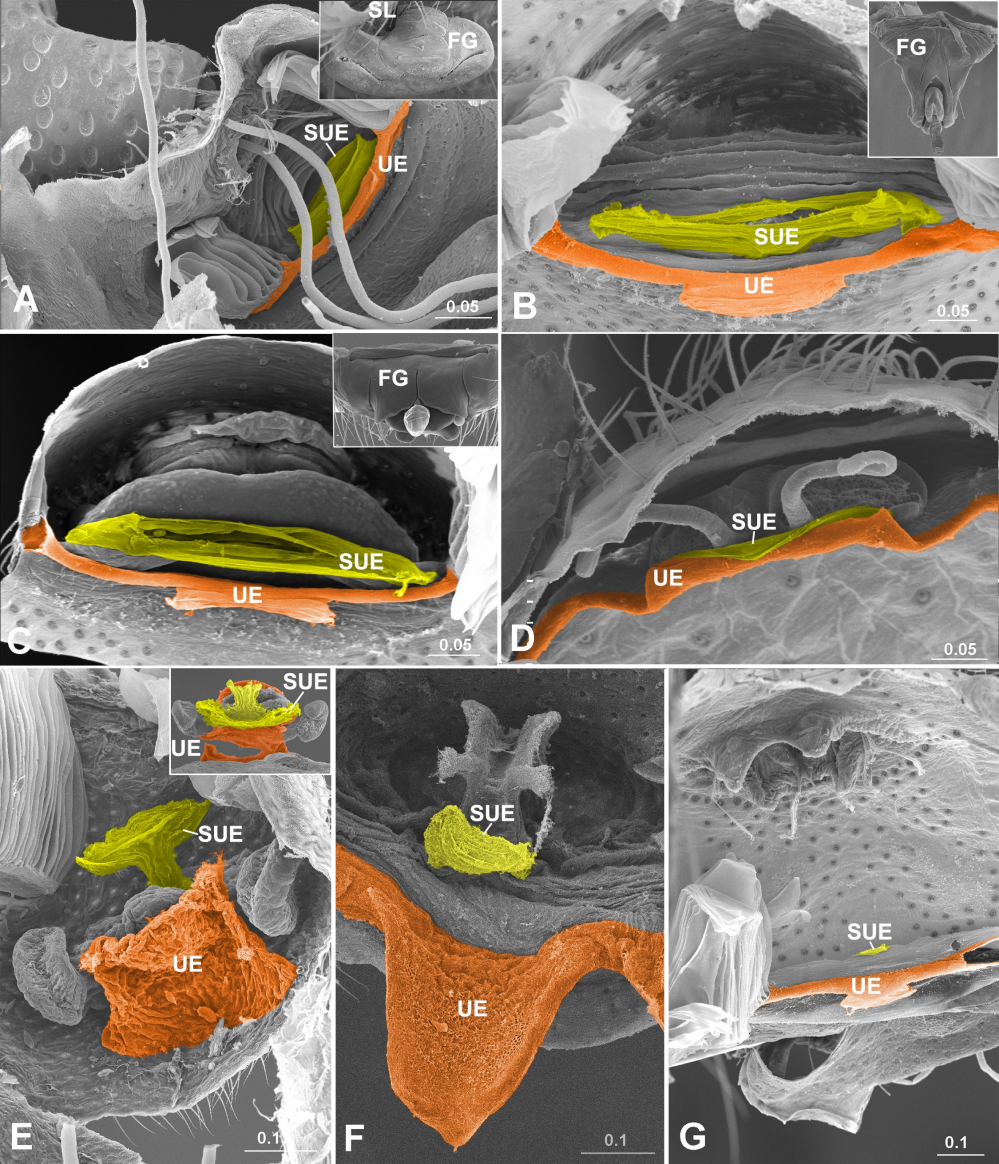
*Secondary uterus externus* in special cases. (A) *Solenysa protrudens* (Linyphiidae), thumbnail shows epigynum hung by a solenoid. (B) *Acanoides hengshanensis* (Linyphiidae), note wrinkled epigynal base makes epigynum movable; (C) *Tenuiphantes mengei* (Linyphiidae). Note thumbnails in (A-C) show FG slits on dorsal surface, not extending into EF. (D) undet. sp. (Ochyroceratidae), haplogyne. (E) *Pachygnatha degeeri* (Tetragnathidae), secondary haplogyne, EF lost. (F) *Argiope bruennichi* (Araneidae), juvenile with epigynal tracts under developed. (G) undet. sp. (Theridiidae), juvenile with epigynal tracts undeveloped. Color in orange represents TD and UE; yellow-green, SUE. EF, epigastric furrow; FG, fertilization groove; SL, solenoid; SUE, *secondary uterus externus*; UE, *uterus externus*. Scale bars: mm.

**Fig 4:**
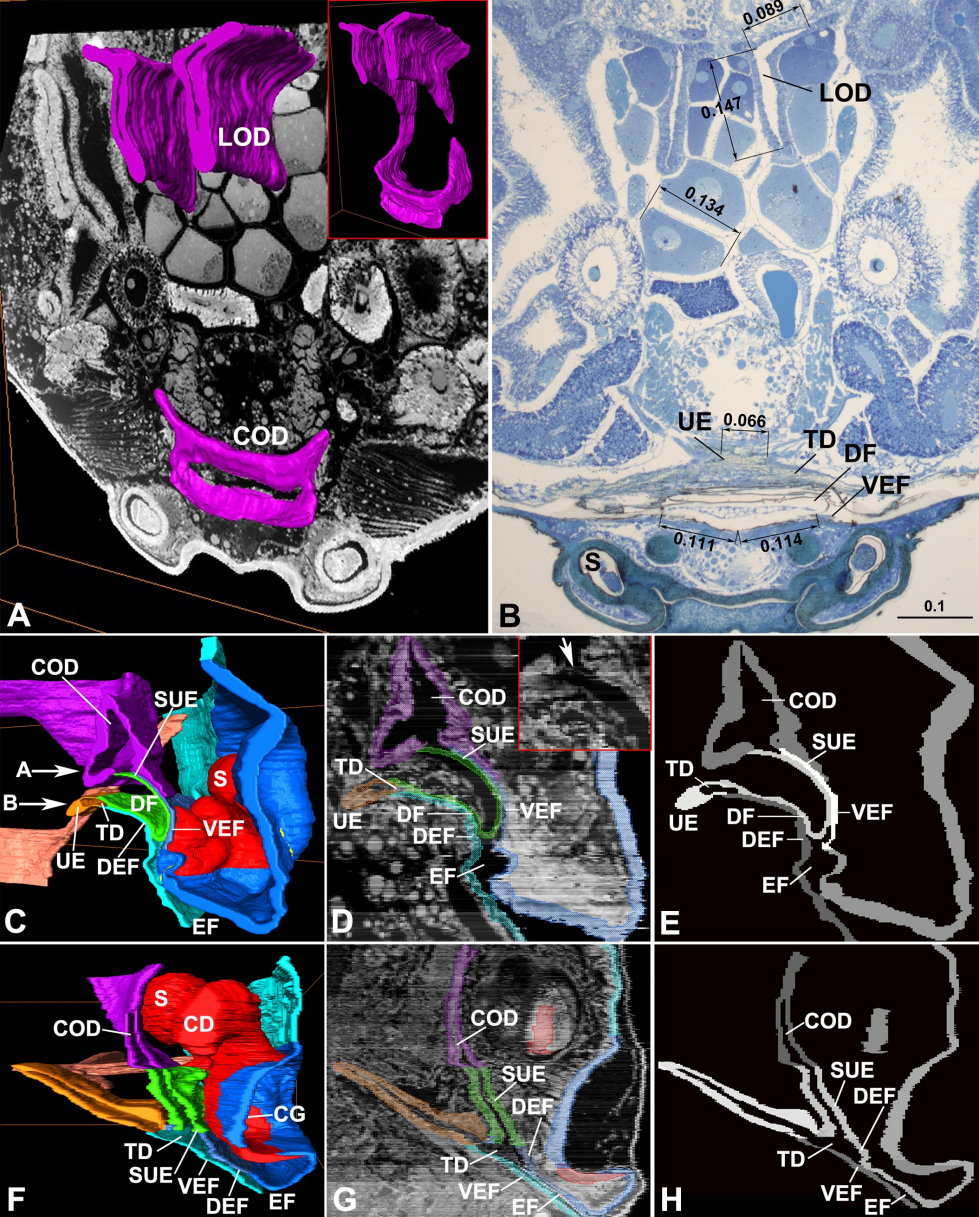
Connection between oviduct and epigastric furrow. (A–E) *Diphya wulingensi* (Tetragnathidae). (F–H) *Parasteatoda tepidariorum* (Theridiidae). (A) Reconstructed oviduct, section crossing posterior part of COD. (B) Section crossing UE and SUE, note the opening widths of UE and SUE, comparing to size of eggs. (C) Scenograph of longitudinal section, note DF between UE and SUE, arrows indicate section positions of (A) and (B) respectively. (D) Longitudinal section with structures lined in colors, arrow to internal opening of SUE. (E) Line drawing of (D). (F) Scenograph of longitudinal section, note UE extension divergent from COD. (G) Longitudinal section with structures lined in colors. (H) Line drawing of (G). Color in blue represents sclerotized epigynal plate; light blue, integument of abdomen and DEF; greyish blue, less sclerotized VEF; green, DF and SUE; orange, TD and UE; purple, oviduct; red, epigynal tract; flesh, muscles. CD, copulatory duct; CG, copulatory groove; COD, common oviduct; DEF, dorsal EF wall; DF, dorsal fold; EF, epigastric furrow; LOD, lateral oviduct; S, spermatheca; SUE, *secondary uterus externus*; TD, transversal duct; UE, *uterus externus*; VEF, ventral EF wall. Scale bars: mm.

**Fig 5:**
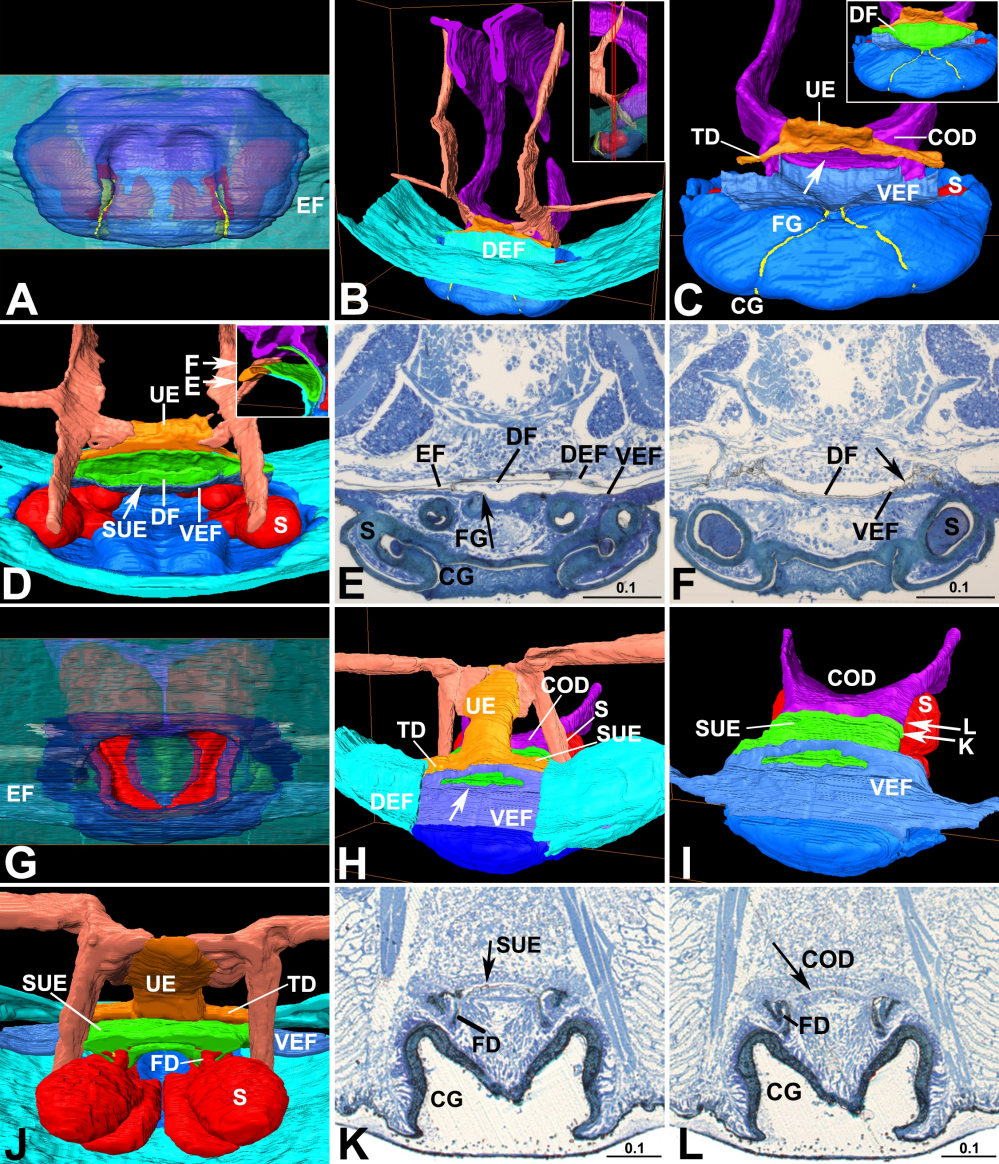
Reconstruction of oviduct in relation with epigynal tracts. (A–F) *Diphya wulingensi* (Tetragnathidae). (G–L) *Parasteatoda tepidariorum* (Theridiidae). (A) Ventral view. (B) latero-posterior view, lateral in thumbnail. (C) Posterior view with DEF and most part of VEF removed, with DF in thumbnail, arrow to COD opening. (D) Antero-dorsal view, longitude section of EF in thumbnail, note muscles attached to sides of UE end, up arrow to SUE opening, right arrows indicate section positions of (E) and (F). (E) Section crossing proximal part of FG, arrow to proximal end of FG. (F) Section crossing DF, arrow to two appressed cuticle layers of SUE. (G) Ventral view. (H) Latero-posterior view, with central part of DEF removed, note muscles attached to sides of UE end, arrows to UE opening on TD and SUE opening on VEF respectively. (I) Latero-posterior view with DEF and TD removed, note SUE and COD smoothly connected, longitude section of EF in thumbnail, arrows section positions of (K) and (L). (J) Antero-dorsal view. (K) Section crossing SUE, arrow to internal cuticle layer of SUE wall. (L) Section crossing COD, arrow to COD wall without internal cuticle layer. Color in blue represents sclerotized epigynal plate; light blue, integument of abdomen and DEF; greyish blue, less sclerotized VEF; green, DF and SUE; orange, TD and UE; purple, oviduct; red, epigynal tract; flesh, muscles. COD, common oviduct; DEF, dorsal EF wall; DF, dorsal fold; EF, epigastric furrow; S, spermatheca; SUE, *secondary uterus externus*; TD, transversal duct; UE, *uterus externus*; VEF, ventral EF wall. Scale bars: mm.

Our survey found a high variation in internal genital anatomy in entelegyne spiders (for overview of epigynal diversity across taxa, see S4 File), with the following points that these anatomies have in common. Almost all entelegyne exemplars had the epigastric furrow with two internal openings (Figs 1–3). One opening internally leads into the common oviduct, usually maintaining a close relation with the fertilization tracts (Figs 4 and 5). For reasons outlined below, we interpret this tract not to be the original *uterus externus*. The second internal opening leads into a dead-end caecum (Figs 4 and 5). As explained below, we interpret this caecum to be the original *uterus externus* (UE in figures).

The epigastric furrow is an integument fold with a transversal opening between the two spiracles of the two book lungs on the ventral surface of the abdomen (Fig 1C and 1E), extending somewhat forwards (Fig 4C). Its ventral wall is continuous with the epigynal dorsal surface, and its dorsal wall with the integument of the abdomen. The furrow’s bottom is thickened and internally lined by a mesh of cuticular extensions (Fig 1G), forming a transversal duct connecting the paired chambers of book lungs (Fig 1J). Such a mesh is absent in liphistiids and mygalomorphs (Fig 2E). Two cuticular structures are associated with the epigastric furrow (Fig 1C). One is *uterus externus*, a cuticular column protruding internally from the middle of the transversal duct, present in almost all the samples in the present study, including those having the epigastric furrow secondarily lost (Fig 3E). The other cuticular structure, a column similar to *uterus externus* arising from the furrow’s ventral wall, is termed *“secondary uterus externus”* (SUE in figures) that is found in the samples of all entelegynes and some haplogynes (Figs 2F and 3D), as well as juveniles (Fig 3F and 3G), but is absent in liphistiids and mygalomorphs (Fig 2E and S4 Fig). Both columns have an internal opening (Fig 1C), and externally open to the epigastric furrow (Fig 1G). The *uterus externus* wall is thick, as is that of the transversal duct, while that of *secondary uterus externus* is thin and translucent in LM pictures, and resembles that of the furrow’s ventral wall (Fig 1J).

These two cuticular columns have different relationships with fertilization tracts. In some taxa, the two fertilization ducts converge into a *common fertilization duct* sensu Berendonck & Greven [4], which is equivalent to the *secondary uterus externus* (Fig 1G). In most other taxa, those with a slit-like opening, fertilization groove slits extend into the epigastric furrow, and further into the *secondary uterus externus* (Fig 1A and 1C). No direct connection exists between fertilization tracts and *uterus externus* (for overview across taxa, see S4 File and S1 Fig). Even in liphistiids and mygalomorphs, the spermathecae arise from a bursa copulatrix (Fig 2A), which also opens to the furrow’s ventral wall, independent from the *uterus externus* (Fig 2E). In some special cases, neither of the two columns has a connection with fertilization tracts: e.g. in those where the epigynum hangs on an extensible tube (Fig 3A); in those species where the two fertilization grooves stop outside the epigastric furrow (Fig 3B and 3C); in those with a secondary haplogyne epigynum lacking fertilization tracts (Fig 3E; and Fig 38C within [14]); and in juveniles having undeveloped or underdeveloped epigynal tracts (Fig 3F and 3G). The detailed morphological data for taxa examined here is presented in S1 Table.

The *uterus externus* and the *secondary uterus externus* differ in structure. Although in liphistiids and mygalomorphs the *uterus externus*, as a wide column, has a large internal opening (Fig 2D), in Araneomorphae, both entelegyne spiders and some haplogynes, its internal opening is usually much smaller than that of the *secondary uterus externus* (Figs 1 and 2), even when it has no connection to fertilization grooves (Fig 3A–3C). The *uterus externus* shape varies greatly, with a smaller internal opening (Fig 2G), or is even closed (Fig 2K); as a column shaped (Fig 1C), or getting elongated and narrowed (Fig 1H), even reduced with a pair of apodemes left (Fig 2H and 2I). Our interpretations of LM (Fig 1D) and SEM (S1E Fig) images suggest that sperm are found in the *secondary uterus externus*, but never in the *uterus externus*.

For *Diphya* and *Parasteatoda* the 3D-reconstructed internal reproductive duct consists of a pair of ribbon-like lateral oviducts, converging into a common oviduct (Fig 4A). The lateral oviducts are Y-shaped in cross-section, and run forward among the eggs. At the epigastrium, they extend ventrally, forming a U-shaped common oviduct, which has a wide posterior opening. Furthermore, the measurements on the HSS image shows that the internal opening of *uterus externus* is much smaller than the diameter of a mature egg (Fig 4B).

The common oviduct in these two closely investigated species connects with *secondary uterus externus*, leaving *uterus externus* as a caecum and serving as muscle apodeme (Figs 4 and 5). Within the epigastric furrow of *Diphya*, a dorsal fold that arises from the furrow’s ventral wall (Fig 4C) covers the fertilization groove slits (Fig 5C). Its dorsal layer is connected with the furrow’s dorsal wall by the transversal duct, from which *uterus externus* protrudes; its ventral layer tightly closes the furrow’s ventral wall (Fig 5F), forming *secondary uterus externus*, which harbors the proximal parts of fertilization groove slits and internally opens to the common oviduct. The dorsal fold is filled with soft tissue that separates the two columns (Fig 4D). The common oviduct is triangular shaped in cross-section, having the two posterior angles extend toward the *uterus externus* and *secondary uterus externus*, respectively; however, only the one to the *secondary uterus externus* is opened, while the one to the *uterus externus* is blocked (Fig 4C–4E). In *Parasteatoda* the paired fertilization ducts converge into the *secondary uterus externus* (Figs 1G and 5J), which externally opens to the furrow’s ventral wall, close to the *uterus externus* opening (Fig 5H and 5I). The common oviduct smoothly connects with the internal opening of the *secondary uterus externus* (Fig 5I). The cross-sections show that its inner surface is lined with a thin cuticle layer (Fig 5K), while that of the common oviduct is not (Fig 5L). Furthermore, in both species paired groups of muscles attach to the sides of *uterus externus* end, but that of *secondary uterus externus* are free (Fig 5B and 5H).

Liphistiids and mygalomorphs lack the *secondary uterus externus* entirely (Fig 2A–2E). The wide and flat *uterus externus* column protrudes from the transversal duct, extends internally and then turns upwards. The sections of micro computed tomography (MCT) indicate that in the liphistiid *Songthela* sp., the upwards-turning *uterus externus* connects to the common oviduct, with paired groups of muscles attaching to its side column (S4 Fig). In addition, the spermathecae in liphistiids and mygalomorphs, arise on the roof of bursa copulatrix located dorsal to the *uterus externus*. Each spermatheca opens independently to the bursa copulatrix, which opens on the ventral wall of the epigastric furrow (Fig 2E and S4 Fig).

## Discussion

The novelty of our study is the demonstration of two openings at the epigastric furrow in entelegyne spiders, both in adults and juveniles, as well as in some haplogyne spiders. We homologize the column protruding internally from the transversal duct as the *uterus externus* because a comparable structure is also present in liphistiids and mygalomorphs and is thus the plesiomorphic condition in spiders. However, countering prior knowledge, we showed that this organ does not function in egg laying in entelegynes. Instead, oviducts in entelegyne spiders connect to the *secondary uterus externus* rather than to the *uterus externus*, as evidenced by 3D-reconstructions in *Diphya* and *Parasteatoda*. Furthermore, fertilization ducts (in fact, commonly, grooves), do not have contact with the *uterus externus*, but are instead usually associated with the *secondary uterus externus*. A common oviduct opening to the epigastric furrow via the *uterus externus* has been thought to be present universally in spiders; however, it seems to be present in the relatively basal spider lineages, including Mesothelae, Mygalomorphae, and some Araneomorphae with haplogyne genitalia, but not in entelegynes.

Our interpretation that the dead-end ceacum (Fig 4C and 4F) in entelegyne spiders is homologous to the *uterus externus* present in liphistiids and mygalomorphs differs from literature accounts [1–4,6,7,11]. This interpretation is evidenced by the common feature of all spiders, namely that the *uterus externus* protrudes internally from the transversal duct and serves as a muscle apodeme. Although the *uterus externus* in over two thirds of spiders examined here was typically not column shaped (see S1 Table), it in all cases protruded internally from the transversal duct. This feature is commonly recognized as a muscle apodeme (Figs 2 and 3; see also [9,11,15]. Accordingly, these two features are common in entelegynaes and haplogynaes, and even shared with liphistiids (S4 Fig) and mygalomorphs (Fig 2E), the two basalmost spider lineages [16–18]. However, in both liphistiids and mygalomorphs, as well as in some haplogyne araneomorphs (Figs 2A, 2B, 2J, 3D within [11]; Fig 4A within [13]; Fig 7 within [19]), only the *uterus externus* column protrudes internally from the epigastric furrow. The liphistiid *uterus externus* column turns upwards to connect to the common oviduct, indicating a function in eggs laying. This is hypothesized to be the original function of any *uterus externus*, which subsequently became a dead-end ceacum that serves as muscle apodeme in most araneomorphs.

On the other hand, a large, membranous column that takes over the function of a *uterus externus* in most spiders must be a non-homologous, secondary *uterus externus*. This structure is absent in liphistiids, mygalomorphs, and some araneomorphs with haplogyne epigyna. Where present, the *secondary uterus externus* arises from the ventral wall of the epigastric furrow whereas the *uterus externus* arises from the transversal duct, although it varies in shape and level of reduction (Figs 1–3). The *secondary uterus externus* in entelegyne spiders is usually associated with the fertilization tracts (Figs 1C, 1G and 2G–2K), a feature that is absent in Mesothelae [20], Mygalomorphae [21], and those Araneomorphae with haplogyne genitalia [1]. Special cases, such as the epigyna with fertilization groove slits that stop outside the epigastric furrow, no relation with the *secondary uterus externus* in some linyphiid groups (Fig 3A–3C), and the secondary haplogyny in the otherwise entelegyne family Tetragnathidae (Fig 3E), are all derived conditions [10,22,23].

We thus hypothesize that a novel entelegyne organ, the *secondary uterus externus*, has taken over egg laying, the original function of the plesiomorphic *uterus externus*. Although both of them generally coexist in entelegynes (Figs 1–3), their anatomical details suggest that eggs are released through the *secondary uterus externus*, while the *uterus externus* has no such function. Generally, the transversal common oviduct has a wide opening matched by the large internal opening of the *secondary uterus externus* (Figs 4A, 5D and 5I). Similarly, a wide opening of *uterus externus* is found in liphistiids and mygalomorphs (Fig 2D and S4 Fig), while that in entelegynes and most haplogynes is much smaller (Figs 1–3). Various degrees of *uterus externus* reduction on the one hand, and *secondary uterus externus* functional morphology on the other, imply that the latter has a function in egg laying in most spider groups. If this is true, then such a functional replacement would imply a connection shift with the common oviduct from the *uterus externus* to the *secondary uterus externus*. Such a connection shift needs to be hypothesized with some imagination, but may be plausible considering the shape of the common oviduct folds in *Diphya* (Fig 4C), these folds come into close proximity with both the *uterus externus* and the *secondary uterus externus*.

The factors that drive such a hypothetical shift should be related to egg-laying. The *uterus externus* opening in entelegynes, if even present, is much smaller than the diameter of mature eggs (Fig 4B). Although muscles that attach to the sides of *uterus externus* end (Fig 5D and 5H) could possibly expand its opening [15], the thick wall of its column do not seem to be extensible, as they resemble the transversal duct and book lung layers in entelegynes (Fig 1C and 1G). In contrast, the *secondary uterus externus* has a large opening, and is formed by thinner, less sclerotized integument (Fig 1D and 1J) that is presumably elastic. These anatomical differences suggest that the origin of *secondary uterus externus* may have facilitated discharge of larger eggs.

The functional takeover of *secondary uterus externus* from *uterus externus* may also affect fertilization, as it creates extra space for internal fertilization. However, the precise site of fertilization in entelegyne spiders remains elusive. Although the *secondary uterus externus* anatomical details allow for eggs to meet sperm before oviposition, it is not rare that fertilization tracts have no direct relation with the *secondary uterus externus*, even without extending into the epigastric furrow, e.g. [19,20,24,25]. This implies that fertilization could also plausibly be external [26]. Sperm expulsion is likely caused by glands whose secretions displace the sperm mass [1,27,28]. Often it has been observed that the laid-out eggs are surrounded by a clear, viscous liquid [1]. Perhaps sperm is to be found within this liquid, whose function may go beyond only cementing the eggs together. If so, a likely place of fertilization in some spiders could be the egg sac where sperm and eggs are well protected [1]. Indeed, in entelegynes fertilization could start internally and be completed externally.

## Conclusions

As we demonstrate with detailed morphological comparisons, the fertilization tracts in entelegyne spiders, regardless of their groove or duct state, have no direct connection with the *uterus externus*, but usually are connected to the *secondary uterus externus*, which may have taken over the function of the plesiomorphic *uterus externus*. Future studies should test the precise site of internal versus external fertilization, which we hypothesize both to be likely in certain spider groups, or perhaps operate in combination. The egg sac, being made of special silk whose function may go beyond mere physical protection of the eggs, could be a likely venue of external fertilization in at least some spider groups.

## Materials and methods

### Taxon sampling

First, to examine connection between epigynal tracts and the *uterus externus*, epigyna were broadly sampled to maximize phylogenetic representations and to emphasize variation in the anatomy of the fertilization tracts. Spiders of the family Linyphiidae were sampled more intensively due to their great epigynal diversity, especially the fertilization tracts (for collecting data of the materials examined here, see S2 Table), with some legacy SEM images [9,11,13,14,29,30]. To demonstrate the relationship between fertilization tracts and the *uterus externus*, epigyna were dissected together with the entire epigastric furrow from the abdomen. Furthermore, two species, *Diphya wulingensis* and *Parasteatoda tepidariorum*, were selected as representatives of those groups with fertilization tracts in a groove and duct state, respectively, for a further detailed histological study. Semithin HSS images were collected from two individuals per species.

### Morphological examination

All materials were examined using a Leica M205A stereomicroscope and scanning electronic microscope (SEM). Light microscopic (LM) pictures were collected using a Leica DFC 500 camera. For SEM examination, specimens were prepared as described in [31]. Non-chitinous abdominal tissues were digested with Pancreatin (Sigma LP 1750) enzyme complex, then cleared by an ultrasonic cleaner before drying. SEM images were taken using a LEO 1430VP in the Department of Biological Sciences at George Washington University and a Hitachi S-3400N at China Agricultural University. The semi-thin serial sections (1μm) were applied to the spider opisthosoma and performed at China Agricultural University using a Leica EM UC6 microtome with a glass knife and stained with toluidine blue (1%) in an aqueous borax solution (1%) at approximately 90°C for 1–4 minutes. All slides were examined using a Leica DM5500B light microscope and images were collected with a Leica DFC 500 camera. The egg diameter, and the opening widths of the *uterus externus* and the *secondary uterus externus* were measured using the measurement tool of the software package LEICA APPLICATION SUITE to qualitatively compare the size of a mature egg and that of the uterus externus opening and secondary uterus externus opening. One of the two sets of HSS images per species was selected for 3D-reconstruction. The 3D-reconstructed structures for *Diphya* and *Parasteatoda* were based on images of 237 and 469 semithin HSS slices, respectively (S5 and S6 Figs). The MCT approach was applied to the spider opisthosoma of *Songthela* sp. (Liphistiidae) and performed using MagicEye-MicroCT225 at 40 kV, 300.0 muA in Capital Normal University.

Data were visualized and processed using the 3D analysis software Avizo 9.0. The values used to build graphs for *Diphya wulingensis* include: Input resolution (px): 1700*1700*237; Input voxel size: 1*1*2.37; Filter: Lanczos; Mode: dimensions; Resolution (px): 480*480*237; Voxel size: 3.54167*3.54167*2.37. For *Parasteatoda tepidariorum*: Input resolution (px): 1200*1500*469; Input voxel size: 1*1*1; Filter: Lanczos; Mode: dimensions; Resolution (px): 320*400*469; Voxel size: 3.75002*3.75001*1. For *Songthela* sp.: Resolution (px): 960*960*768; Voxel size: 5.37 μm. The main points extracted from HSS MCT images include epigynal tracts, epigastric furrow, *uterus externus*, *secondary uterus externus*, oviduct, and the muscles attached to the *uterus externus* end. All this structures were lined in color on the black-and-white reversal images in Avizo.

## Supporting information

S1 File Methodological overview. (DOCX)

S2 File Basic conformation of female reproductive anatomy in spiders. (DOCX)

S3 File Summary on the relationships among fertilization ducts, uterus externus and oviduct in the literature. (DOCX)

S4 File Description of epigynal types. (DOCX)

S1 Table Morphological features of UE, SUE and epigynal tracts. EB, epigynal base; NEB, normal EB, with epigynum attached on the abodmen; WEB, wrinkled EB that makes epigynum movable. UE, uterus externus. SUE, secondary uterus externus; CF, common fertilization duct; PCF, pseudo common fertilization duct; SUEC, column of SUE without direct connection with FT. FT, fertilization tract; FG, FT in groove state; FD, FT in duct state; AFD, additional fertilization duct; PFD, pseudo fertilization duct. FG ending, proximal end of FG; IEF, FG slits extend into EF; MEF, FG slits stop at the margin of EF; OEF, FG slits stop outside EF. EF, epigastric furrow; EF fold, integument fold/folds arising from EF ventral wall; DF, dorsal fold; LF, lateral fold; DF+LF, one DF and a pair of LF; IF, internal fold anterior to FG; DF+IF, DF externally and LF inside epigynum.
(XLSX)

S2 Table Collecting data of materials examined in the present study. (XLSX)

S1 Fig Epigynal types. (A–C) *Pardosa chionophila* (Lycosidae), Type-I FG. (D–E) *Agyneta* sp. (Linyphiidae), Type-II FG+AFD, arrow to sperm-like granules in SUE. (F–H) *Parasteatoda tepidariorum* (Theridiidae), Type-III FD. (I–K) *Nephila clavata* (Araneidae), Type-IV PFD. Scale bars: mm.
(TIF)

S2 Fig Serial cross sections of *Diphya wulingensis* (Tetragnathidae). (A) Longitudinal section with structures lined in colors. (B) Line drawing of (A), square shows corresponding part in detail, lines indicate sections positions in (C-K), numbers refer to relevant numbers of slices. (C-E) Sections crossing COD. (F-I) Sections around UE internal end, arrows to muscles attached to sides of UE end. (J-K) Sections crossing EF and DF. COD, common oviduct; DEF, dorsal EF wall; DF, dorsal fold; EF, epigastric furrow; SUE, *secondary uterus externus*; TD, transversal duct; UE, *uterus externus*; VEF, ventral EF wall.
(TIF)

S3 Fig Serial cross sections of *Parasteatoda tepidariorum* (Theridiidae). (A) Longitudinal section with structures lined in colors. (B) Line drawing of (A), square shows corresponding part in detail, lines indicate section positions in (C-K), numbers refer to relevant numbers of slices. (C-G) Sections around UE internal end, arrows to muscles attached to sides of UE end. (H-K) Sections around UE and SUE external openings. COD, common oviduct; DEF, dorsal EF wall; DF, dorsal fold; EF, epigastric furrow; SUE, *secondary uterus externus*; TD, transversal duct; UE, *uterus externus*; VEF, ventral EF wall.
(TIF)

S4 Fig Connection between oviduct and epigastric furrow in *Songthela* sp. (Liphistiidae). **(**A) Abdomen, ventral view, line shows section position of (D). (B) Longitudinal section with angle, section position shown in (D). (C) Detail of B, lines show section positions of (D-F). (D-F) Cross sections with angle. (D) Section crossing UE and left apodeme. (E) Section crossing turning point of UE. (F) Section crossing right apodeme and EF, note UE as a wide column with narrow and long chamber, and a pair of apodemes located at lateral sides of UE. (G-I) Longitudinal sections with angle, section positions shown in (D). (G) Section crossing left apodeme and EF. (H) Section crossing COD, UE and EF, note UE protruding internally from EF bottom, turning upwards to connect to COD (shadow). (I) Same section with reconstructed COD, UE, EF and right apodeme and muscles, BC opening to VEF (arrows). BC, bursa copulatrix; BL, book lung; COD, common oviduct; EF, epigastric furrow; M_1_, left bundle of muscles; M_2_, right bundle of muscles; S, spermatheca; UE, *uterus externus*; VEF, ventral EF wall.
(TIF)

S5 Fig Figure set of HSS slices of *Diphya wulingensis*. (ZIP)

S6 Fig Figure set of HSS slices of *Parasteatoda tepidariorum*. (ZIP)
