# Supplementary material for: Comparative morphology refines the conventional model of spider reproduction
Source: PLoS One. 2019 Jul 5;14(7):e0218486. doi: 10.1371/journal.pone.0218486 (PMC6611574; doi:10.1371/journal.pone.0218486)
Supplement: S1 File — (DOCX) [file pone.0218486.s001.docx]

**Additional file 1:** File S1. Methodological overview

Traditional approaches used in morphological study, light microscopy (LM) and scanning electronic microscopy (SEM), fail to demonstrate the relationship between the proximal end of epigynal tracts and the internal reproductive duct. As a species rich group, spiders have species-specific genitalia, both for male palp and epigynum (the genitalia of female entelegyne spiders), while the internal reproductive duct is conservative. In conventional taxonomic practice, the knowledge on epigynal characters is largely based on LM approach by examining slice-mounted epigyna under light microscope that is capable of revealing the epigynal features, tract tracings, degree of sclerotization, and transparency of the cuticle (Millidge 1984,1993; Ma et al. 2016; Wipfler et al. 2016). The main limitations of this technique are: 1) the epigynum has to be dissected from the spider abdomen, that destroys the possible connection between fertilization ducts and internal reproductive duct; 2) the study material has to be cleared in the process of treatment that makes the structures formed by less sclerotized integument and soft tissues indiscernible; 3) some information is lost, such as the three-dimensional relationships among structural elements. The SEM approach is an excellent tool for documenting the exoskeleton structures (Friedrich et al. 2014; Wipfler et al. 2016). Treated by enzymes during the material preparation to digest the surrounding soft tissues (Álvarez-Padilla & Hormiga 2008), the epigynal morphology can be demonstrated both externally and internally by SEM images with a high resolution (e.g. Sierwald 1989; Dimitrov et al. 2007; Tu & Hormiga 2010; Álvarez-Padilla & Hormiga 2011). Nevertheless, the internal reproductive duct is also digested during the preparation process, except for the distal part referred as *uterus externus* (e.g. Griswold et al. 2005; Cabra-García et al. 2014; Pérez-González et al. 2016). Accordingly, neither approach can demonstrate epigynal tracts and internal reproductive duct simultaneously.

Our current knowledge regarding the two fertilization ducts connecting to the uterus externus is largely inferred from histological serial sections (HSS) and usually presented schematically in literature (Bhatnagar & Rempel 1962; Eberhard & Huber 1998; Berendonck & Greven 2005; Uhl et al. 2010; Foelix 2011), as well as in the textbooks (e.g. Hickman et al. 2008), rather than direct demonstration. Because fertilization ducts opening to the uterus externus is thought to be common in spiders (Foelix 2011) and due to some misunderstanding on epigynal tracts (Tu & Hormiga 2010), the studies on epigynal morphology usually pay little attention to the relationship between the epigynal tracts and the internal fertilization duct. Almost in all existing literature, the images of epigynal morphology lack the information on the proximal ends of fertilization ducts, even in the studies via 3D-reconstruction based on HSS pictures (e.g. Berendonck & Greven 2005; Schendel et al. 2018, but see Tu & Hormiga 2010).

**Reference**

Hickman CP, Roberts LS, Keen SL, Larson A, I’Anson H, Eisenhour DJ. *Integrated Principles of Zoology*. 14th ed.,Reidy PE, ed.. McGraw-Hill, a business unit of The McGraw-Hill Companies, Inc., 1221 Avenue of the Americas, New York, NY 10020; 2008.
