# Supplementary material for: Comparative morphology refines the conventional model of spider reproduction
Source: PLoS One. 2019 Jul 5;14(7):e0218486. doi: 10.1371/journal.pone.0218486 (PMC6611574; doi:10.1371/journal.pone.0218486)
Supplement: S2 File — (DOCX) [file pone.0218486.s002.docx]

**Additional file 2:** **File S2. Basic conformation of female reproductive anatomy in spiders**

According to Foelix (2011), the gonopore of female spiders is situated inside the epigastric furrow, an integument groove transversely located on the ventral surface of the abdomen between the two spiracles of book lungs. The distal part of the oviduct referred as the *uterus externus* opens at the furrow’s bottom. The female genitalia are located on ventral surface of the abdomen above the epigastric furrow; the sperm coming from males are deposited in the spermathecae, the openings of which are located within the uterus externus. Such a conformation facilitates eggs to meet sperm during oviposition and to be fertilized when they are laid out.

Two types of female genitalia are recognized in spiders, haplogyne and entelegyne. In the haplogyne type, the spermathecae have a single opening to the uterus externus; the sperm enters and leaves via the same duct. In the entelegyne type, the paired spermathecae have two openings: the entrances are connected with copulatory openings by paired copulatory ducts for sperm uptake, and the exits are connected to the uterus externus by paired fertilization ducts for sperm discharge.

A basic structural model of an entelegyne is widely accepted (e.g. Foelix 2011: fig. 7.13; Uhl et al. 2010: fig. 2). Two terms, “epigynum” and “vulva”, have been used to refer to the external sclerotized plate on which the copulatory openings located and the internal ducts including the spermathecae, respectively (e.g. Sierwald 1989; Eberhard & Huber 1998). The studies of the great diversity of entelegyne structures is largely focused on variation on epigynal plate and tracings of copulatory ducts driven by sexual selection (Arnqvist 1998; Hosken & Stockley 2004; Eberhard 2004, 2009); characters of fertilization ducts are comparatively conservative due to their single function. The direct opening of the fertilization ducts to the uterus externus is inferred based on histological serial sections (e.g. Bhatnagar & Rempel 1962; Eberhard & Huber 1998; Berendonck & Greven 2005). This is thought to be common for spiders (Foelix 2011), and usually absent in studies of genital morphology due to methodological limitations (File S1). In fact, the direct connection between fertilization ducts and the uterus externus has never been unequivocally demonstrated, apart from schematic depiction (see summary in File S3).

**References**

Arnqvist G. Comparative evidence for the evolution of genitalia by sexual selection. *Nature*. 1998;393:784-787.

Eberhard WG. Why study spider sex: special traits of spiders facilitate studies of sperm competition and cryptic female choice. *J Arachnol*. 2004;32(3):545-556.

Eberhard WG. Evolution of genitalia: Theories, evidence, and new directions. *Genetica*. 2009;138(1):5-18. doi:10.1007/s10709-009-9358-y.

Hosken DJ, Stockley P. Sexual selection and genital evolution. *TRENDS Ecol Evol Vol19*. 2004;19(2):87-93. doi:10.1111/aen.12053.
