# Supplementary material for: Comparative morphology refines the conventional model of spider reproduction
Source: PLoS One. 2019 Jul 5;14(7):e0218486. doi: 10.1371/journal.pone.0218486 (PMC6611574; doi:10.1371/journal.pone.0218486)
Supplement: S3 File — (DOCX) [file pone.0218486.s003.docx]

**Additional file 3:** **File S3. Summary on the relationships among fertilization ducts, *uterus externus* and oviduct in the literature**

A number of studies addressed on epigynal morphology and its relationship with the oviduct by histological methods (e.g. Bhatnagar & Rempel 1962; Eberhard & Huber 1998; Berendonck & Greven 2005), concluded that fertilization ducts open to the *uterus externus*. Their results are represented by schematic drawings (Bhatnagar & Rempel 1962: fig. 53; Eberhard & Huber 1998: fig. 15; Berendonck & Greven 2005: fig. 14; see also Foelix 2011: fig. 7.12). Nevertheless, in their schematic drawings except for the *uterus externus* they recognized, there is another caecum protruding internally from the furrow’s bottom; none of them mentioned what it is, and where it connects.

Such a connection between fertilization ducts and uterus externus is questioned. In *Latrodectus curacaviensis*, Bhatnagar and Rempel (1962) found “two small and narrow tubes which open straight into the *vagina* on the dorsal side of the spermathecae”. A similar situation was found in *Leucauge mariana* by Eberhard & Huber (1998). However, in *Latrodectus revivensis*, Berendonck and Greven (2005) found that two fertilization ducts convergent into a “common fertilization duct”. They further concluded that fertilization ducts do not directly open to *uterus externus*, but via a common fertilization duct, although they did not demonstrate a connection between this duct and *uterus externus*. The species they studied belongs to the same genus (*Latrodectus*) as the species studied by Bhatnagar and Rempel (1962), and has very similar epigynal morphology. This suggests that the “*vagina*” recognized by Bhatnagar & Rempel (1962) is the “common fertilization duct” reported by Berendonck & Greven (2005).

Although it is commonly believed that fertilization ducts discharge the sperm into the uterus externus, the actual site where fertilization takes place remains unknown. Suzuki (1995) provided an evidence of internal fertilization in *Achaearanea tepidariorum*. Morishita et al. (2003) and Alberti & Michalik (2004) suggest that this observation could not be confirmed in other spiders since they fail to find sperm within uterus externus and internal reproductive duct. Sekiguchi (1988) hypothesized that fertilization may be completed externally.

Increasing evidence shows that fertilization tracts may have no direct connection with the *uterus externus*. In many groups, the ducts recognized under a microscope are in fact grooves, with slits opening on the epigynal plate (Tu & Hormiga 2010; see also Engelhardt 1910; Blauvelt 1936; van Helsdingen 1969; Saaristo 1971, 1972, 1977; Schendel et al. 2018; Uhl & Gunnarsson 2001). Moreover, the epigynal tracts in many spiders are in groove state from the beginning to the end (Tu & Hormiga 2010: fig. 3b) thus integrating the “epigynum” with “vulva” (referred to hereafter as a “groove-model epigynum”); in other cases, at least part of the tracts are in a duct state (referred hereafter as a “duct-model epigynum”). In groove-model epigyna, fertilization groove slits extend forward into the epigastric furrow along the epigynal dorsal surface (Tu & Hormiga 2010: fig. 2b). Although the proximal parts of fertilization grooves usually broken as they were dissected from the spiders’ abdomen, we really found in some linyphiids that the fertilization grooves stop outside the furrow (Tu & Hormiga 2010: fig. 1d), even in some linyphiids the epigynum hangs at the distal end of a extensible tube that makes the epigynum movable (Tu & Hormiga 2011), while the uterus externus opening is located at the furrow bottom (Foelix 2011). These observations suggest that, at least in the groove-model epigyna, if no additional duct is developed to introduce the sperm internally, those fertilization tracts in groove state may not release the sperm directly into the *uterus externus*.

**References**

Blauvelt HH. The comparative morphology of the secondary sexual organs of *Linyphia* and some related genera, including a revision of the group. *Festschrift Embrik Strand*. 1936;2:81-171.

Engelhardt V Von. Beiträge zur Kenntnis der weiblichen Copulationsorgane einiger Spinnen. *Zeitschrift für Wissenschaftliche Zool*. 1910;96:32-117.

Saaristo MI. Revision of the genus *Maro* O. P.-Cambridge (Araneae, Linyphiidae). *Ann Zool Fennici*. 1971;8:463-482.

Saaristo MI. Redelimitation of the genus *Oreonetides* Strand 1901 (Araneae Linyphiidae) based on an analysis of the genital organs. *Ann Zool Fennici*. 1972;9:69-74.

Saaristo MI. Secondary genital organs in the taxonomy of *Lepthyphantinae* (Araneae, Linyphiidae). *Reports from Dep Zool Univ Turku*. 1977;5:1-16.

Schendel V, Junghanns A, Bilde T, Uhl G. Comparative female genital morphology in *Stegodyphus* spiders (Araneae: Eresidae). *Zool Anz*. 2018:1-10.

doi:10.1016/j.jcz.2018.01.011.

Uhl G, Gunnarsson B. Female genitalia in *Pityohyphantes phrygianus*, a spider with a skewed sex ratio. *J Zool London*. 2001;255:367-376. doi:10.1017/S0952836901001467.

van Helsdingen P. A reclassification of the species of *Linyphia latreille* based on the functioning of the genitalia (Araneida, Linyphiidae), Part I. *Linyphia latreille* and *Neriene* Blackwall). *Zool Meded*. 1969:1-302.
