# Supplementary material for: Comparative morphology refines the conventional model of spider reproduction
Source: PLoS One. 2019 Jul 5;14(7):e0218486. doi: 10.1371/journal.pone.0218486 (PMC6611574; doi:10.1371/journal.pone.0218486)
Supplement: S4 File — (DOCX) [file pone.0218486.s004.docx]

**Additional file 4:** **File S4. Description of epigynal types** (Fig. S1)

The fundamental structure of entelegyne epigyna consists of a pair of epigynal tracts, located in front of the epigastric furrow. The spermatheca as an inflation on each tract divides the tract into two parts: the distal copulatory tract with opening on epigynal surface, and the proximal fertilization tract, the proximal end or which are usually broken by classical dissections (Fig. 1B, 1F). Epigynal tracts are of the two main states: “groove” (Fig. 1A) and “duct” (Fig. 1G). Unlike a duct as a closed circle in its cross section, the tract in groove state has a slit opening on the epigynal surface and is shaped as a comma in cross-section (Fig. 4E). Epigynal tracts in most spiders are grooves in their entirety; some are entirely ducts, or groove and duct in part. The integument surrounding the copulatory openings and groove slits are sclerotized to form the *epigynal plate* (Figs 1A, 4A). Its posterior part passes from ventral to dorsal side, continuing with the ventral wall of the epigastric furrow (Fig. 3C).

Copulatory tracts and fertilization tracts function in copulation and fertilization respectively (File S2). The great epigynal diversity is largely caused by modifications of the epigynal plate and variation of the tracings of copulatory tracts. Comparatively, fertilization tracts have a more conservative structure; variation largely focuses in the architecture of the tracts. Four states were recognized for fertilization tracts in entelegyne spiders: fertilization groove (FG), additional fertilization duct (AFD), fertilization duct (FD) and pseudo fertilization duct (PFD). Since the present study focuses on the relationship among fertilization tracts, uterus externus, and oviduct, we recognize four types of entelegyne epigyna with a special reference to the states of fertilization tracts.

Type I. FG. The fertilization tracts are entirely in a groove state (Fig. S1A­-C). Generally the proximal parts of fertilization grooves extend into the epigastric furrow and further into the *secondary* *uterus externus*. In some linyphiid groups, fertilization grooves remain outside the furrow (Fig. S2).

Type II. FG+AFD. Besides fertilization grooves, the epigyna have a pair of additional fertilization ducts, arising at the spermathecal junction (Fig. S1D-E). In those having an epigynum of Type II, fertilization grooves are kept within the epigynal cavity, a sigmoid folded scape covers over the groove slits; the additional fertilization ducts open to the ventral wall of the epigastric furrow.

Type III. FD. The fertilization tracts are in a duct state (Fig. S1F-H). The two fertilization ducts convergent into the *secondary* *uterus externus*, which opens to the ventral wall of the epigastric furrow.

Type IV. PFD. The fertilization tracts are in a groove state entirely or in part, but their groove slits are covered by an integument fold, or folds that make them look like ducts. We call them *“pseudo fertilization ducts”* (Fig. S1I-K).

In all entelegynes examined in the present study fertilization tracts, regardless in groove or duct state, have no direct connection with the *uterus externus*. However, they usually have their proximal parts included within the *secondary* *uterus externus*, except for some special cases (Fig. S2).
