# Supplementary figures and images for: Comparative morphology refines the conventional model of spider reproduction (part 1 of 5)
Source: PLoS One. 2019 Jul 5;14(7):e0218486. doi: 10.1371/journal.pone.0218486 (PMC6611574; doi:10.1371/journal.pone.0218486)

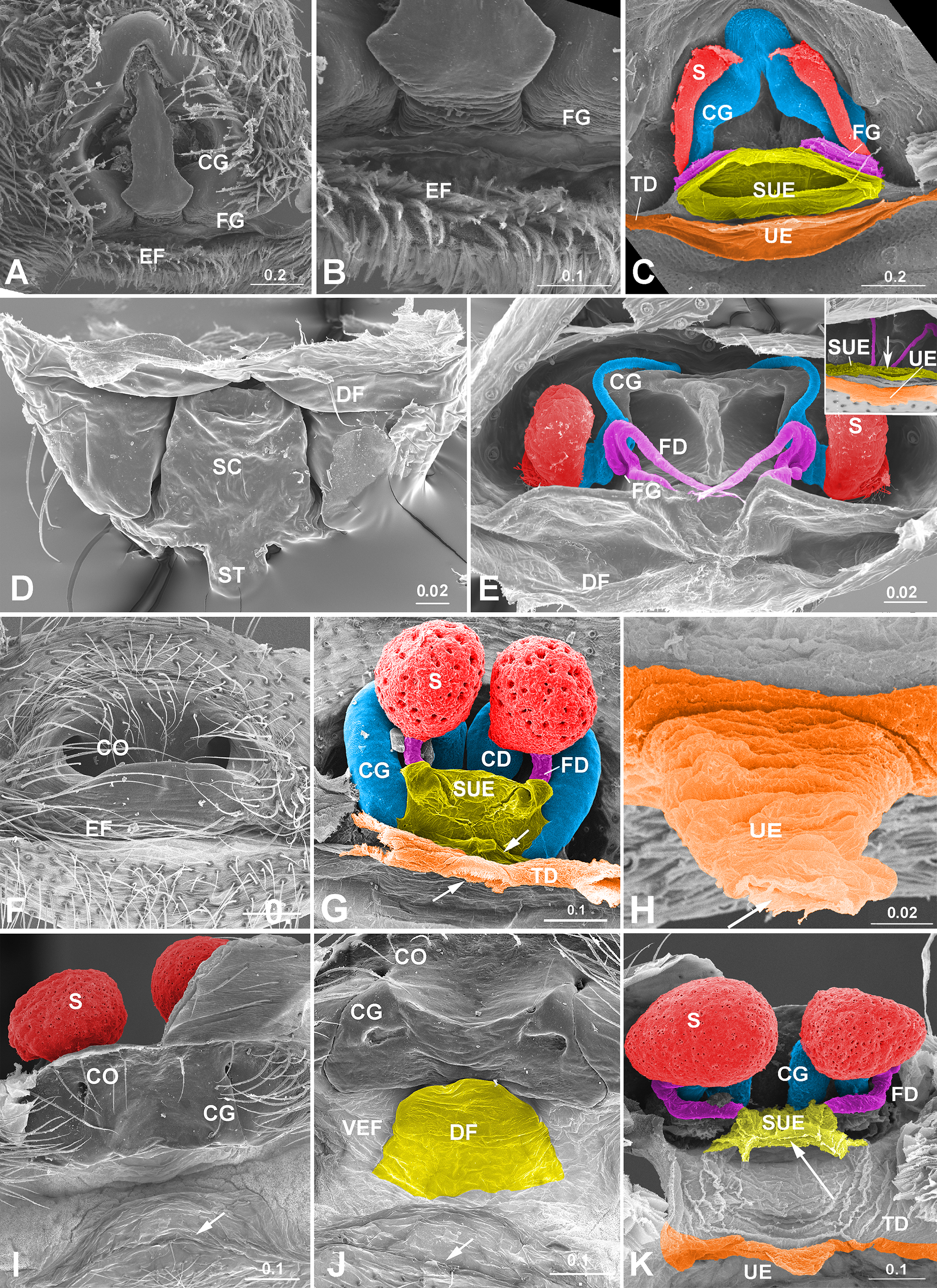

Supplement: S1 Fig — (A–C) Pardosa chionophila (Lycosidae), Type-I FG. (D–E) Agyneta sp. (Linyphiidae), Type-II FG+AFD, arrow to sperm-like granules in SUE. (F–H) Parasteatoda tepidariorum (Theridiidae), Type-III FD. (I–K) Nephila clavata (Araneidae), Type-IV PFD. Scale bars: mm. (TIF) [file pone.0218486.s007.tif]

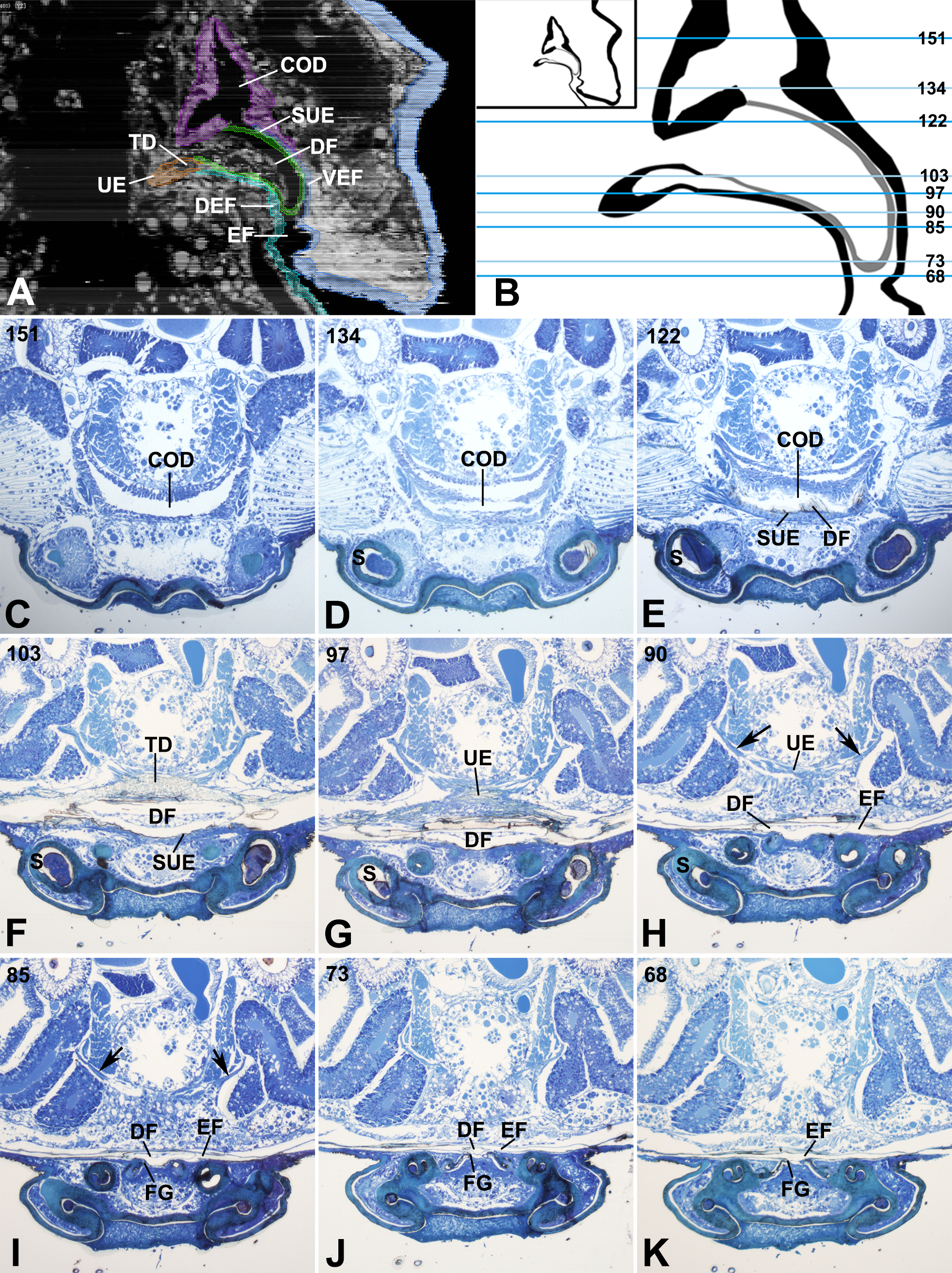

Supplement: S2 Fig — (A) Longitudinal section with structures lined in colors. (B) Line drawing of (A), square shows corresponding part in detail, lines indicate sections positions in (C-K), numbers refer to relevant numbers of slices. (C-E) Sections crossing COD. (F-I) Sections around UE internal end, arrows to muscles attached to sides of UE end. (J-K) Sections crossing EF and DF. COD, common oviduct; DEF, dorsal EF wall; DF, dorsal fold; EF, epigastric furrow; SUE, secondary uterus externus; TD, transversal duct; UE, uterus externus; VEF, ventral EF wall. (TIF) [file pone.0218486.s008.tif]

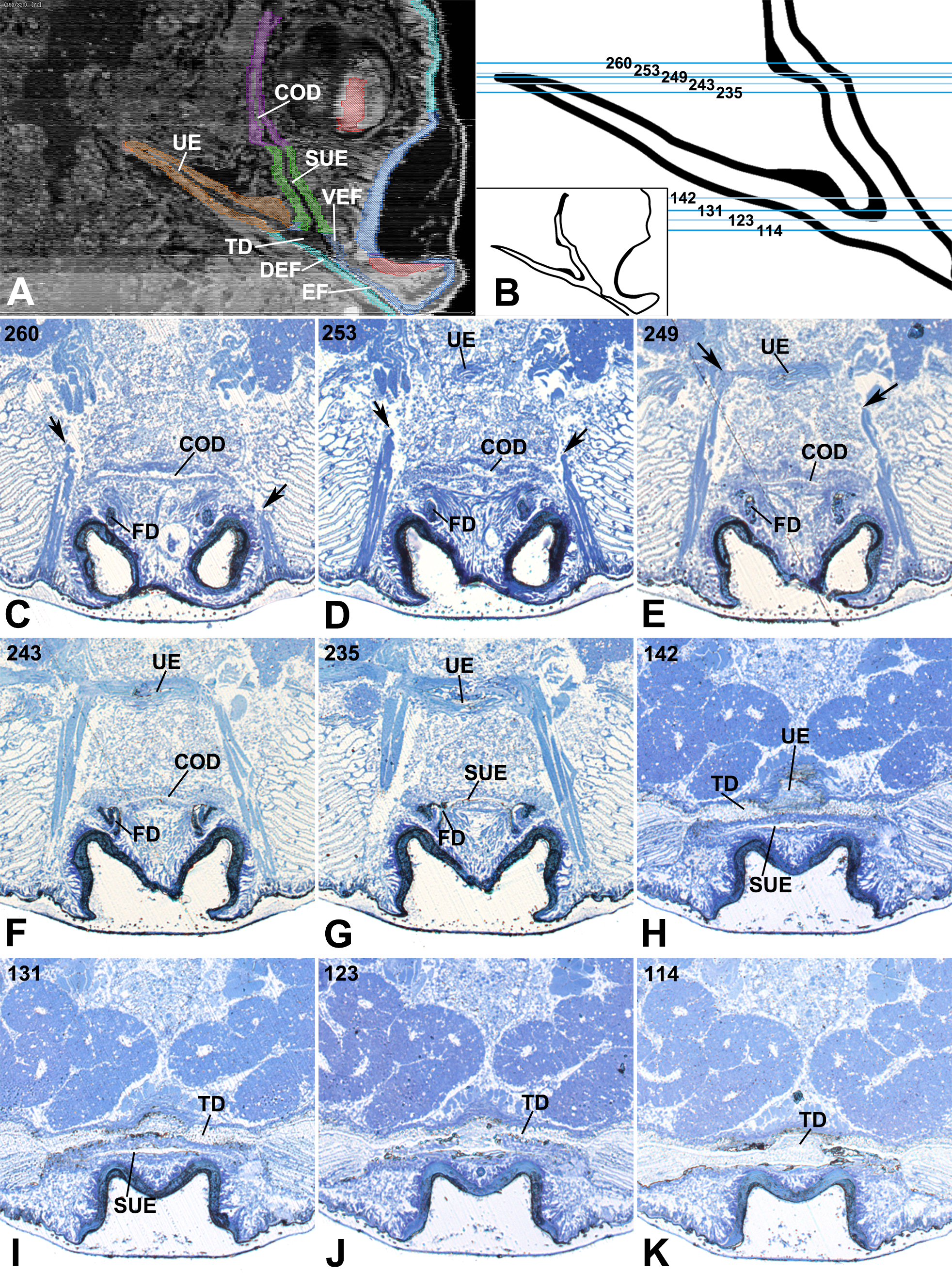

Supplement: S3 Fig — (A) Longitudinal section with structures lined in colors. (B) Line drawing of (A), square shows corresponding part in detail, lines indicate section positions in (C-K), numbers refer to relevant numbers of slices. (C-G) Sections around UE internal end, arrows to muscles attached to sides of UE end. (H-K) Sections around UE and SUE external openings. COD, common oviduct; DEF, dorsal EF wall; DF, dorsal fold; EF, epigastric furrow; SUE, secondary uterus externus; TD, transversal duct; UE, uterus externus; VEF, ventral EF wall. (TIF) [file pone.0218486.s009.tif]

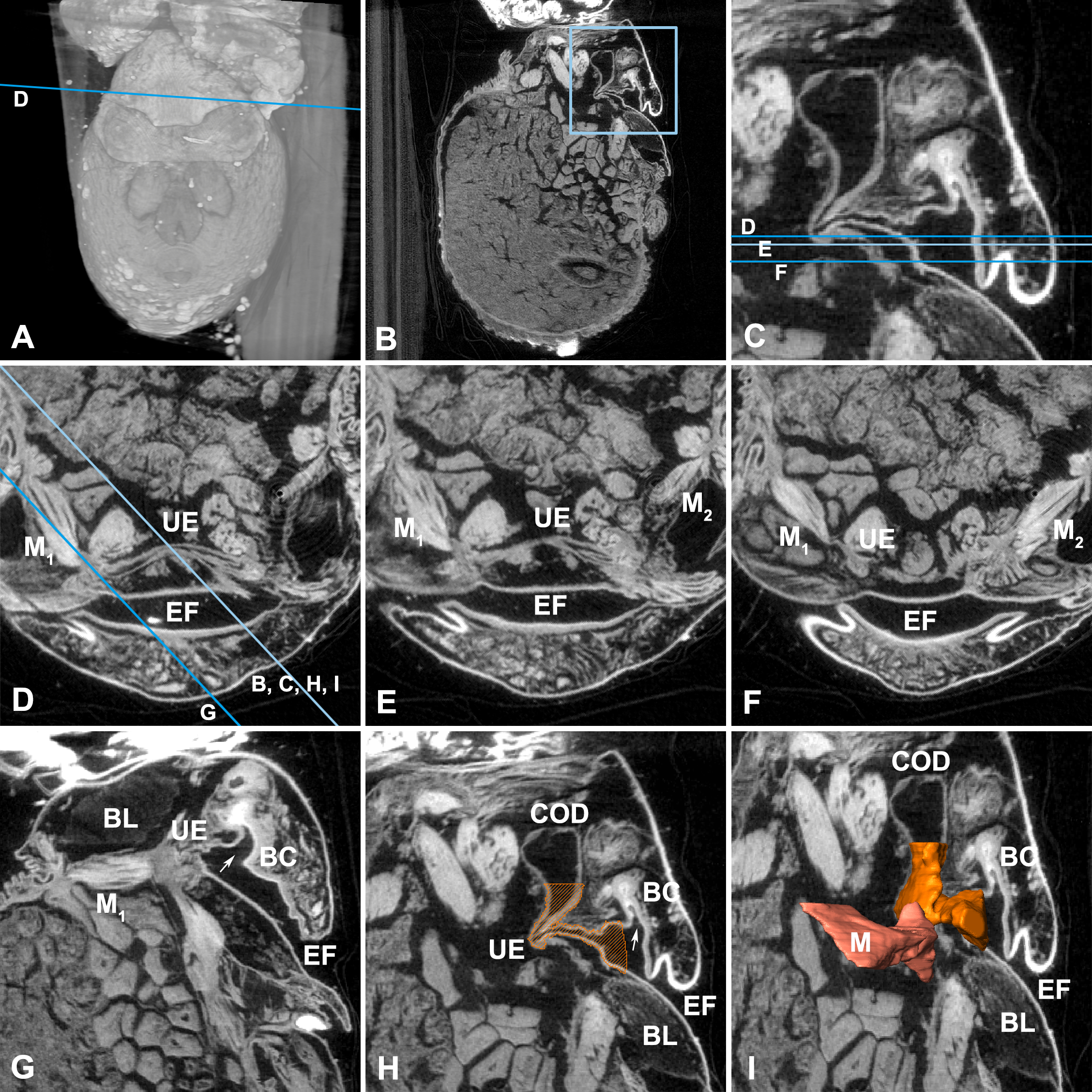

Supplement: S4 Fig — (A) Abdomen, ventral view, line shows section position of (D). (B) Longitudinal section with angle, section position shown in (D). (C) Detail of B, lines show section positions of (D-F). (D-F) Cross sections with angle. (D) Section crossing UE and left apodeme. (E) Section crossing turning point of UE. (F) Section crossing right apodeme and EF, note UE as a wide column with narrow and long chamber, and a pair of apodemes located at lateral sides of UE. (G-I) Longitudinal sections with angle, section positions shown in (D). (G) Section crossing left apodeme and EF. (H) Section crossing COD, UE and EF, note UE protruding internally from EF bottom, turning upwards to connect to COD (shadow). (I) Same section with reconstructed COD, UE, EF and right apodeme and muscles, BC opening to VEF (arrows). BC, bursa copulatrix; BL, book lung; COD, common oviduct; EF, epigastric furrow; M1, left bundle of muscles; M2, right bundle of muscles; S, spermatheca; UE, uterus externus; VEF, ventral EF wall. (TIF) [file pone.0218486.s010.tif]

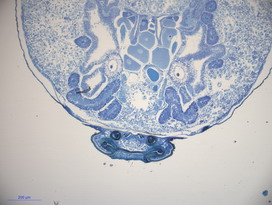

Supplement: S5 Fig — (ZIP) [file pone.0218486.s011.zip › T1570_237/T1570-0080_τ╝⌐σ░Åσñoσ░Å.jpg]

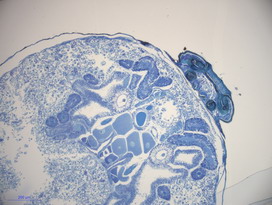

Supplement: S5 Fig — (ZIP) [file pone.0218486.s011.zip › T1570_237/T1570-0081_τ╝⌐σ░Åσñoσ░Å.jpg]

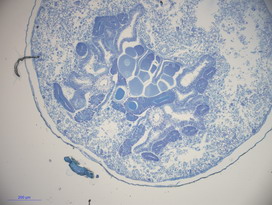

Supplement: S5 Fig — (ZIP) [file pone.0218486.s011.zip › T1570_237/T1570-0012_τ╝⌐σ░Åσñoσ░Å.jpg]

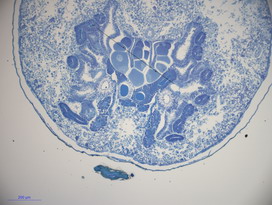

Supplement: S5 Fig — (ZIP) [file pone.0218486.s011.zip › T1570_237/T1570-0013_τ╝⌐σ░Åσñoσ░Å.jpg]

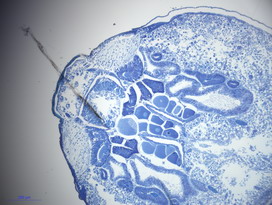

Supplement: S5 Fig — (ZIP) [file pone.0218486.s011.zip › T1570_237/T1570-0188_τ╝⌐σ░Åσñoσ░Å.jpg]

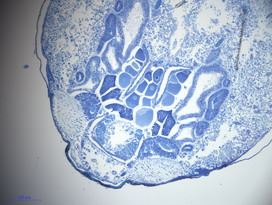

Supplement: S5 Fig — (ZIP) [file pone.0218486.s011.zip › T1570_237/T1570-0189_τ╝⌐σ░Åσñoσ░Å.jpg]

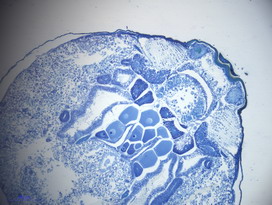

Supplement: S5 Fig — (ZIP) [file pone.0218486.s011.zip › T1570_237/T1570-0153_τ╝⌐σ░Åσñoσ░Å.jpg]

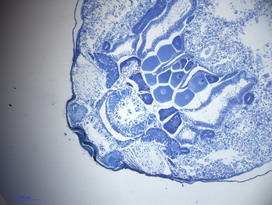

Supplement: S5 Fig — (ZIP) [file pone.0218486.s011.zip › T1570_237/T1570-0152_τ╝⌐σ░Åσñoσ░Å.jpg]

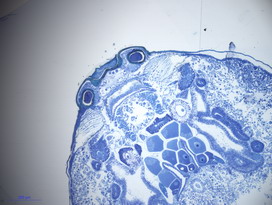

Supplement: S5 Fig — (ZIP) [file pone.0218486.s011.zip › T1570_237/T1570-0124_τ╝⌐σ░Åσñoσ░Å.jpg]

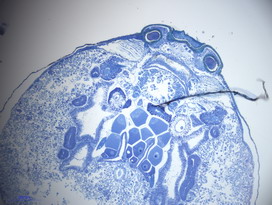

Supplement: S5 Fig — (ZIP) [file pone.0218486.s011.zip › T1570_237/T1570-0125_τ╝⌐σ░Åσñoσ░Å.jpg]

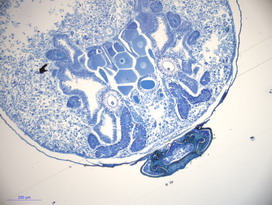

Supplement: S5 Fig — (ZIP) [file pone.0218486.s011.zip › T1570_237/T1570-0065_τ╝⌐σ░Åσñoσ░Å.jpg]

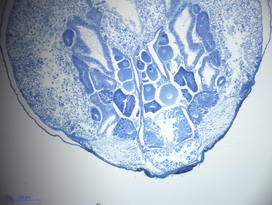

Supplement: S5 Fig — (ZIP) [file pone.0218486.s011.zip › T1570_237/T1570-0203_τ╝⌐σ░Åσñoσ░Å.jpg]

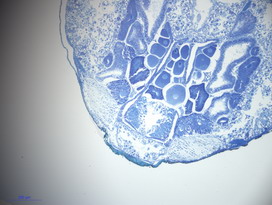

Supplement: S5 Fig — (ZIP) [file pone.0218486.s011.zip › T1570_237/T1570-0202_τ╝⌐σ░Åσñoσ░Å.jpg]

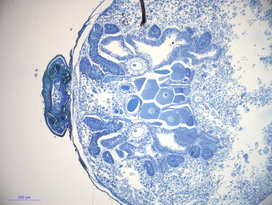

Supplement: S5 Fig — (ZIP) [file pone.0218486.s011.zip › T1570_237/T1570-0064_τ╝⌐σ░Åσñoσ░Å.jpg]

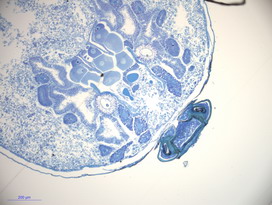

Supplement: S5 Fig — (ZIP) [file pone.0218486.s011.zip › T1570_237/T1570-0051_τ╝⌐σ░Åσñoσ░Å.jpg]

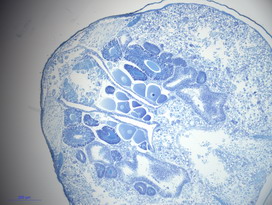

Supplement: S5 Fig — (ZIP) [file pone.0218486.s011.zip › T1570_237/T1570-0237_τ╝⌐σ░Åσñoσ░Å.jpg]

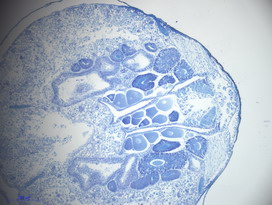

Supplement: S5 Fig — (ZIP) [file pone.0218486.s011.zip › T1570_237/T1570-0236_τ╝⌐σ░Åσñoσ░Å.jpg]

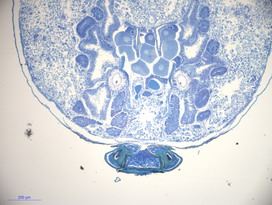

Supplement: S5 Fig — (ZIP) [file pone.0218486.s011.zip › T1570_237/T1570-0050_τ╝⌐σ░Åσñoσ░Å.jpg]

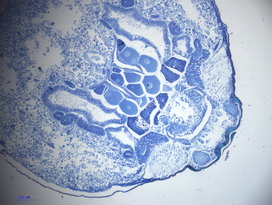

Supplement: S5 Fig — (ZIP) [file pone.0218486.s011.zip › T1570_237/T1570-0159_τ╝⌐σ░Åσñoσ░Å.jpg]

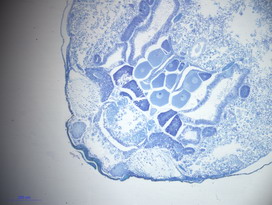

Supplement: S5 Fig — (ZIP) [file pone.0218486.s011.zip › T1570_237/T1570-0158_τ╝⌐σ░Åσñoσ░Å.jpg]

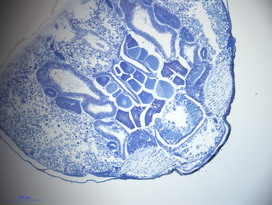

Supplement: S5 Fig — (ZIP) [file pone.0218486.s011.zip › T1570_237/T1570-0182_τ╝⌐σ░Åσñoσ░Å.jpg]

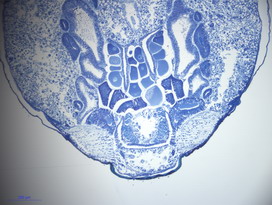

Supplement: S5 Fig — (ZIP) [file pone.0218486.s011.zip › T1570_237/T1570-0183_τ╝⌐σ░Åσñoσ░Å.jpg]

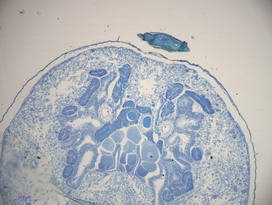

Supplement: S5 Fig — (ZIP) [file pone.0218486.s011.zip › T1570_237/T1570-0018_τ╝⌐σ░Åσñoσ░Å.jpg]

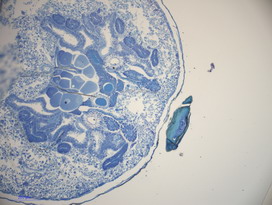

Supplement: S5 Fig — (ZIP) [file pone.0218486.s011.zip › T1570_237/T1570-0019_τ╝⌐σ░Åσñoσ░Å.jpg]

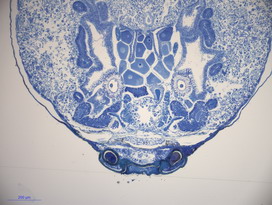

Supplement: S5 Fig — (ZIP) [file pone.0218486.s011.zip › T1570_237/T1570-0110_τ╝⌐σ░Åσñoσ░Å.jpg]

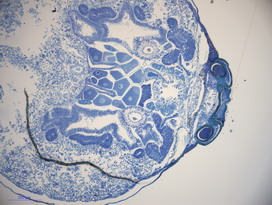

Supplement: S5 Fig — (ZIP) [file pone.0218486.s011.zip › T1570_237/T1570-0111_τ╝⌐σ░Åσñoσ░Å.jpg]

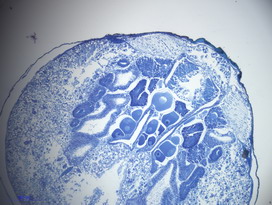

Supplement: S5 Fig — (ZIP) [file pone.0218486.s011.zip › T1570_237/T1570-0209_τ╝⌐σ░Åσñoσ░Å.jpg]

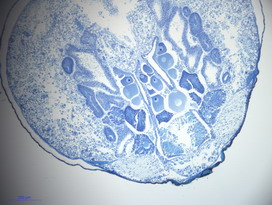

Supplement: S5 Fig — (ZIP) [file pone.0218486.s011.zip › T1570_237/T1570-0208_τ╝⌐σ░Åσñoσ░Å.jpg]

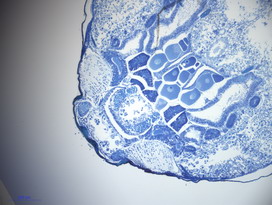

Supplement: S5 Fig — (ZIP) [file pone.0218486.s011.zip › T1570_237/T1570-0167_τ╝⌐σ░Åσñoσ░Å.jpg]

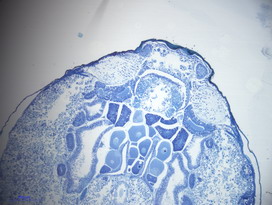

Supplement: S5 Fig — (ZIP) [file pone.0218486.s011.zip › T1570_237/T1570-0166_τ╝⌐σ░Åσñoσ░Å.jpg]

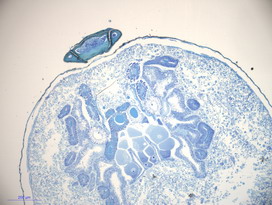

Supplement: S5 Fig — (ZIP) [file pone.0218486.s011.zip › T1570_237/T1570-0026_τ╝⌐σ░Åσñoσ░Å.jpg]

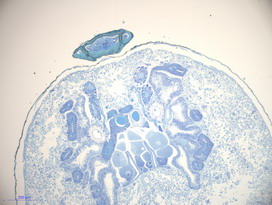

Supplement: S5 Fig — (ZIP) [file pone.0218486.s011.zip › T1570_237/T1570-0027_τ╝⌐σ░Åσñoσ░Å.jpg]

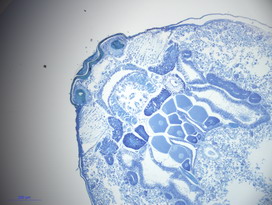

Supplement: S5 Fig — (ZIP) [file pone.0218486.s011.zip › T1570_237/T1570-0144_τ╝⌐σ░Åσñoσ░Å.jpg]

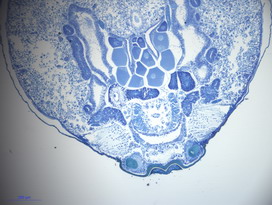

Supplement: S5 Fig — (ZIP) [file pone.0218486.s011.zip › T1570_237/T1570-0145_τ╝⌐σ░Åσñoσ░Å.jpg]

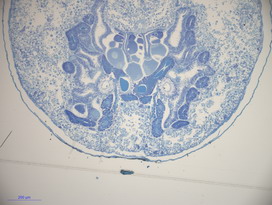

Supplement: S5 Fig — (ZIP) [file pone.0218486.s011.zip › T1570_237/T1570-0005_τ╝⌐σ░Åσñoσ░Å.jpg]

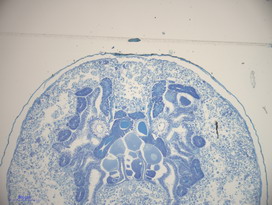

Supplement: S5 Fig — (ZIP) [file pone.0218486.s011.zip › T1570_237/T1570-0004_τ╝⌐σ░Åσñoσ░Å.jpg]

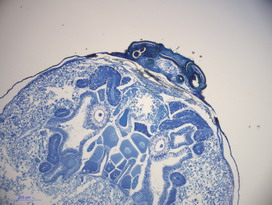

Supplement: S5 Fig — (ZIP) [file pone.0218486.s011.zip › T1570_237/T1570-0097_τ╝⌐σ░Åσñoσ░Å.jpg]

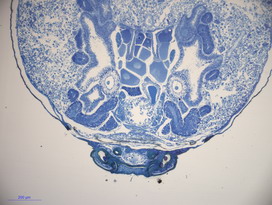

Supplement: S5 Fig — (ZIP) [file pone.0218486.s011.zip › T1570_237/T1570-0096_τ╝⌐σ░Åσñoσ░Å.jpg]

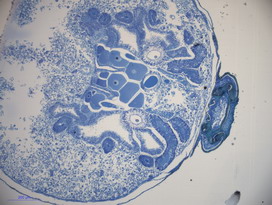

Supplement: S5 Fig — (ZIP) [file pone.0218486.s011.zip › T1570_237/T1570-0072_τ╝⌐σ░Åσñoσ░Å.jpg]

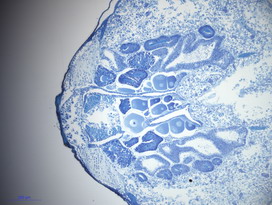

Supplement: S5 Fig — (ZIP) [file pone.0218486.s011.zip › T1570_237/T1570-0214_τ╝⌐σ░Åσñoσ░Å.jpg]

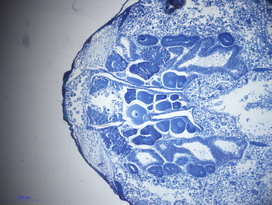

Supplement: S5 Fig — (ZIP) [file pone.0218486.s011.zip › T1570_237/T1570-0215_τ╝⌐σ░Åσñoσ░Å.jpg]

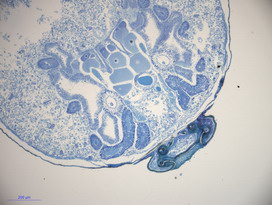

Supplement: S5 Fig — (ZIP) [file pone.0218486.s011.zip › T1570_237/T1570-0073_τ╝⌐σ░Åσñoσ░Å.jpg]

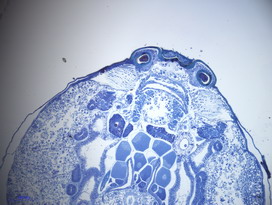

Supplement: S5 Fig — (ZIP) [file pone.0218486.s011.zip › T1570_237/T1570-0133_τ╝⌐σ░Åσñoσ░Å.jpg]

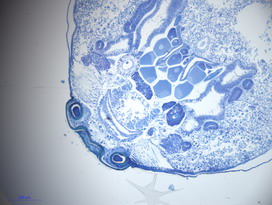

Supplement: S5 Fig — (ZIP) [file pone.0218486.s011.zip › T1570_237/T1570-0132_τ╝⌐σ░Åσñoσ░Å.jpg]

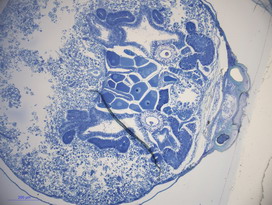

Supplement: S5 Fig — (ZIP) [file pone.0218486.s011.zip › T1570_237/T1570-0107_τ╝⌐σ░Åσñoσ░Å.jpg]

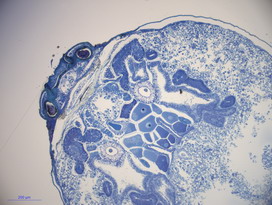

Supplement: S5 Fig — (ZIP) [file pone.0218486.s011.zip › T1570_237/T1570-0106_τ╝⌐σ░Åσñoσ░Å.jpg]

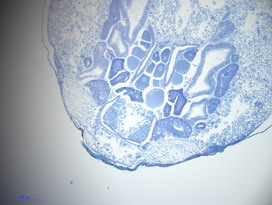

Supplement: S5 Fig — (ZIP) [file pone.0218486.s011.zip › T1570_237/T1570-0195_τ╝⌐σ░Åσñoσ░Å.jpg]

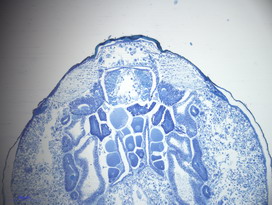

Supplement: S5 Fig — (ZIP) [file pone.0218486.s011.zip › T1570_237/T1570-0194_τ╝⌐σ░Åσñoσ░Å.jpg]

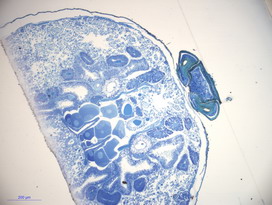

Supplement: S5 Fig — (ZIP) [file pone.0218486.s011.zip › T1570_237/T1570-0046_τ╝⌐σ░Åσñoσ░Å.jpg]

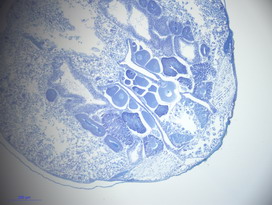

Supplement: S5 Fig — (ZIP) [file pone.0218486.s011.zip › T1570_237/T1570-0220_τ╝⌐σ░Åσñoσ░Å.jpg]

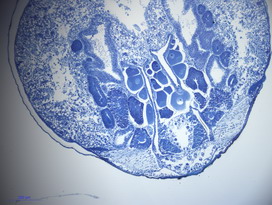

Supplement: S5 Fig — (ZIP) [file pone.0218486.s011.zip › T1570_237/T1570-0221_τ╝⌐σ░Åσñoσ░Å.jpg]

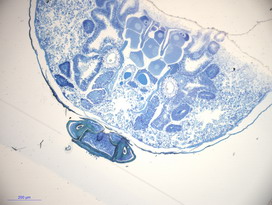

Supplement: S5 Fig — (ZIP) [file pone.0218486.s011.zip › T1570_237/T1570-0047_τ╝⌐σ░Åσñoσ░Å.jpg]

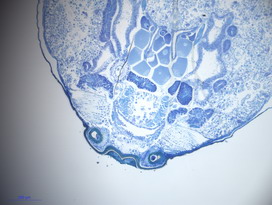

Supplement: S5 Fig — (ZIP) [file pone.0218486.s011.zip › T1570_237/T1570-0139_τ╝⌐σ░Åσñoσ░Å.jpg]

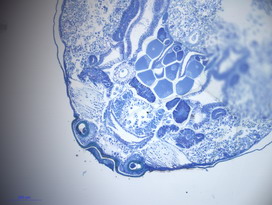

Supplement: S5 Fig — (ZIP) [file pone.0218486.s011.zip › T1570_237/T1570-0138_τ╝⌐σ░Åσñoσ░Å.jpg]

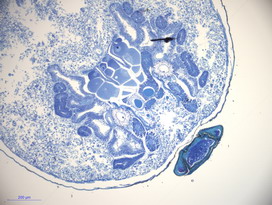

Supplement: S5 Fig — (ZIP) [file pone.0218486.s011.zip › T1570_237/T1570-0031_τ╝⌐σ░Åσñoσ░Å.jpg]

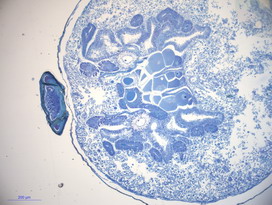

Supplement: S5 Fig — (ZIP) [file pone.0218486.s011.zip › T1570_237/T1570-0030_τ╝⌐σ░Åσñoσ░Å.jpg]

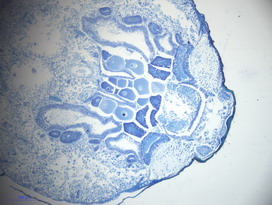

Supplement: S5 Fig — (ZIP) [file pone.0218486.s011.zip › T1570_237/T1570-0170_τ╝⌐σ░Åσñoσ░Å.jpg]

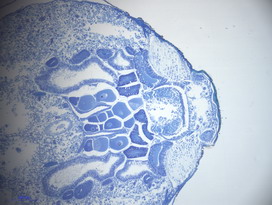

Supplement: S5 Fig — (ZIP) [file pone.0218486.s011.zip › T1570_237/T1570-0171_τ╝⌐σ░Åσñoσ░Å.jpg]

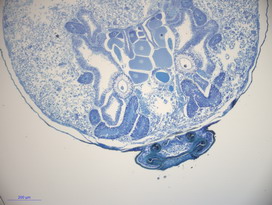

Supplement: S5 Fig — (ZIP) [file pone.0218486.s011.zip › T1570_237/T1570-0078_τ╝⌐σ░Åσñoσ░Å.jpg]

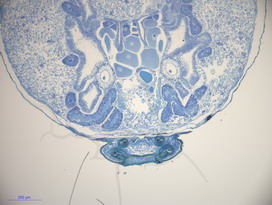

Supplement: S5 Fig — (ZIP) [file pone.0218486.s011.zip › T1570_237/T1570-0079_τ╝⌐σ░Åσñoσ░Å.jpg]

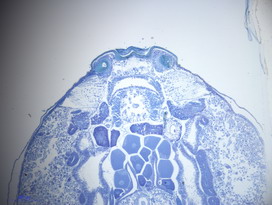

Supplement: S5 Fig — (ZIP) [file pone.0218486.s011.zip › T1570_237/T1570-0143_τ╝⌐σ░Åσñoσ░Å.jpg]

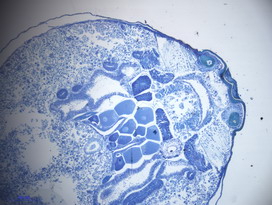

Supplement: S5 Fig — (ZIP) [file pone.0218486.s011.zip › T1570_237/T1570-0142_τ╝⌐σ░Åσñoσ░Å.jpg]

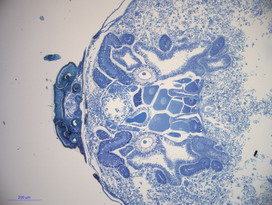

Supplement: S5 Fig — (ZIP) [file pone.0218486.s011.zip › T1570_237/T1570-0090_τ╝⌐σ░Åσñoσ░Å.jpg]

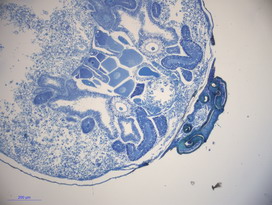

Supplement: S5 Fig — (ZIP) [file pone.0218486.s011.zip › T1570_237/T1570-0091_τ╝⌐σ░Åσñoσ░Å.jpg]

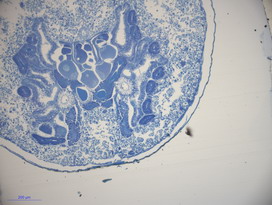

Supplement: S5 Fig — (ZIP) [file pone.0218486.s011.zip › T1570_237/T1570-0002_τ╝⌐σ░Åσñoσ░Å.jpg]

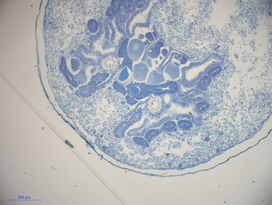

Supplement: S5 Fig — (ZIP) [file pone.0218486.s011.zip › T1570_237/T1570-0003_τ╝⌐σ░Åσñoσ░Å.jpg]

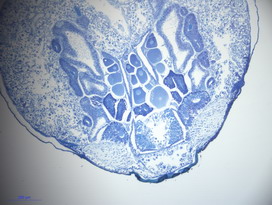

Supplement: S5 Fig — (ZIP) [file pone.0218486.s011.zip › T1570_237/T1570-0198_τ╝⌐σ░Åσñoσ░Å.jpg]

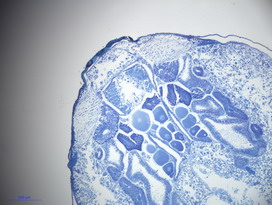

Supplement: S5 Fig — (ZIP) [file pone.0218486.s011.zip › T1570_237/T1570-0199_τ╝⌐σ░Åσñoσ░Å.jpg]

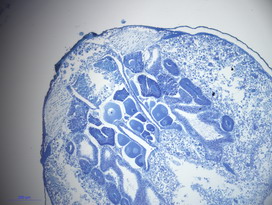

Supplement: S5 Fig — (ZIP) [file pone.0218486.s011.zip › T1570_237/T1570-0213_τ╝⌐σ░Åσñoσ░Å.jpg]

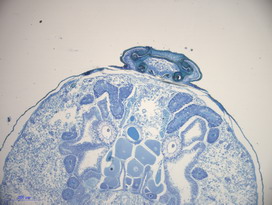

Supplement: S5 Fig — (ZIP) [file pone.0218486.s011.zip › T1570_237/T1570-0075_τ╝⌐σ░Åσñoσ░Å.jpg]

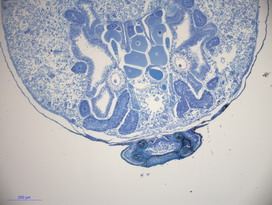

Supplement: S5 Fig — (ZIP) [file pone.0218486.s011.zip › T1570_237/T1570-0074_τ╝⌐σ░Åσñoσ░Å.jpg]

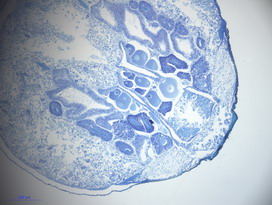

Supplement: S5 Fig — (ZIP) [file pone.0218486.s011.zip › T1570_237/T1570-0212_τ╝⌐σ░Åσñoσ░Å.jpg]

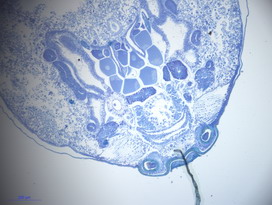

Supplement: S5 Fig — (ZIP) [file pone.0218486.s011.zip › T1570_237/T1570-0134_τ╝⌐σ░Åσñoσ░Å.jpg]

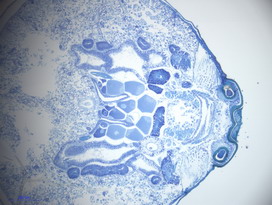

Supplement: S5 Fig — (ZIP) [file pone.0218486.s011.zip › T1570_237/T1570-0135_τ╝⌐σ░Åσñoσ░Å.jpg]

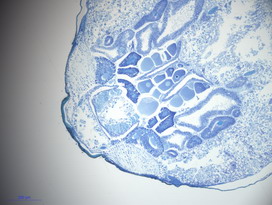

Supplement: S5 Fig — (ZIP) [file pone.0218486.s011.zip › T1570_237/T1570-0192_τ╝⌐σ░Åσñoσ░Å.jpg]

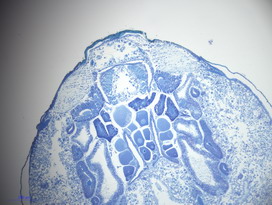

Supplement: S5 Fig — (ZIP) [file pone.0218486.s011.zip › T1570_237/T1570-0193_τ╝⌐σ░Åσñoσ░Å.jpg]

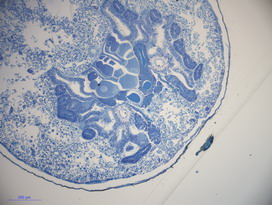

Supplement: S5 Fig — (ZIP) [file pone.0218486.s011.zip › T1570_237/T1570-0008_τ╝⌐σ░Åσñoσ░Å.jpg]

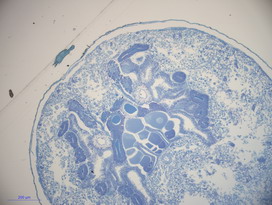

Supplement: S5 Fig — (ZIP) [file pone.0218486.s011.zip › T1570_237/T1570-0009_τ╝⌐σ░Åσñoσ░Å.jpg]

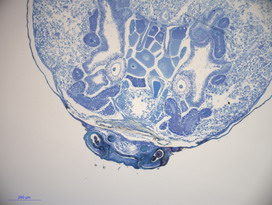

Supplement: S5 Fig — (ZIP) [file pone.0218486.s011.zip › T1570_237/T1570-0100_τ╝⌐σ░Åσñoσ░Å.jpg]

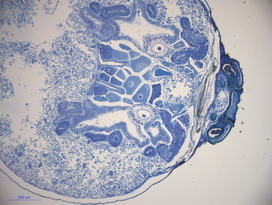

Supplement: S5 Fig — (ZIP) [file pone.0218486.s011.zip › T1570_237/T1570-0101_τ╝⌐σ░Åσñoσ░Å.jpg]

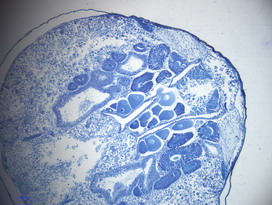

Supplement: S5 Fig — (ZIP) [file pone.0218486.s011.zip › T1570_237/T1570-0227_τ╝⌐σ░Åσñoσ░Å.jpg]

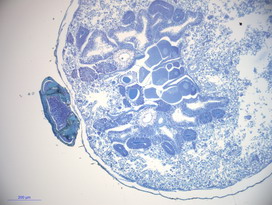

Supplement: S5 Fig — (ZIP) [file pone.0218486.s011.zip › T1570_237/T1570-0041_τ╝⌐σ░Åσñoσ░Å.jpg]

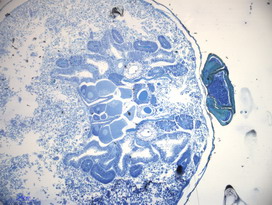

Supplement: S5 Fig — (ZIP) [file pone.0218486.s011.zip › T1570_237/T1570-0040_τ╝⌐σ░Åσñoσ░Å.jpg]

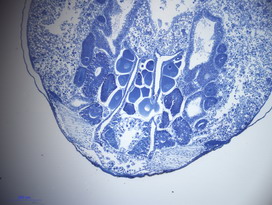

Supplement: S5 Fig — (ZIP) [file pone.0218486.s011.zip › T1570_237/T1570-0226_τ╝⌐σ░Åσñoσ░Å.jpg]

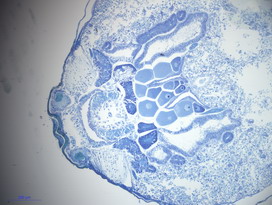

Supplement: S5 Fig — (ZIP) [file pone.0218486.s011.zip › T1570_237/T1570-0149_τ╝⌐σ░Åσñoσ░Å.jpg]

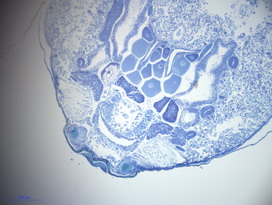

Supplement: S5 Fig — (ZIP) [file pone.0218486.s011.zip › T1570_237/T1570-0148_τ╝⌐σ░Åσñoσ░Å.jpg]

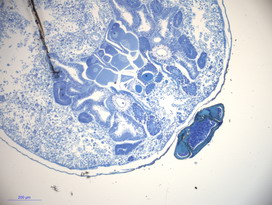

Supplement: S5 Fig — (ZIP) [file pone.0218486.s011.zip › T1570_237/T1570-0036_τ╝⌐σ░Åσñoσ░Å.jpg]

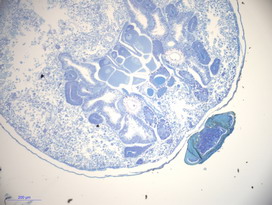

Supplement: S5 Fig — (ZIP) [file pone.0218486.s011.zip › T1570_237/T1570-0037_τ╝⌐σ░Åσñoσ░Å.jpg]

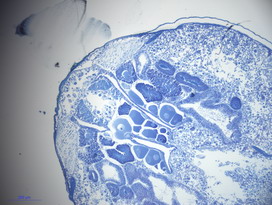

Supplement: S5 Fig — (ZIP) [file pone.0218486.s011.zip › T1570_237/T1570-0219_τ╝⌐σ░Åσñoσ░Å.jpg]

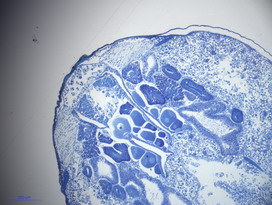

Supplement: S5 Fig — (ZIP) [file pone.0218486.s011.zip › T1570_237/T1570-0218_τ╝⌐σ░Åσñoσ░Å.jpg]

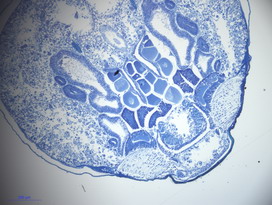

Supplement: S5 Fig — (ZIP) [file pone.0218486.s011.zip › T1570_237/T1570-0177_τ╝⌐σ░Åσñoσ░Å.jpg]

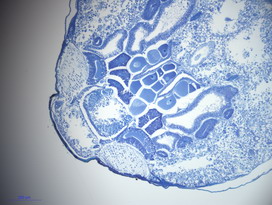

Supplement: S5 Fig — (ZIP) [file pone.0218486.s011.zip › T1570_237/T1570-0176_τ╝⌐σ░Åσñoσ░Å.jpg]

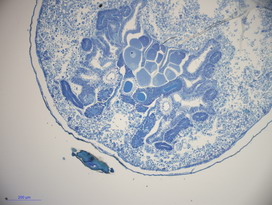

Supplement: S5 Fig — (ZIP) [file pone.0218486.s011.zip › T1570_237/T1570-0015_τ╝⌐σ░Åσñoσ░Å.jpg]

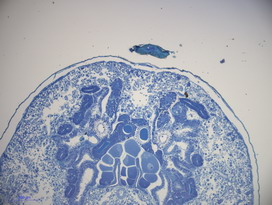

Supplement: S5 Fig — (ZIP) [file pone.0218486.s011.zip › T1570_237/T1570-0014_τ╝⌐σ░Åσñoσ░Å.jpg]

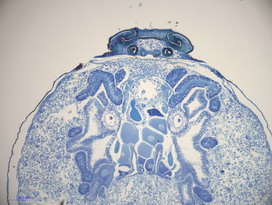

Supplement: S5 Fig — (ZIP) [file pone.0218486.s011.zip › T1570_237/T1570-0087_τ╝⌐σ░Åσñoσ░Å.jpg]

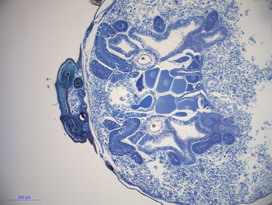

Supplement: S5 Fig — (ZIP) [file pone.0218486.s011.zip › T1570_237/T1570-0086_τ╝⌐σ░Åσñoσ░Å.jpg]
