# Supplementary figures and images for: Comparative morphology refines the conventional model of spider reproduction (part 3 of 5)
Source: PLoS One. 2019 Jul 5;14(7):e0218486. doi: 10.1371/journal.pone.0218486 (PMC6611574; doi:10.1371/journal.pone.0218486)

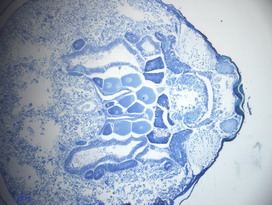

Supplement: S5 Fig — (ZIP) [file pone.0218486.s011.zip › T1570_237/T1570-0157_τ╝⌐σ░Åσñoσ░Å.jpg]

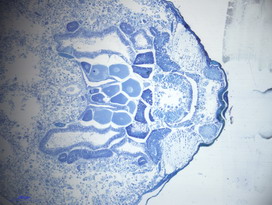

Supplement: S5 Fig — (ZIP) [file pone.0218486.s011.zip › T1570_237/T1570-0156_τ╝⌐σ░Åσñoσ░Å.jpg]

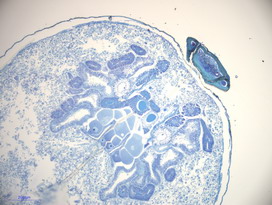

Supplement: S5 Fig — (ZIP) [file pone.0218486.s011.zip › T1570_237/T1570-0028_τ╝⌐σ░Åσñoσ░Å.jpg]

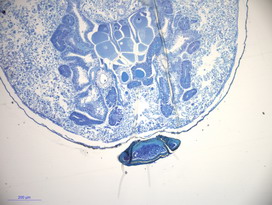

Supplement: S5 Fig — (ZIP) [file pone.0218486.s011.zip › T1570_237/T1570-0029_τ╝⌐σ░Åσñoσ░Å.jpg]

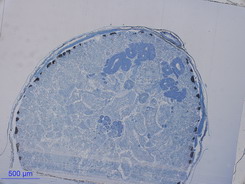

Supplement: S6 Fig — (ZIP) [file pone.0218486.s012.zip › T6055_469/T6085-0043_τ╝⌐σ░Åσñoσ░Å.jpg]

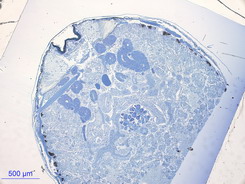

Supplement: S6 Fig — (ZIP) [file pone.0218486.s012.zip › T6055_469/T6085-0225_τ╝⌐σ░Åσñoσ░Å.jpg]

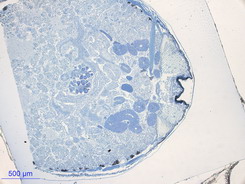

Supplement: S6 Fig — (ZIP) [file pone.0218486.s012.zip › T6055_469/T6085-0224_τ╝⌐σ░Åσñoσ░Å.jpg]

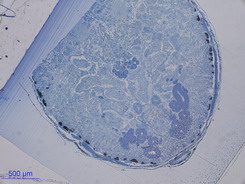

Supplement: S6 Fig — (ZIP) [file pone.0218486.s012.zip › T6055_469/T6085-0042_τ╝⌐σ░Åσñoσ░Å.jpg]

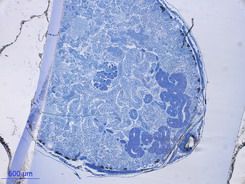

Supplement: S6 Fig — (ZIP) [file pone.0218486.s012.zip › T6055_469/T6085-0098_τ╝⌐σ░Åσñoσ░Å.jpg]

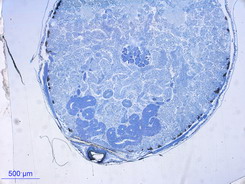

Supplement: S6 Fig — (ZIP) [file pone.0218486.s012.zip › T6055_469/T6085-0099_τ╝⌐σ░Åσñoσ░Å.jpg]

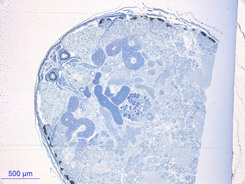

Supplement: S6 Fig — (ZIP) [file pone.0218486.s012.zip › T6055_469/T6085-0456_τ╝⌐σ░Åσñoσ░Å.jpg]

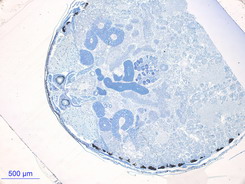

Supplement: S6 Fig — (ZIP) [file pone.0218486.s012.zip › T6055_469/T6085-0457_τ╝⌐σ░Åσñoσ░Å.jpg]

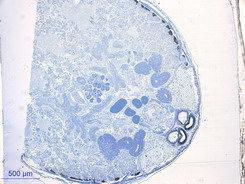

Supplement: S6 Fig — (ZIP) [file pone.0218486.s012.zip › T6055_469/T6085-0364_τ╝⌐σ░Åσñoσ░Å.jpg]

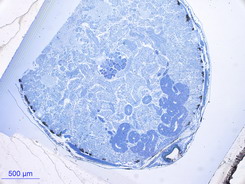

Supplement: S6 Fig — (ZIP) [file pone.0218486.s012.zip › T6055_469/T6085-0102_τ╝⌐σ░Åσñoσ░Å.jpg]

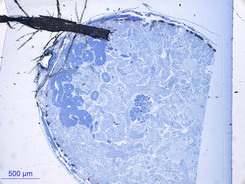

Supplement: S6 Fig — (ZIP) [file pone.0218486.s012.zip › T6055_469/T6085-0103_τ╝⌐σ░Åσñoσ░Å.jpg]

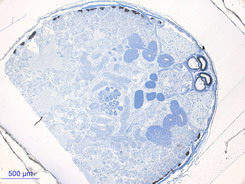

Supplement: S6 Fig — (ZIP) [file pone.0218486.s012.zip › T6055_469/T6085-0365_τ╝⌐σ░Åσñoσ░Å.jpg]

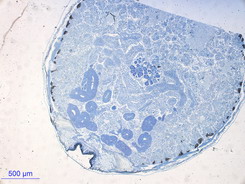

Supplement: S6 Fig — (ZIP) [file pone.0218486.s012.zip › T6055_469/T6085-0190_τ╝⌐σ░Åσñoσ░Å.jpg]

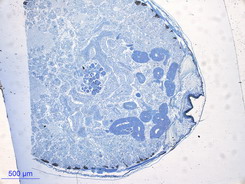

Supplement: S6 Fig — (ZIP) [file pone.0218486.s012.zip › T6055_469/T6085-0191_τ╝⌐σ░Åσñoσ░Å.jpg]

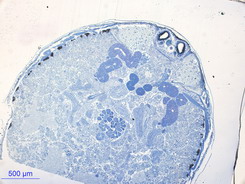

Supplement: S6 Fig — (ZIP) [file pone.0218486.s012.zip › T6055_469/T6085-0289_τ╝⌐σ░Åσñoσ░Å.jpg]

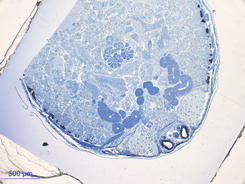

Supplement: S6 Fig — (ZIP) [file pone.0218486.s012.zip › T6055_469/T6085-0288_τ╝⌐σ░Åσñoσ░Å.jpg]

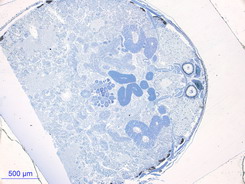

Supplement: S6 Fig — (ZIP) [file pone.0218486.s012.zip › T6055_469/T6085-0421_τ╝⌐σ░Åσñoσ░Å.jpg]

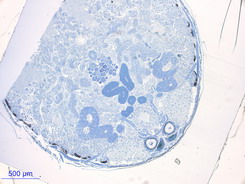

Supplement: S6 Fig — (ZIP) [file pone.0218486.s012.zip › T6055_469/T6085-0420_τ╝⌐σ░Åσñoσ░Å.jpg]

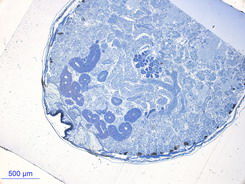

Supplement: S6 Fig — (ZIP) [file pone.0218486.s012.zip › T6055_469/T6085-0175_τ╝⌐σ░Åσñoσ░Å.jpg]

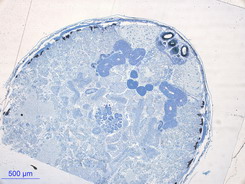

Supplement: S6 Fig — (ZIP) [file pone.0218486.s012.zip › T6055_469/T6085-0313_τ╝⌐σ░Åσñoσ░Å.jpg]

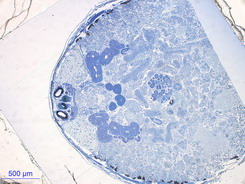

Supplement: S6 Fig — (ZIP) [file pone.0218486.s012.zip › T6055_469/T6085-0312_τ╝⌐σ░Åσñoσ░Å.jpg]

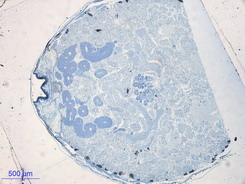

Supplement: S6 Fig — (ZIP) [file pone.0218486.s012.zip › T6055_469/T6085-0174_τ╝⌐σ░Åσñoσ░Å.jpg]

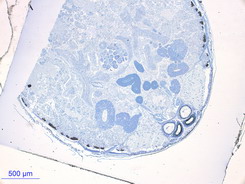

Supplement: S6 Fig — (ZIP) [file pone.0218486.s012.zip › T6055_469/T6085-0381_τ╝⌐σ░Åσñoσ░Å.jpg]

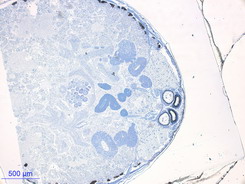

Supplement: S6 Fig — (ZIP) [file pone.0218486.s012.zip › T6055_469/T6085-0380_τ╝⌐σ░Åσñoσ░Å.jpg]

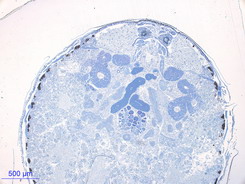

Supplement: S6 Fig — (ZIP) [file pone.0218486.s012.zip › T6055_469/T6085-0468_τ╝⌐σ░Åσñoσ░Å.jpg]

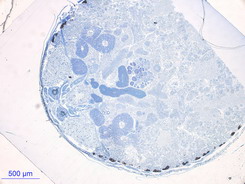

Supplement: S6 Fig — (ZIP) [file pone.0218486.s012.zip › T6055_469/T6085-0469_τ╝⌐σ░Åσñoσ░Å.jpg]

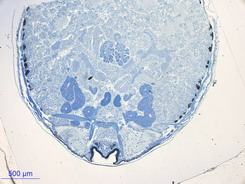

Supplement: S6 Fig — (ZIP) [file pone.0218486.s012.zip › T6055_469/T6085-0252_τ╝⌐σ░Åσñoσ░Å.jpg]

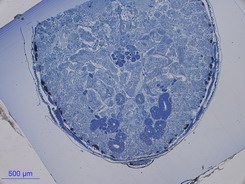

Supplement: S6 Fig — (ZIP) [file pone.0218486.s012.zip › T6055_469/T6085-0034_τ╝⌐σ░Åσñoσ░Å.jpg]

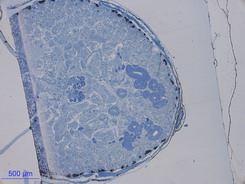

Supplement: S6 Fig — (ZIP) [file pone.0218486.s012.zip › T6055_469/T6085-0035_τ╝⌐σ░Åσñoσ░Å.jpg]

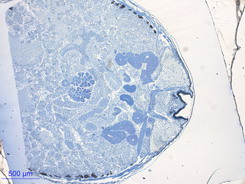

Supplement: S6 Fig — (ZIP) [file pone.0218486.s012.zip › T6055_469/T6085-0253_τ╝⌐σ░Åσñoσ░Å.jpg]

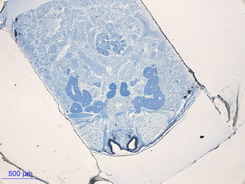

Supplement: S6 Fig — (ZIP) [file pone.0218486.s012.zip › T6055_469/T6085-0266_τ╝⌐σ░Åσñoσ░Å.jpg]

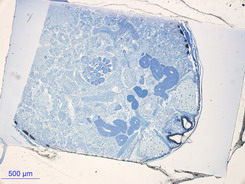

Supplement: S6 Fig — (ZIP) [file pone.0218486.s012.zip › T6055_469/T6085-0267_τ╝⌐σ░Åσñoσ░Å.jpg]

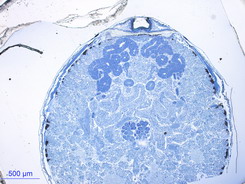

Supplement: S6 Fig — (ZIP) [file pone.0218486.s012.zip › T6055_469/T6085-0108_τ╝⌐σ░Åσñoσ░Å.jpg]

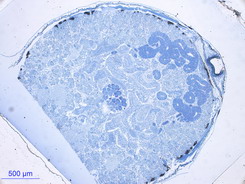

Supplement: S6 Fig — (ZIP) [file pone.0218486.s012.zip › T6055_469/T6085-0109_τ╝⌐σ░Åσñoσ░Å.jpg]

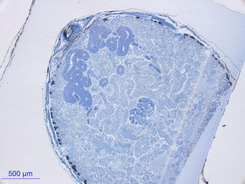

Supplement: S6 Fig — (ZIP) [file pone.0218486.s012.zip › T6055_469/T6085-0092_τ╝⌐σ░Åσñoσ░Å.jpg]

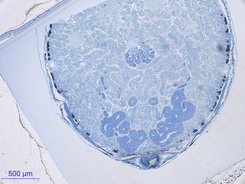

Supplement: S6 Fig — (ZIP) [file pone.0218486.s012.zip › T6055_469/T6085-0093_τ╝⌐σ░Åσñoσ░Å.jpg]

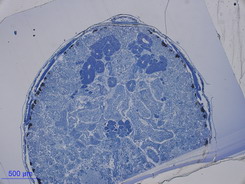

Supplement: S6 Fig — (ZIP) [file pone.0218486.s012.zip › T6055_469/T6085-0049_τ╝⌐σ░Åσñoσ░Å.jpg]

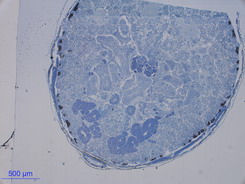

Supplement: S6 Fig — (ZIP) [file pone.0218486.s012.zip › T6055_469/T6085-0048_τ╝⌐σ░Åσñoσ░Å.jpg]

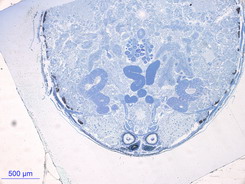

Supplement: S6 Fig — (ZIP) [file pone.0218486.s012.zip › T6055_469/T6085-0415_τ╝⌐σ░Åσñoσ░Å.jpg]

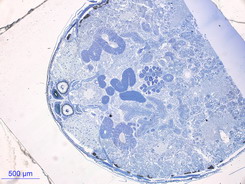

Supplement: S6 Fig — (ZIP) [file pone.0218486.s012.zip › T6055_469/T6085-0414_τ╝⌐σ░Åσñoσ░Å.jpg]

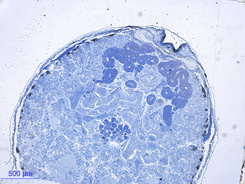

Supplement: S6 Fig — (ZIP) [file pone.0218486.s012.zip › T6055_469/T6085-0141_τ╝⌐σ░Åσñoσ░Å.jpg]

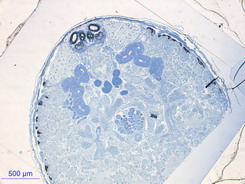

Supplement: S6 Fig — (ZIP) [file pone.0218486.s012.zip › T6055_469/T6085-0327_τ╝⌐σ░Åσñoσ░Å.jpg]

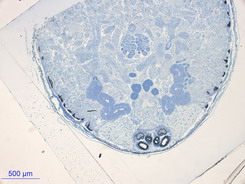

Supplement: S6 Fig — (ZIP) [file pone.0218486.s012.zip › T6055_469/T6085-0326_τ╝⌐σ░Åσñoσ░Å.jpg]

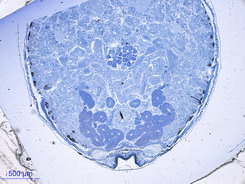

Supplement: S6 Fig — (ZIP) [file pone.0218486.s012.zip › T6055_469/T6085-0140_τ╝⌐σ░Åσñoσ░Å.jpg]

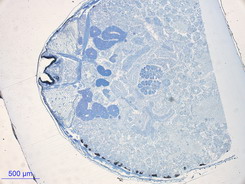

Supplement: S6 Fig — (ZIP) [file pone.0218486.s012.zip › T6055_469/T6085-0258_τ╝⌐σ░Åσñoσ░Å.jpg]

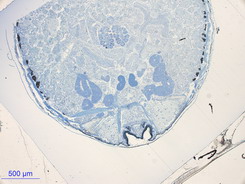

Supplement: S6 Fig — (ZIP) [file pone.0218486.s012.zip › T6055_469/T6085-0259_τ╝⌐σ░Åσñoσ░Å.jpg]

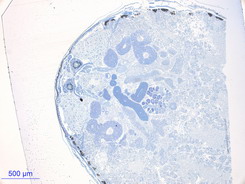

Supplement: S6 Fig — (ZIP) [file pone.0218486.s012.zip › T6055_469/T6085-0462_τ╝⌐σ░Åσñoσ░Å.jpg]

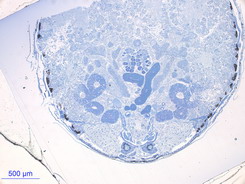

Supplement: S6 Fig — (ZIP) [file pone.0218486.s012.zip › T6055_469/T6085-0463_τ╝⌐σ░Åσñoσ░Å.jpg]

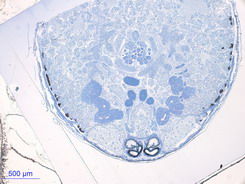

Supplement: S6 Fig — (ZIP) [file pone.0218486.s012.zip › T6055_469/T6085-0350_τ╝⌐σ░Åσñoσ░Å.jpg]

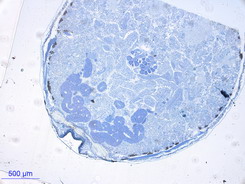

Supplement: S6 Fig — (ZIP) [file pone.0218486.s012.zip › T6055_469/T6085-0136_τ╝⌐σ░Åσñoσ░Å.jpg]

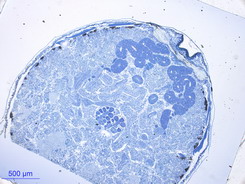

Supplement: S6 Fig — (ZIP) [file pone.0218486.s012.zip › T6055_469/T6085-0137_τ╝⌐σ░Åσñoσ░Å.jpg]

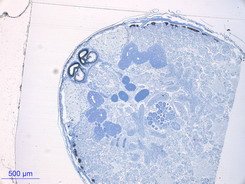

Supplement: S6 Fig — (ZIP) [file pone.0218486.s012.zip › T6055_469/T6085-0351_τ╝⌐σ░Åσñoσ░Å.jpg]

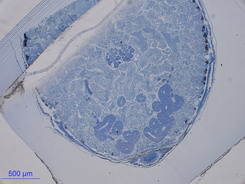

Supplement: S6 Fig — (ZIP) [file pone.0218486.s012.zip › T6055_469/T6085-0077_τ╝⌐σ░Åσñoσ░Å.jpg]

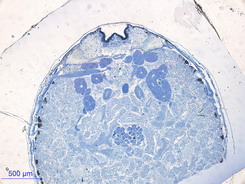

Supplement: S6 Fig — (ZIP) [file pone.0218486.s012.zip › T6055_469/T6085-0211_τ╝⌐σ░Åσñoσ░Å.jpg]

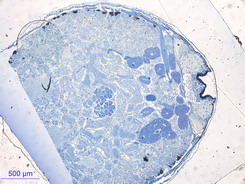

Supplement: S6 Fig — (ZIP) [file pone.0218486.s012.zip › T6055_469/T6085-0210_τ╝⌐σ░Åσñoσ░Å.jpg]

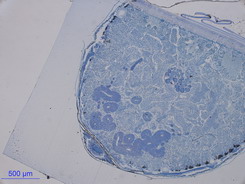

Supplement: S6 Fig — (ZIP) [file pone.0218486.s012.zip › T6055_469/T6085-0076_τ╝⌐σ░Åσñoσ░Å.jpg]

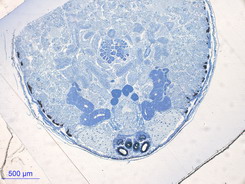

Supplement: S6 Fig — (ZIP) [file pone.0218486.s012.zip › T6055_469/T6085-0319_τ╝⌐σ░Åσñoσ░Å.jpg]

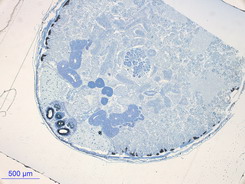

Supplement: S6 Fig — (ZIP) [file pone.0218486.s012.zip › T6055_469/T6085-0318_τ╝⌐σ░Åσñoσ░Å.jpg]

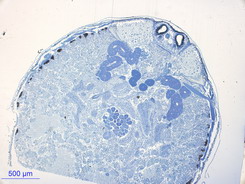

Supplement: S6 Fig — (ZIP) [file pone.0218486.s012.zip › T6055_469/T6085-0283_τ╝⌐σ░Åσñoσ░Å.jpg]

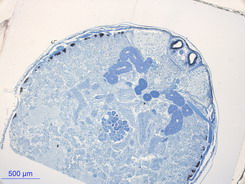

Supplement: S6 Fig — (ZIP) [file pone.0218486.s012.zip › T6055_469/T6085-0282_τ╝⌐σ░Åσñoσ░Å.jpg]

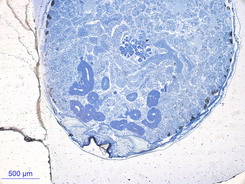

Supplement: S6 Fig — (ZIP) [file pone.0218486.s012.zip › T6055_469/T6085-0187_τ╝⌐σ░Åσñoσ░Å.jpg]

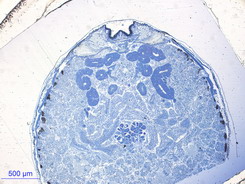

Supplement: S6 Fig — (ZIP) [file pone.0218486.s012.zip › T6055_469/T6085-0186_τ╝⌐σ░Åσñoσ░Å.jpg]

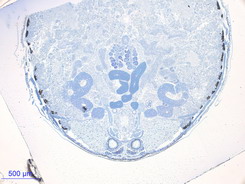

Supplement: S6 Fig — (ZIP) [file pone.0218486.s012.zip › T6055_469/T6085-0441_τ╝⌐σ░Åσñoσ░Å.jpg]

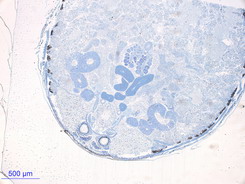

Supplement: S6 Fig — (ZIP) [file pone.0218486.s012.zip › T6055_469/T6085-0440_τ╝⌐σ░Åσñoσ░Å.jpg]

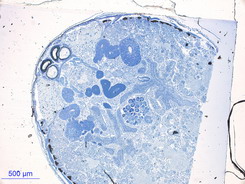

Supplement: S6 Fig — (ZIP) [file pone.0218486.s012.zip › T6055_469/T6085-0373_τ╝⌐σ░Åσñoσ░Å.jpg]

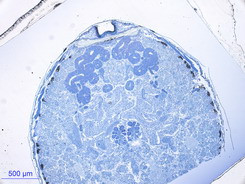

Supplement: S6 Fig — (ZIP) [file pone.0218486.s012.zip › T6055_469/T6085-0115_τ╝⌐σ░Åσñoσ░Å.jpg]

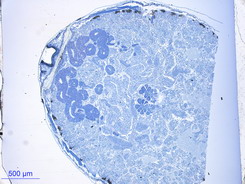

Supplement: S6 Fig — (ZIP) [file pone.0218486.s012.zip › T6055_469/T6085-0114_τ╝⌐σ░Åσñoσ░Å.jpg]

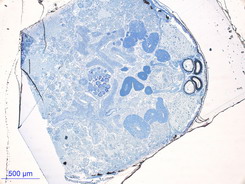

Supplement: S6 Fig — (ZIP) [file pone.0218486.s012.zip › T6055_469/T6085-0372_τ╝⌐σ░Åσñoσ░Å.jpg]

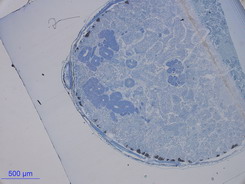

Supplement: S6 Fig — (ZIP) [file pone.0218486.s012.zip › T6055_469/T6085-0054_τ╝⌐σ░Åσñoσ░Å.jpg]

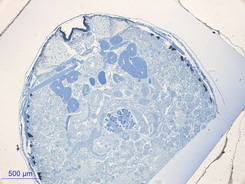

Supplement: S6 Fig — (ZIP) [file pone.0218486.s012.zip › T6055_469/T6085-0232_τ╝⌐σ░Åσñoσ░Å.jpg]

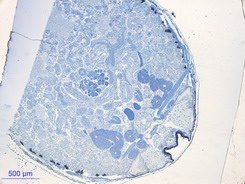

Supplement: S6 Fig — (ZIP) [file pone.0218486.s012.zip › T6055_469/T6085-0233_τ╝⌐σ░Åσñoσ░Å.jpg]

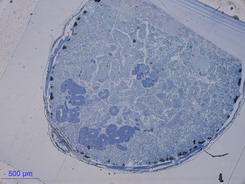

Supplement: S6 Fig — (ZIP) [file pone.0218486.s012.zip › T6055_469/T6085-0055_τ╝⌐σ░Åσñoσ░Å.jpg]

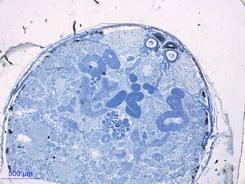

Supplement: S6 Fig — (ZIP) [file pone.0218486.s012.zip › T6055_469/T6085-0408_τ╝⌐σ░Åσñoσ░Å.jpg]

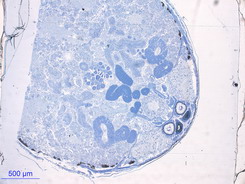

Supplement: S6 Fig — (ZIP) [file pone.0218486.s012.zip › T6055_469/T6085-0409_τ╝⌐σ░Åσñoσ░Å.jpg]

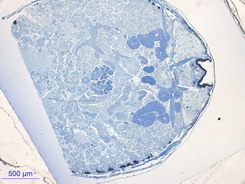

Supplement: S6 Fig — (ZIP) [file pone.0218486.s012.zip › T6055_469/T6085-0245_τ╝⌐σ░Åσñoσ░Å.jpg]

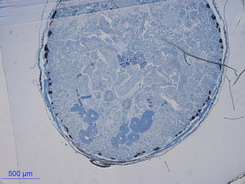

Supplement: S6 Fig — (ZIP) [file pone.0218486.s012.zip › T6055_469/T6085-0023_τ╝⌐σ░Åσñoσ░Å.jpg]

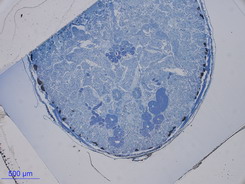

Supplement: S6 Fig — (ZIP) [file pone.0218486.s012.zip › T6055_469/T6085-0022_τ╝⌐σ░Åσñoσ░Å.jpg]

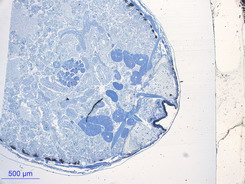

Supplement: S6 Fig — (ZIP) [file pone.0218486.s012.zip › T6055_469/T6085-0244_τ╝⌐σ░Åσñoσ░Å.jpg]

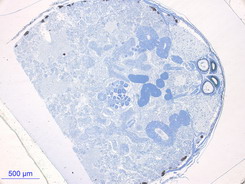

Supplement: S6 Fig — (ZIP) [file pone.0218486.s012.zip › T6055_469/T6085-0396_τ╝⌐σ░Åσñoσ░Å.jpg]

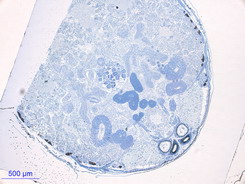

Supplement: S6 Fig — (ZIP) [file pone.0218486.s012.zip › T6055_469/T6085-0397_τ╝⌐σ░Åσñoσ░Å.jpg]

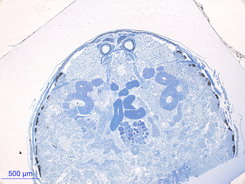

Supplement: S6 Fig — (ZIP) [file pone.0218486.s012.zip › T6055_469/T6085-0436_τ╝⌐σ░Åσñoσ░Å.jpg]

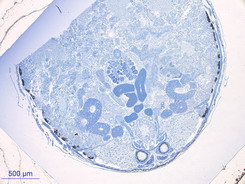

Supplement: S6 Fig — (ZIP) [file pone.0218486.s012.zip › T6055_469/T6085-0437_τ╝⌐σ░Åσñoσ░Å.jpg]

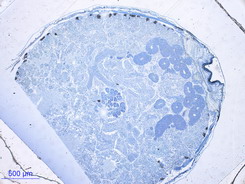

Supplement: S6 Fig — (ZIP) [file pone.0218486.s012.zip › T6055_469/T6085-0162_τ╝⌐σ░Åσñoσ░Å.jpg]

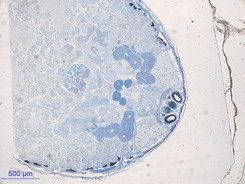

Supplement: S6 Fig — (ZIP) [file pone.0218486.s012.zip › T6055_469/T6085-0304_τ╝⌐σ░Åσñoσ░Å.jpg]

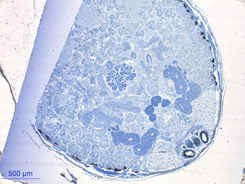

Supplement: S6 Fig — (ZIP) [file pone.0218486.s012.zip › T6055_469/T6085-0305_τ╝⌐σ░Åσñoσ░Å.jpg]

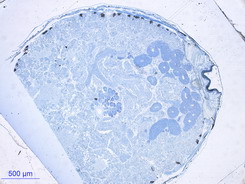

Supplement: S6 Fig — (ZIP) [file pone.0218486.s012.zip › T6055_469/T6085-0163_τ╝⌐σ░Åσñoσ░Å.jpg]

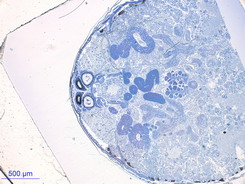

Supplement: S6 Fig — (ZIP) [file pone.0218486.s012.zip › T6055_469/T6085-0402_τ╝⌐σ░Åσñoσ░Å.jpg]

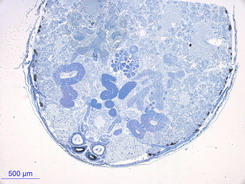

Supplement: S6 Fig — (ZIP) [file pone.0218486.s012.zip › T6055_469/T6085-0403_τ╝⌐σ░Åσñoσ░Å.jpg]

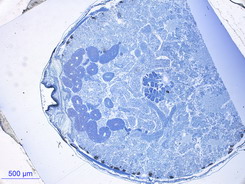

Supplement: S6 Fig — (ZIP) [file pone.0218486.s012.zip › T6055_469/T6085-0156_τ╝⌐σ░Åσñoσ░Å.jpg]

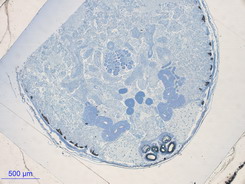

Supplement: S6 Fig — (ZIP) [file pone.0218486.s012.zip › T6055_469/T6085-0330_τ╝⌐σ░Åσñoσ░Å.jpg]

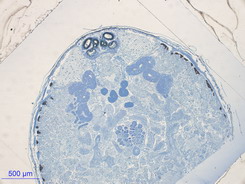

Supplement: S6 Fig — (ZIP) [file pone.0218486.s012.zip › T6055_469/T6085-0331_τ╝⌐σ░Åσñoσ░Å.jpg]

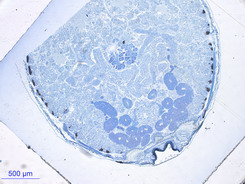

Supplement: S6 Fig — (ZIP) [file pone.0218486.s012.zip › T6055_469/T6085-0157_τ╝⌐σ░Åσñoσ░Å.jpg]

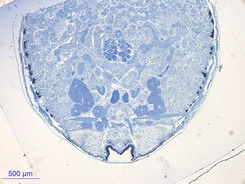

Supplement: S6 Fig — (ZIP) [file pone.0218486.s012.zip › T6055_469/T6085-0238_τ╝⌐σ░Åσñoσ░Å.jpg]

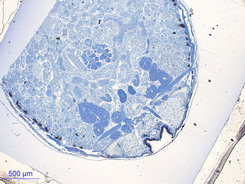

Supplement: S6 Fig — (ZIP) [file pone.0218486.s012.zip › T6055_469/T6085-0239_τ╝⌐σ░Åσñoσ░Å.jpg]

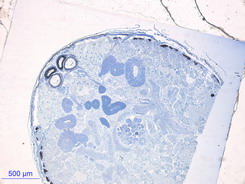

Supplement: S6 Fig — (ZIP) [file pone.0218486.s012.zip › T6055_469/T6085-0379_τ╝⌐σ░Åσñoσ░Å.jpg]

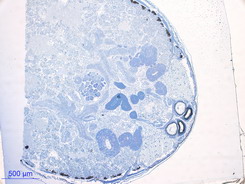

Supplement: S6 Fig — (ZIP) [file pone.0218486.s012.zip › T6055_469/T6085-0378_τ╝⌐σ░Åσñoσ░Å.jpg]
