# Supplementary figures and images for: Comparative morphology refines the conventional model of spider reproduction (part 2 of 5)
Source: PLoS One. 2019 Jul 5;14(7):e0218486. doi: 10.1371/journal.pone.0218486 (PMC6611574; doi:10.1371/journal.pone.0218486)

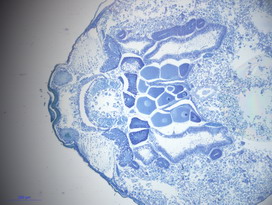

Supplement: S5 Fig — (ZIP) [file pone.0218486.s011.zip › T1570_237/T1570-0154_τ╝⌐σ░Åσñoσ░Å.jpg]

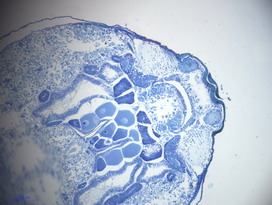

Supplement: S5 Fig — (ZIP) [file pone.0218486.s011.zip › T1570_237/T1570-0155_τ╝⌐σ░Åσñoσ░Å.jpg]

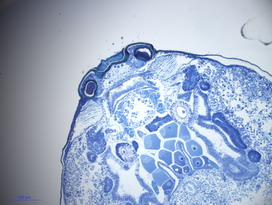

Supplement: S5 Fig — (ZIP) [file pone.0218486.s011.zip › T1570_237/T1570-0123_τ╝⌐σ░Åσñoσ░Å.jpg]

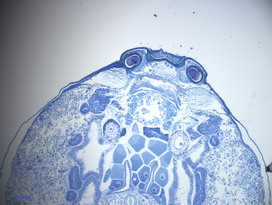

Supplement: S5 Fig — (ZIP) [file pone.0218486.s011.zip › T1570_237/T1570-0122_τ╝⌐σ░Åσñoσ░Å.jpg]

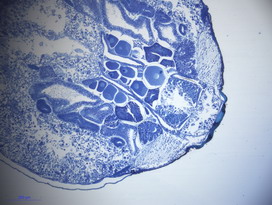

Supplement: S5 Fig — (ZIP) [file pone.0218486.s011.zip › T1570_237/T1570-0204_τ╝⌐σ░Åσñoσ░Å.jpg]

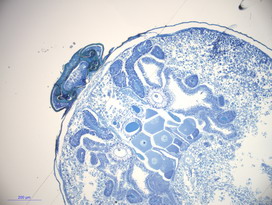

Supplement: S5 Fig — (ZIP) [file pone.0218486.s011.zip › T1570_237/T1570-0062_τ╝⌐σ░Åσñoσ░Å.jpg]

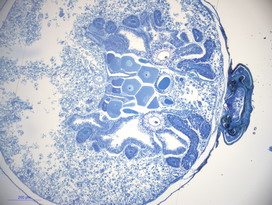

Supplement: S5 Fig — (ZIP) [file pone.0218486.s011.zip › T1570_237/T1570-0063_τ╝⌐σ░Åσñoσ░Å.jpg]

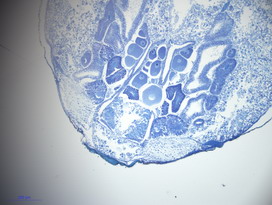

Supplement: S5 Fig — (ZIP) [file pone.0218486.s011.zip › T1570_237/T1570-0205_τ╝⌐σ░Åσñoσ░Å.jpg]

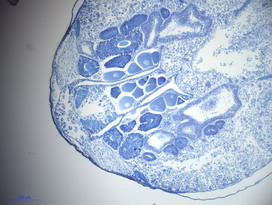

Supplement: S5 Fig — (ZIP) [file pone.0218486.s011.zip › T1570_237/T1570-0230_τ╝⌐σ░Åσñoσ░Å.jpg]

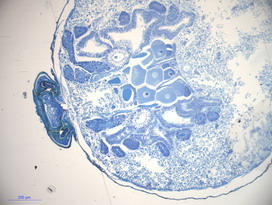

Supplement: S5 Fig — (ZIP) [file pone.0218486.s011.zip › T1570_237/T1570-0056_τ╝⌐σ░Åσñoσ░Å.jpg]

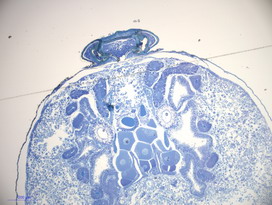

Supplement: S5 Fig — (ZIP) [file pone.0218486.s011.zip › T1570_237/T1570-0057_τ╝⌐σ░Åσñoσ░Å.jpg]

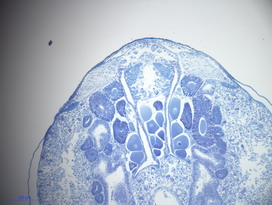

Supplement: S5 Fig — (ZIP) [file pone.0218486.s011.zip › T1570_237/T1570-0231_τ╝⌐σ░Åσñoσ░Å.jpg]

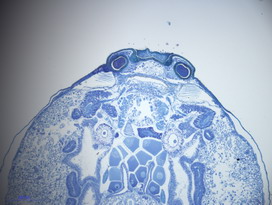

Supplement: S5 Fig — (ZIP) [file pone.0218486.s011.zip › T1570_237/T1570-0117_τ╝⌐σ░Åσñoσ░Å.jpg]

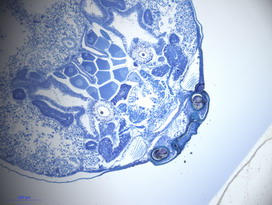

Supplement: S5 Fig — (ZIP) [file pone.0218486.s011.zip › T1570_237/T1570-0116_τ╝⌐σ░Åσñoσ░Å.jpg]

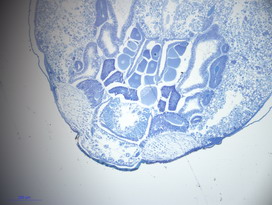

Supplement: S5 Fig — (ZIP) [file pone.0218486.s011.zip › T1570_237/T1570-0185_τ╝⌐σ░Åσñoσ░Å.jpg]

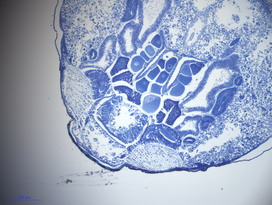

Supplement: S5 Fig — (ZIP) [file pone.0218486.s011.zip › T1570_237/T1570-0184_τ╝⌐σ░Åσñoσ░Å.jpg]

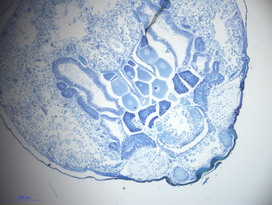

Supplement: S5 Fig — (ZIP) [file pone.0218486.s011.zip › T1570_237/T1570-0160_τ╝⌐σ░Åσñoσ░Å.jpg]

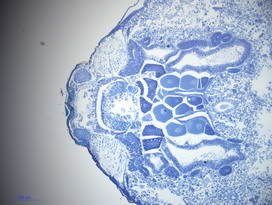

Supplement: S5 Fig — (ZIP) [file pone.0218486.s011.zip › T1570_237/T1570-0161_τ╝⌐σ░Åσñoσ░Å.jpg]

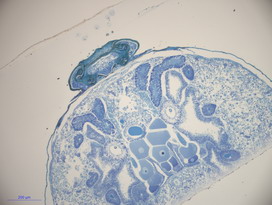

Supplement: S5 Fig — (ZIP) [file pone.0218486.s011.zip › T1570_237/T1570-0068_τ╝⌐σ░Åσñoσ░Å.jpg]

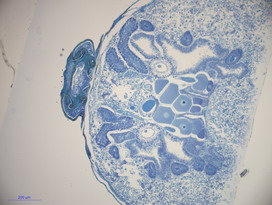

Supplement: S5 Fig — (ZIP) [file pone.0218486.s011.zip › T1570_237/T1570-0069_τ╝⌐σ░Åσñoσ░Å.jpg]

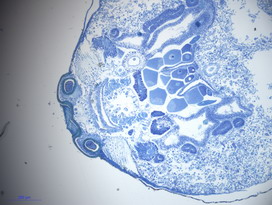

Supplement: S5 Fig — (ZIP) [file pone.0218486.s011.zip › T1570_237/T1570-0129_τ╝⌐σ░Åσñoσ░Å.jpg]

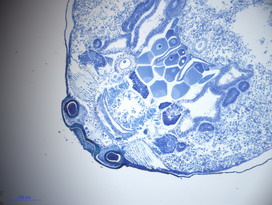

Supplement: S5 Fig — (ZIP) [file pone.0218486.s011.zip › T1570_237/T1570-0128_τ╝⌐σ░Åσñoσ░Å.jpg]

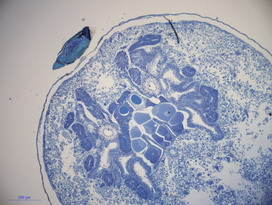

Supplement: S5 Fig — (ZIP) [file pone.0218486.s011.zip › T1570_237/T1570-0021_τ╝⌐σ░Åσñoσ░Å.jpg]

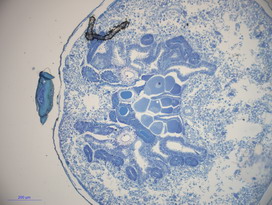

Supplement: S5 Fig — (ZIP) [file pone.0218486.s011.zip › T1570_237/T1570-0020_τ╝⌐σ░Åσñoσ░Å.jpg]

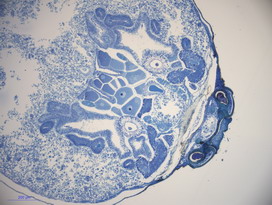

Supplement: S5 Fig — (ZIP) [file pone.0218486.s011.zip › T1570_237/T1570-0104_τ╝⌐σ░Åσñoσ░Å.jpg]

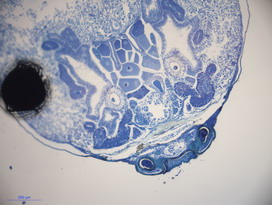

Supplement: S5 Fig — (ZIP) [file pone.0218486.s011.zip › T1570_237/T1570-0105_τ╝⌐σ░Åσñoσ░Å.jpg]

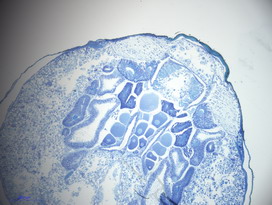

Supplement: S5 Fig — (ZIP) [file pone.0218486.s011.zip › T1570_237/T1570-0196_τ╝⌐σ░Åσñoσ░Å.jpg]

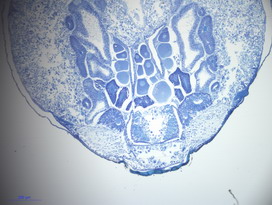

Supplement: S5 Fig — (ZIP) [file pone.0218486.s011.zip › T1570_237/T1570-0197_τ╝⌐σ░Åσñoσ░Å.jpg]

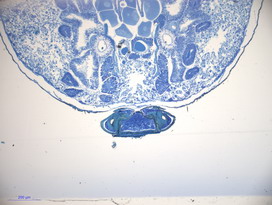

Supplement: S5 Fig — (ZIP) [file pone.0218486.s011.zip › T1570_237/T1570-0045_τ╝⌐σ░Åσñoσ░Å.jpg]

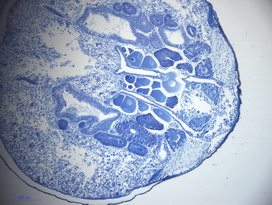

Supplement: S5 Fig — (ZIP) [file pone.0218486.s011.zip › T1570_237/T1570-0223_τ╝⌐σ░Åσñoσ░Å.jpg]

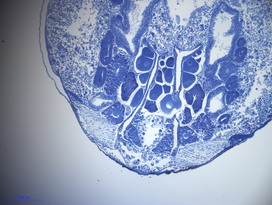

Supplement: S5 Fig — (ZIP) [file pone.0218486.s011.zip › T1570_237/T1570-0222_τ╝⌐σ░Åσñoσ░Å.jpg]

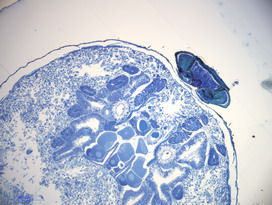

Supplement: S5 Fig — (ZIP) [file pone.0218486.s011.zip › T1570_237/T1570-0044_τ╝⌐σ░Åσñoσ░Å.jpg]

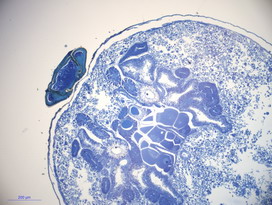

Supplement: S5 Fig — (ZIP) [file pone.0218486.s011.zip › T1570_237/T1570-0032_τ╝⌐σ░Åσñoσ░Å.jpg]

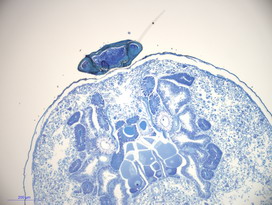

Supplement: S5 Fig — (ZIP) [file pone.0218486.s011.zip › T1570_237/T1570-0033_τ╝⌐σ░Åσñoσ░Å.jpg]

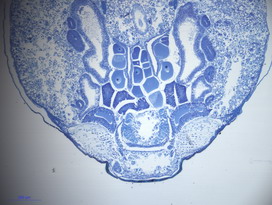

Supplement: S5 Fig — (ZIP) [file pone.0218486.s011.zip › T1570_237/T1570-0173_τ╝⌐σ░Åσñoσ░Å.jpg]

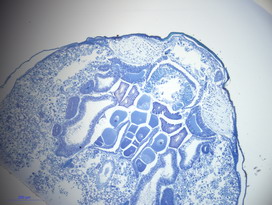

Supplement: S5 Fig — (ZIP) [file pone.0218486.s011.zip › T1570_237/T1570-0172_τ╝⌐σ░Åσñoσ░Å.jpg]

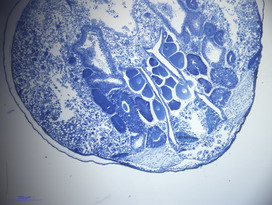

Supplement: S5 Fig — (ZIP) [file pone.0218486.s011.zip › T1570_237/T1570-0229_τ╝⌐σ░Åσñoσ░Å.jpg]

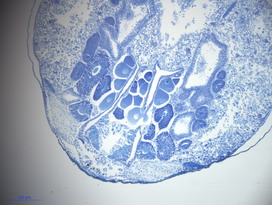

Supplement: S5 Fig — (ZIP) [file pone.0218486.s011.zip › T1570_237/T1570-0228_τ╝⌐σ░Åσñoσ░Å.jpg]

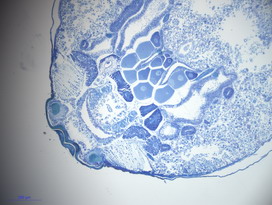

Supplement: S5 Fig — (ZIP) [file pone.0218486.s011.zip › T1570_237/T1570-0147_τ╝⌐σ░Åσñoσ░Å.jpg]

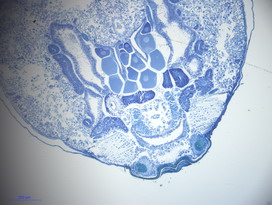

Supplement: S5 Fig — (ZIP) [file pone.0218486.s011.zip › T1570_237/T1570-0146_τ╝⌐σ░Åσñoσ░Å.jpg]

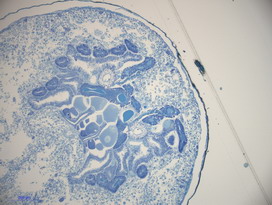

Supplement: S5 Fig — (ZIP) [file pone.0218486.s011.zip › T1570_237/T1570-0006_τ╝⌐σ░Åσñoσ░Å.jpg]

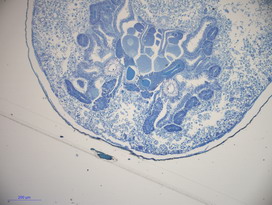

Supplement: S5 Fig — (ZIP) [file pone.0218486.s011.zip › T1570_237/T1570-0007_τ╝⌐σ░Åσñoσ░Å.jpg]

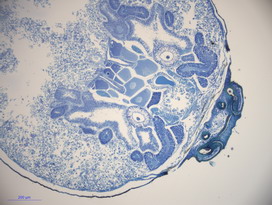

Supplement: S5 Fig — (ZIP) [file pone.0218486.s011.zip › T1570_237/T1570-0094_τ╝⌐σ░Åσñoσ░Å.jpg]

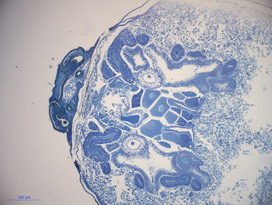

Supplement: S5 Fig — (ZIP) [file pone.0218486.s011.zip › T1570_237/T1570-0095_τ╝⌐σ░Åσñoσ░Å.jpg]

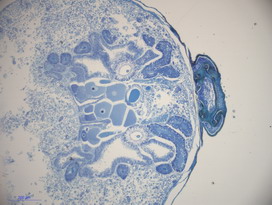

Supplement: S5 Fig — (ZIP) [file pone.0218486.s011.zip › T1570_237/T1570-0071_τ╝⌐σ░Åσñoσ░Å.jpg]

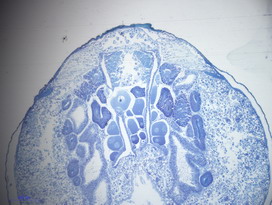

Supplement: S5 Fig — (ZIP) [file pone.0218486.s011.zip › T1570_237/T1570-0217_τ╝⌐σ░Åσñoσ░Å.jpg]

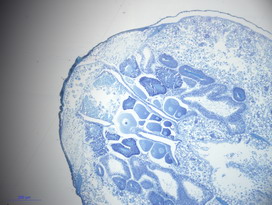

Supplement: S5 Fig — (ZIP) [file pone.0218486.s011.zip › T1570_237/T1570-0216_τ╝⌐σ░Åσñoσ░Å.jpg]

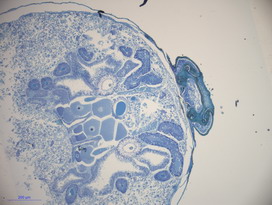

Supplement: S5 Fig — (ZIP) [file pone.0218486.s011.zip › T1570_237/T1570-0070_τ╝⌐σ░Åσñoσ░Å.jpg]

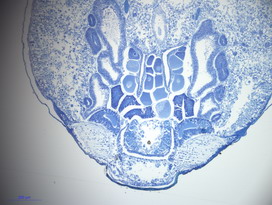

Supplement: S5 Fig — (ZIP) [file pone.0218486.s011.zip › T1570_237/T1570-0179_τ╝⌐σ░Åσñoσ░Å.jpg]

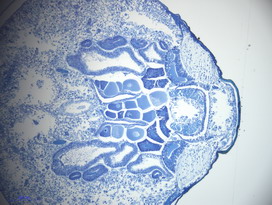

Supplement: S5 Fig — (ZIP) [file pone.0218486.s011.zip › T1570_237/T1570-0178_τ╝⌐σ░Åσñoσ░Å.jpg]

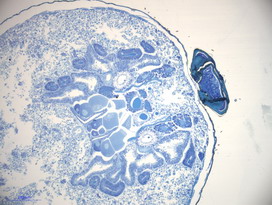

Supplement: S5 Fig — (ZIP) [file pone.0218486.s011.zip › T1570_237/T1570-0038_τ╝⌐σ░Åσñoσ░Å.jpg]

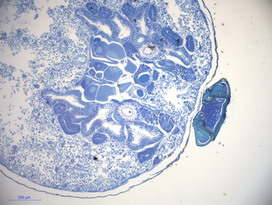

Supplement: S5 Fig — (ZIP) [file pone.0218486.s011.zip › T1570_237/T1570-0039_τ╝⌐σ░Åσñoσ░Å.jpg]

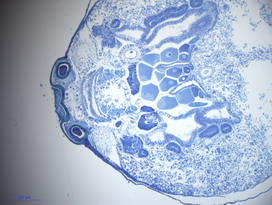

Supplement: S5 Fig — (ZIP) [file pone.0218486.s011.zip › T1570_237/T1570-0130_τ╝⌐σ░Åσñoσ░Å.jpg]

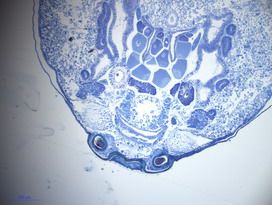

Supplement: S5 Fig — (ZIP) [file pone.0218486.s011.zip › T1570_237/T1570-0131_τ╝⌐σ░Åσñoσ░Å.jpg]

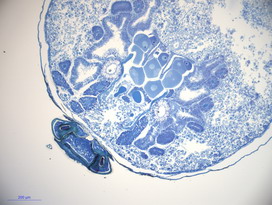

Supplement: S5 Fig — (ZIP) [file pone.0218486.s011.zip › T1570_237/T1570-0052_τ╝⌐σ░Åσñoσ░Å.jpg]

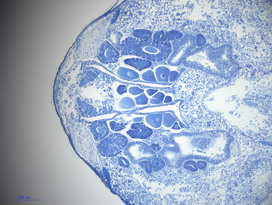

Supplement: S5 Fig — (ZIP) [file pone.0218486.s011.zip › T1570_237/T1570-0234_τ╝⌐σ░Åσñoσ░Å.jpg]

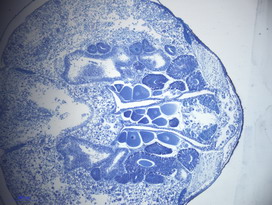

Supplement: S5 Fig — (ZIP) [file pone.0218486.s011.zip › T1570_237/T1570-0235_τ╝⌐σ░Åσñoσ░Å.jpg]

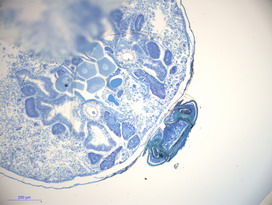

Supplement: S5 Fig — (ZIP) [file pone.0218486.s011.zip › T1570_237/T1570-0053_τ╝⌐σ░Åσñoσ░Å.jpg]

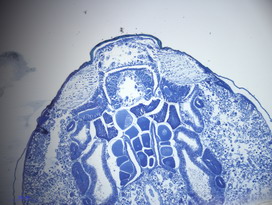

Supplement: S5 Fig — (ZIP) [file pone.0218486.s011.zip › T1570_237/T1570-0181_τ╝⌐σ░Åσñoσ░Å.jpg]

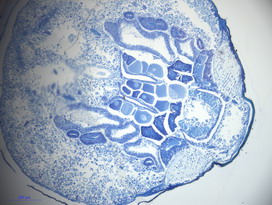

Supplement: S5 Fig — (ZIP) [file pone.0218486.s011.zip › T1570_237/T1570-0180_τ╝⌐σ░Åσñoσ░Å.jpg]

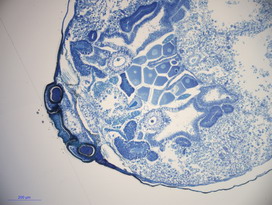

Supplement: S5 Fig — (ZIP) [file pone.0218486.s011.zip › T1570_237/T1570-0113_τ╝⌐σ░Åσñoσ░Å.jpg]

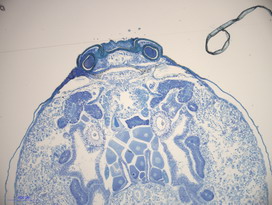

Supplement: S5 Fig — (ZIP) [file pone.0218486.s011.zip › T1570_237/T1570-0112_τ╝⌐σ░Åσñoσ░Å.jpg]

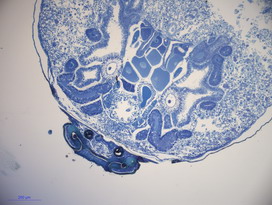

Supplement: S5 Fig — (ZIP) [file pone.0218486.s011.zip › T1570_237/T1570-0089_τ╝⌐σ░Åσñoσ░Å.jpg]

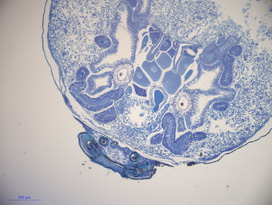

Supplement: S5 Fig — (ZIP) [file pone.0218486.s011.zip › T1570_237/T1570-0088_τ╝⌐σ░Åσñoσ░Å.jpg]

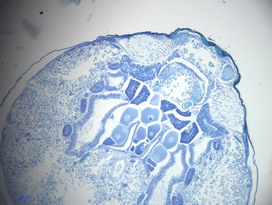

Supplement: S5 Fig — (ZIP) [file pone.0218486.s011.zip › T1570_237/T1570-0164_τ╝⌐σ░Åσñoσ░Å.jpg]

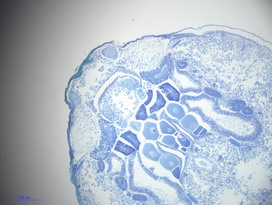

Supplement: S5 Fig — (ZIP) [file pone.0218486.s011.zip › T1570_237/T1570-0165_τ╝⌐σ░Åσñoσ░Å.jpg]

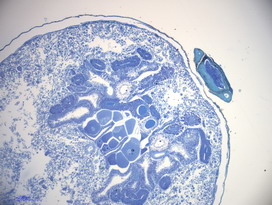

Supplement: S5 Fig — (ZIP) [file pone.0218486.s011.zip › T1570_237/T1570-0025_τ╝⌐σ░Åσñoσ░Å.jpg]

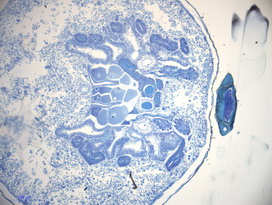

Supplement: S5 Fig — (ZIP) [file pone.0218486.s011.zip › T1570_237/T1570-0024_τ╝⌐σ░Åσñoσ░Å.jpg]

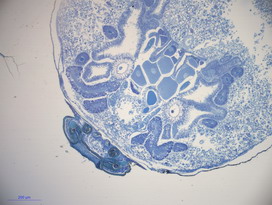

Supplement: S5 Fig — (ZIP) [file pone.0218486.s011.zip › T1570_237/T1570-0083_τ╝⌐σ░Åσñoσ░Å.jpg]

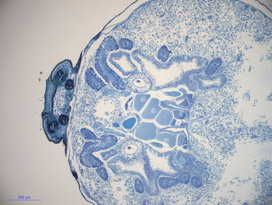

Supplement: S5 Fig — (ZIP) [file pone.0218486.s011.zip › T1570_237/T1570-0082_τ╝⌐σ░Åσñoσ░Å.jpg]

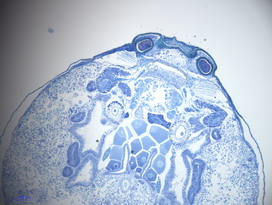

Supplement: S5 Fig — (ZIP) [file pone.0218486.s011.zip › T1570_237/T1570-0119_τ╝⌐σ░Åσñoσ░Å.jpg]

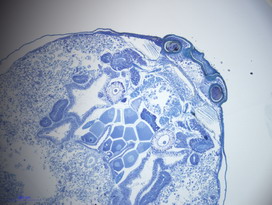

Supplement: S5 Fig — (ZIP) [file pone.0218486.s011.zip › T1570_237/T1570-0118_τ╝⌐σ░Åσñoσ░Å.jpg]

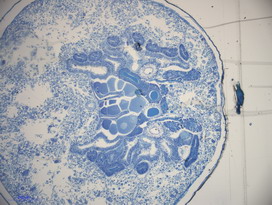

Supplement: S5 Fig — (ZIP) [file pone.0218486.s011.zip › T1570_237/T1570-0011_τ╝⌐σ░Åσñoσ░Å.jpg]

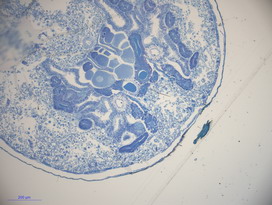

Supplement: S5 Fig — (ZIP) [file pone.0218486.s011.zip › T1570_237/T1570-0010_τ╝⌐σ░Åσñoσ░Å.jpg]

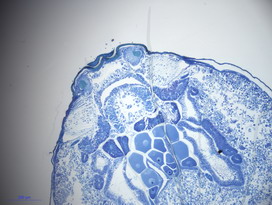

Supplement: S5 Fig — (ZIP) [file pone.0218486.s011.zip › T1570_237/T1570-0150_τ╝⌐σ░Åσñoσ░Å.jpg]

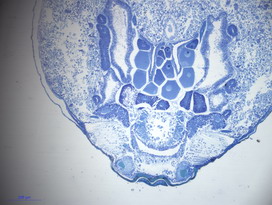

Supplement: S5 Fig — (ZIP) [file pone.0218486.s011.zip › T1570_237/T1570-0151_τ╝⌐σ░Åσñoσ░Å.jpg]

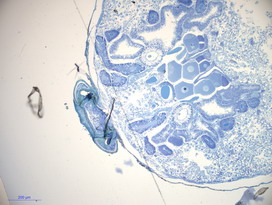

Supplement: S5 Fig — (ZIP) [file pone.0218486.s011.zip › T1570_237/T1570-0058_τ╝⌐σ░Åσñoσ░Å.jpg]

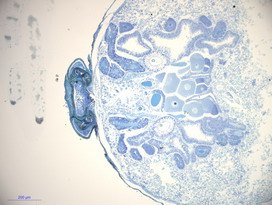

Supplement: S5 Fig — (ZIP) [file pone.0218486.s011.zip › T1570_237/T1570-0059_τ╝⌐σ░Åσñoσ░Å.jpg]

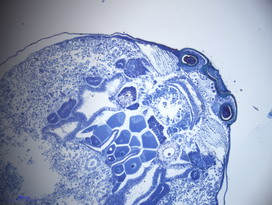

Supplement: S5 Fig — (ZIP) [file pone.0218486.s011.zip › T1570_237/T1570-0127_τ╝⌐σ░Åσñoσ░Å.jpg]

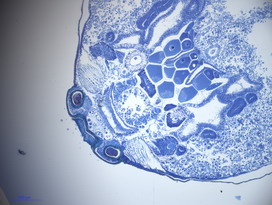

Supplement: S5 Fig — (ZIP) [file pone.0218486.s011.zip › T1570_237/T1570-0126_τ╝⌐σ░Åσñoσ░Å.jpg]

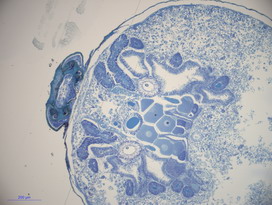

Supplement: S5 Fig — (ZIP) [file pone.0218486.s011.zip › T1570_237/T1570-0066_τ╝⌐σ░Åσñoσ░Å.jpg]

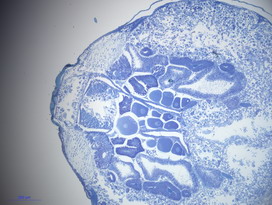

Supplement: S5 Fig — (ZIP) [file pone.0218486.s011.zip › T1570_237/T1570-0200_τ╝⌐σ░Åσñoσ░Å.jpg]

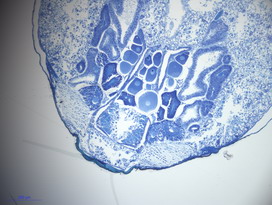

Supplement: S5 Fig — (ZIP) [file pone.0218486.s011.zip › T1570_237/T1570-0201_τ╝⌐σ░Åσñoσ░Å.jpg]

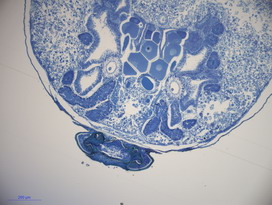

Supplement: S5 Fig — (ZIP) [file pone.0218486.s011.zip › T1570_237/T1570-0067_τ╝⌐σ░Åσñoσ░Å.jpg]

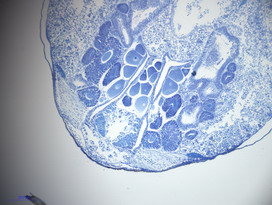

Supplement: S5 Fig — (ZIP) [file pone.0218486.s011.zip › T1570_237/T1570-0233_τ╝⌐σ░Åσñoσ░Å.jpg]

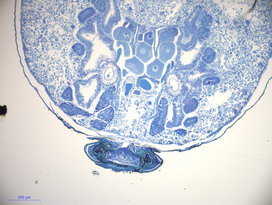

Supplement: S5 Fig — (ZIP) [file pone.0218486.s011.zip › T1570_237/T1570-0055_τ╝⌐σ░Åσñoσ░Å.jpg]

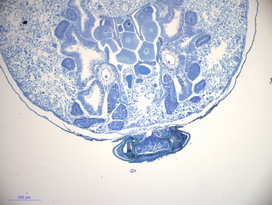

Supplement: S5 Fig — (ZIP) [file pone.0218486.s011.zip › T1570_237/T1570-0054_τ╝⌐σ░Åσñoσ░Å.jpg]

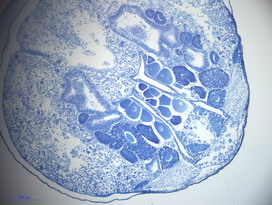

Supplement: S5 Fig — (ZIP) [file pone.0218486.s011.zip › T1570_237/T1570-0232_τ╝⌐σ░Åσñoσ░Å.jpg]

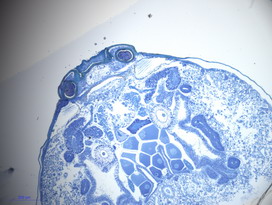

Supplement: S5 Fig — (ZIP) [file pone.0218486.s011.zip › T1570_237/T1570-0114_τ╝⌐σ░Åσñoσ░Å.jpg]

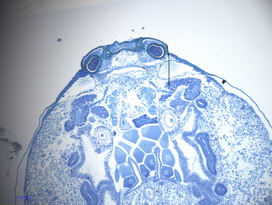

Supplement: S5 Fig — (ZIP) [file pone.0218486.s011.zip › T1570_237/T1570-0115_τ╝⌐σ░Åσñoσ░Å.jpg]

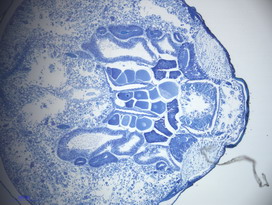

Supplement: S5 Fig — (ZIP) [file pone.0218486.s011.zip › T1570_237/T1570-0186_τ╝⌐σ░Åσñoσ░Å.jpg]

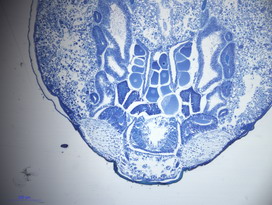

Supplement: S5 Fig — (ZIP) [file pone.0218486.s011.zip › T1570_237/T1570-0187_τ╝⌐σ░Åσñoσ░Å.jpg]

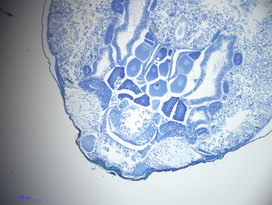

Supplement: S5 Fig — (ZIP) [file pone.0218486.s011.zip › T1570_237/T1570-0163_τ╝⌐σ░Åσñoσ░Å.jpg]

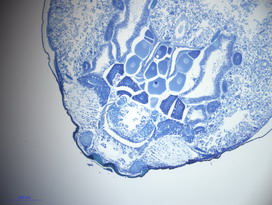

Supplement: S5 Fig — (ZIP) [file pone.0218486.s011.zip › T1570_237/T1570-0162_τ╝⌐σ░Åσñoσ░Å.jpg]

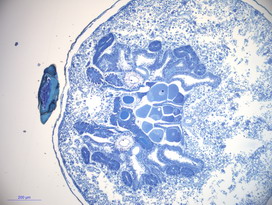

Supplement: S5 Fig — (ZIP) [file pone.0218486.s011.zip › T1570_237/T1570-0022_τ╝⌐σ░Åσñoσ░Å.jpg]

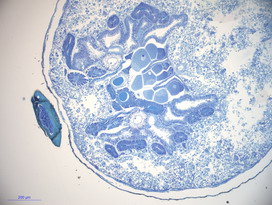

Supplement: S5 Fig — (ZIP) [file pone.0218486.s011.zip › T1570_237/T1570-0023_τ╝⌐σ░Åσñoσ░Å.jpg]

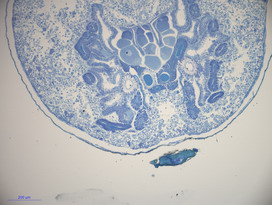

Supplement: S5 Fig — (ZIP) [file pone.0218486.s011.zip › T1570_237/T1570-0016_τ╝⌐σ░Åσñoσ░Å.jpg]

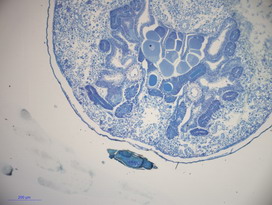

Supplement: S5 Fig — (ZIP) [file pone.0218486.s011.zip › T1570_237/T1570-0017_τ╝⌐σ░Åσñoσ░Å.jpg]

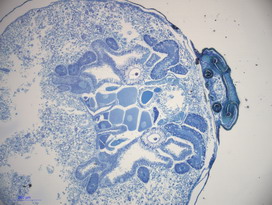

Supplement: S5 Fig — (ZIP) [file pone.0218486.s011.zip › T1570_237/T1570-0084_τ╝⌐σ░Åσñoσ░Å.jpg]

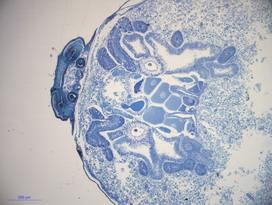

Supplement: S5 Fig — (ZIP) [file pone.0218486.s011.zip › T1570_237/T1570-0085_τ╝⌐σ░Åσñoσ░Å.jpg]
