# Supplementary figures and images for: Comparative morphology refines the conventional model of spider reproduction (part 5 of 5)
Source: PLoS One. 2019 Jul 5;14(7):e0218486. doi: 10.1371/journal.pone.0218486 (PMC6611574; doi:10.1371/journal.pone.0218486)

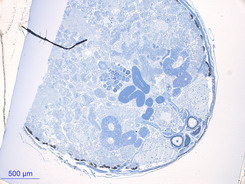

Supplement: S6 Fig — (ZIP) [file pone.0218486.s012.zip › T6055_469/T6085-0427_τ╝⌐σ░Åσñoσ░Å.jpg]

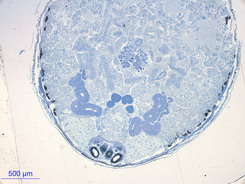

Supplement: S6 Fig — (ZIP) [file pone.0218486.s012.zip › T6055_469/T6085-0314_τ╝⌐σ░Åσñoσ░Å.jpg]

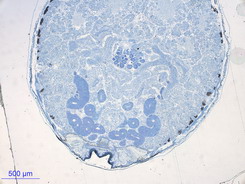

Supplement: S6 Fig — (ZIP) [file pone.0218486.s012.zip › T6055_469/T6085-0172_τ╝⌐σ░Åσñoσ░Å.jpg]

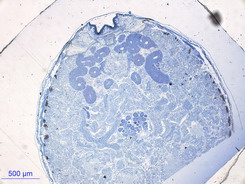

Supplement: S6 Fig — (ZIP) [file pone.0218486.s012.zip › T6055_469/T6085-0173_τ╝⌐σ░Åσñoσ░Å.jpg]
