# Supplementary figures and images for: Comparative morphology refines the conventional model of spider reproduction (part 4 of 5)
Source: PLoS One. 2019 Jul 5;14(7):e0218486. doi: 10.1371/journal.pone.0218486 (PMC6611574; doi:10.1371/journal.pone.0218486)

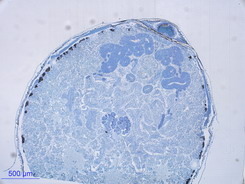

Supplement: S6 Fig — (ZIP) [file pone.0218486.s012.zip › T6055_469/T6085-0085_τ╝⌐σ░Åσñoσ░Å.jpg]

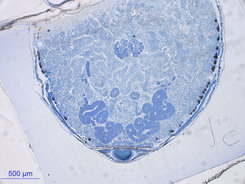

Supplement: S6 Fig — (ZIP) [file pone.0218486.s012.zip › T6055_469/T6085-0084_τ╝⌐σ░Åσñoσ░Å.jpg]

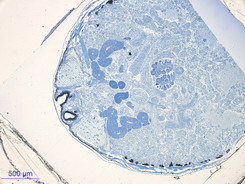

Supplement: S6 Fig — (ZIP) [file pone.0218486.s012.zip › T6055_469/T6085-0271_τ╝⌐σ░Åσñoσ░Å.jpg]

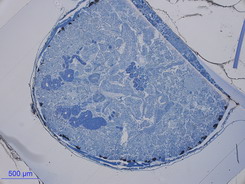

Supplement: S6 Fig — (ZIP) [file pone.0218486.s012.zip › T6055_469/T6085-0017_τ╝⌐σ░Åσñoσ░Å.jpg]

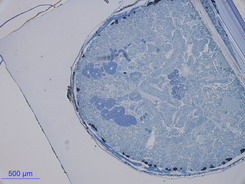

Supplement: S6 Fig — (ZIP) [file pone.0218486.s012.zip › T6055_469/T6085-0016_τ╝⌐σ░Åσñoσ░Å.jpg]

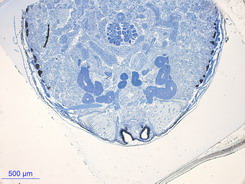

Supplement: S6 Fig — (ZIP) [file pone.0218486.s012.zip › T6055_469/T6085-0270_τ╝⌐σ░Åσñoσ░Å.jpg]

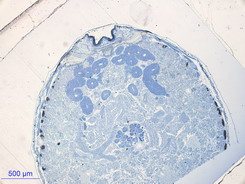

Supplement: S6 Fig — (ZIP) [file pone.0218486.s012.zip › T6055_469/T6085-0168_τ╝⌐σ░Åσñoσ░Å.jpg]

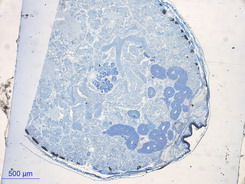

Supplement: S6 Fig — (ZIP) [file pone.0218486.s012.zip › T6055_469/T6085-0169_τ╝⌐σ░Åσñoσ░Å.jpg]

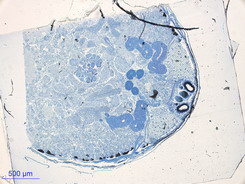

Supplement: S6 Fig — (ZIP) [file pone.0218486.s012.zip › T6055_469/T6085-0294_τ╝⌐σ░Åσñoσ░Å.jpg]

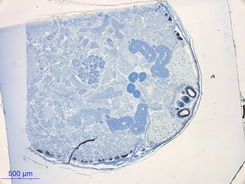

Supplement: S6 Fig — (ZIP) [file pone.0218486.s012.zip › T6055_469/T6085-0295_τ╝⌐σ░Åσñoσ░Å.jpg]

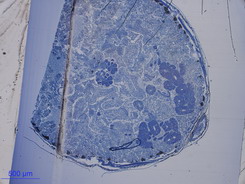

Supplement: S6 Fig — (ZIP) [file pone.0218486.s012.zip › T6055_469/T6085-0060_τ╝⌐σ░Åσñoσ░Å.jpg]

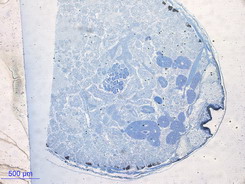

Supplement: S6 Fig — (ZIP) [file pone.0218486.s012.zip › T6055_469/T6085-0206_τ╝⌐σ░Åσñoσ░Å.jpg]

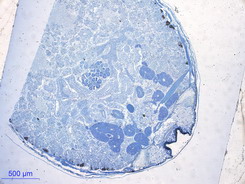

Supplement: S6 Fig — (ZIP) [file pone.0218486.s012.zip › T6055_469/T6085-0207_τ╝⌐σ░Åσñoσ░Å.jpg]

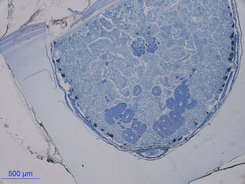

Supplement: S6 Fig — (ZIP) [file pone.0218486.s012.zip › T6055_469/T6085-0061_τ╝⌐σ░Åσñoσ░Å.jpg]

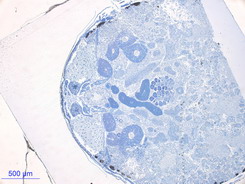

Supplement: S6 Fig — (ZIP) [file pone.0218486.s012.zip › T6055_469/T6085-0475_τ╝⌐σ░Åσñoσ░Å.jpg]

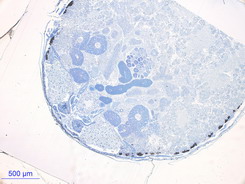

Supplement: S6 Fig — (ZIP) [file pone.0218486.s012.zip › T6055_469/T6085-0474_τ╝⌐σ░Åσñoσ░Å.jpg]

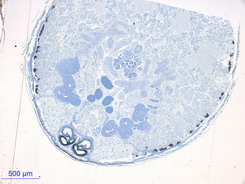

Supplement: S6 Fig — (ZIP) [file pone.0218486.s012.zip › T6055_469/T6085-0347_τ╝⌐σ░Åσñoσ░Å.jpg]

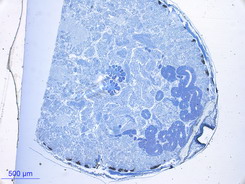

Supplement: S6 Fig — (ZIP) [file pone.0218486.s012.zip › T6055_469/T6085-0121_τ╝⌐σ░Åσñoσ░Å.jpg]

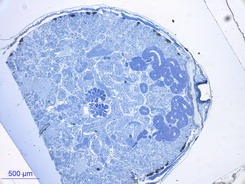

Supplement: S6 Fig — (ZIP) [file pone.0218486.s012.zip › T6055_469/T6085-0120_τ╝⌐σ░Åσñoσ░Å.jpg]

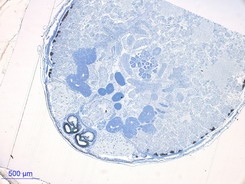

Supplement: S6 Fig — (ZIP) [file pone.0218486.s012.zip › T6055_469/T6085-0346_τ╝⌐σ░Åσñoσ░Å.jpg]

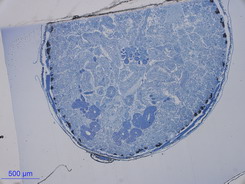

Supplement: S6 Fig — (ZIP) [file pone.0218486.s012.zip › T6055_469/T6085-0029_τ╝⌐σ░Åσñoσ░Å.jpg]

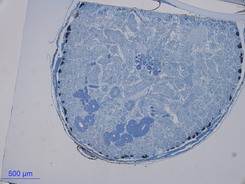

Supplement: S6 Fig — (ZIP) [file pone.0218486.s012.zip › T6055_469/T6085-0028_τ╝⌐σ░Åσñoσ░Å.jpg]

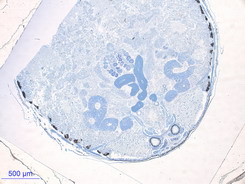

Supplement: S6 Fig — (ZIP) [file pone.0218486.s012.zip › T6055_469/T6085-0446_τ╝⌐σ░Åσñoσ░Å.jpg]

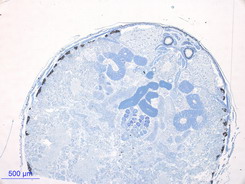

Supplement: S6 Fig — (ZIP) [file pone.0218486.s012.zip › T6055_469/T6085-0447_τ╝⌐σ░Åσñoσ░Å.jpg]

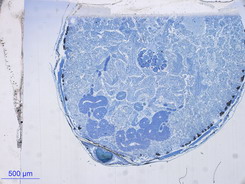

Supplement: S6 Fig — (ZIP) [file pone.0218486.s012.zip › T6055_469/T6085-0088_τ╝⌐σ░Åσñoσ░Å.jpg]

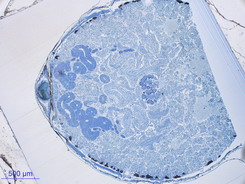

Supplement: S6 Fig — (ZIP) [file pone.0218486.s012.zip › T6055_469/T6085-0089_τ╝⌐σ░Åσñoσ░Å.jpg]

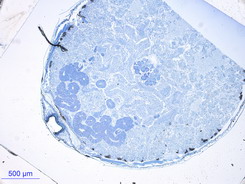

Supplement: S6 Fig — (ZIP) [file pone.0218486.s012.zip › T6055_469/T6085-0112_τ╝⌐σ░Åσñoσ░Å.jpg]

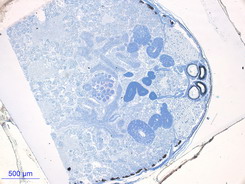

Supplement: S6 Fig — (ZIP) [file pone.0218486.s012.zip › T6055_469/T6085-0374_τ╝⌐σ░Åσñoσ░Å.jpg]

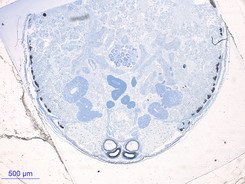

Supplement: S6 Fig — (ZIP) [file pone.0218486.s012.zip › T6055_469/T6085-0375_τ╝⌐σ░Åσñoσ░Å.jpg]

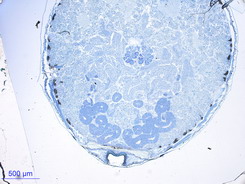

Supplement: S6 Fig — (ZIP) [file pone.0218486.s012.zip › T6055_469/T6085-0113_τ╝⌐σ░Åσñoσ░Å.jpg]

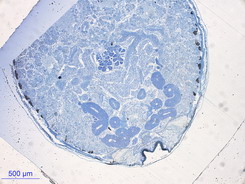

Supplement: S6 Fig — (ZIP) [file pone.0218486.s012.zip › T6055_469/T6085-0180_τ╝⌐σ░Åσñoσ░Å.jpg]

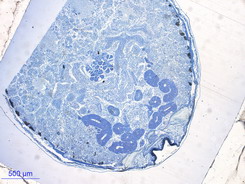

Supplement: S6 Fig — (ZIP) [file pone.0218486.s012.zip › T6055_469/T6085-0181_τ╝⌐σ░Åσñoσ░Å.jpg]

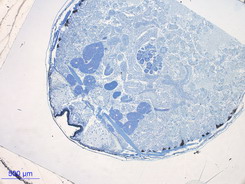

Supplement: S6 Fig — (ZIP) [file pone.0218486.s012.zip › T6055_469/T6085-0235_τ╝⌐σ░Åσñoσ░Å.jpg]

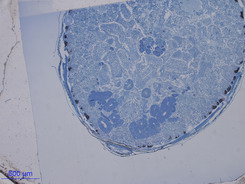

Supplement: S6 Fig — (ZIP) [file pone.0218486.s012.zip › T6055_469/T6085-0053_τ╝⌐σ░Åσñoσ░Å.jpg]

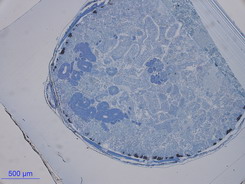

Supplement: S6 Fig — (ZIP) [file pone.0218486.s012.zip › T6055_469/T6085-0052_τ╝⌐σ░Åσñoσ░Å.jpg]

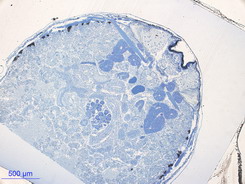

Supplement: S6 Fig — (ZIP) [file pone.0218486.s012.zip › T6055_469/T6085-0234_τ╝⌐σ░Åσñoσ░Å.jpg]

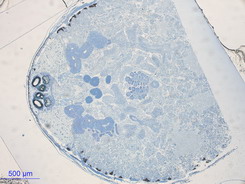

Supplement: S6 Fig — (ZIP) [file pone.0218486.s012.zip › T6055_469/T6085-329_τ╝⌐σ░Åσñoσ░Å.jpg]

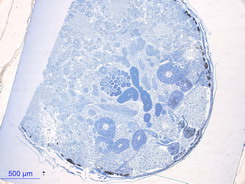

Supplement: S6 Fig — (ZIP) [file pone.0218486.s012.zip › T6055_469/T6085-0478_τ╝⌐σ░Åσñoσ░Å.jpg]

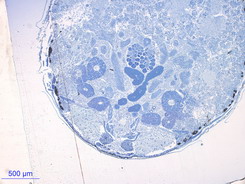

Supplement: S6 Fig — (ZIP) [file pone.0218486.s012.zip › T6055_469/T6085-0479_τ╝⌐σ░Åσñoσ░Å.jpg]

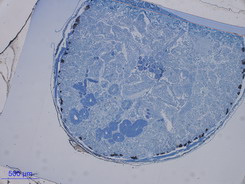

Supplement: S6 Fig — (ZIP) [file pone.0218486.s012.zip › T6055_469/T6085-0024_τ╝⌐σ░Åσñoσ░Å.jpg]

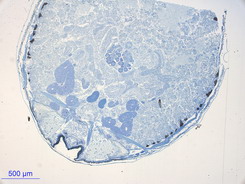

Supplement: S6 Fig — (ZIP) [file pone.0218486.s012.zip › T6055_469/T6085-0242_τ╝⌐σ░Åσñoσ░Å.jpg]

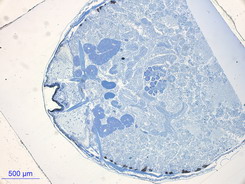

Supplement: S6 Fig — (ZIP) [file pone.0218486.s012.zip › T6055_469/T6085-0243_τ╝⌐σ░Åσñoσ░Å.jpg]

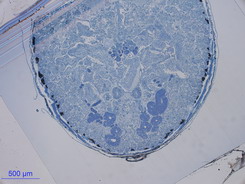

Supplement: S6 Fig — (ZIP) [file pone.0218486.s012.zip › T6055_469/T6085-0025_τ╝⌐σ░Åσñoσ░Å.jpg]

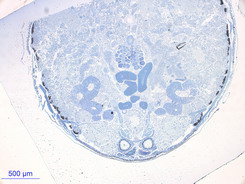

Supplement: S6 Fig — (ZIP) [file pone.0218486.s012.zip › T6055_469/T6085-0431_τ╝⌐σ░Åσñoσ░Å.jpg]

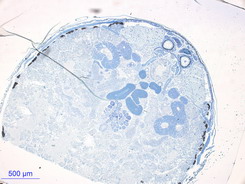

Supplement: S6 Fig — (ZIP) [file pone.0218486.s012.zip › T6055_469/T6085-0430_τ╝⌐σ░Åσñoσ░Å.jpg]

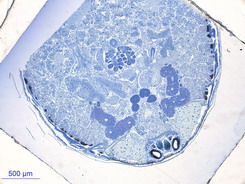

Supplement: S6 Fig — (ZIP) [file pone.0218486.s012.zip › T6055_469/T6085-0299_τ╝⌐σ░Åσñoσ░Å.jpg]

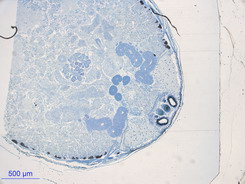

Supplement: S6 Fig — (ZIP) [file pone.0218486.s012.zip › T6055_469/T6085-0298_τ╝⌐σ░Åσñoσ░Å.jpg]

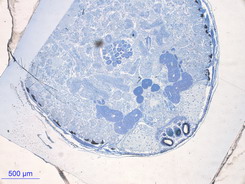

Supplement: S6 Fig — (ZIP) [file pone.0218486.s012.zip › T6055_469/T6085-0303_τ╝⌐σ░Åσñoσ░Å.jpg]

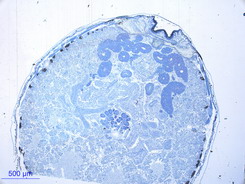

Supplement: S6 Fig — (ZIP) [file pone.0218486.s012.zip › T6055_469/T6085-0165_τ╝⌐σ░Åσñoσ░Å.jpg]

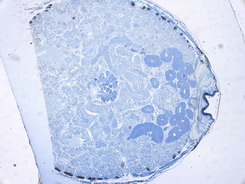

Supplement: S6 Fig — (ZIP) [file pone.0218486.s012.zip › T6055_469/T6085-0164_τ╝⌐σ░Åσñoσ░Å.jpg]

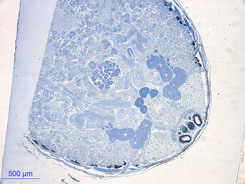

Supplement: S6 Fig — (ZIP) [file pone.0218486.s012.zip › T6055_469/T6085-0302_τ╝⌐σ░Åσñoσ░Å.jpg]

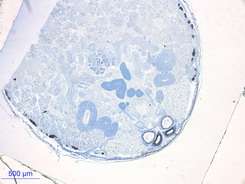

Supplement: S6 Fig — (ZIP) [file pone.0218486.s012.zip › T6055_469/T6085-0391_τ╝⌐σ░Åσñoσ░Å.jpg]

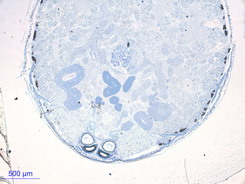

Supplement: S6 Fig — (ZIP) [file pone.0218486.s012.zip › T6055_469/T6085-0390_τ╝⌐σ░Åσñoσ░Å.jpg]

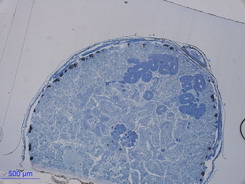

Supplement: S6 Fig — (ZIP) [file pone.0218486.s012.zip › T6055_469/T6085-0059_τ╝⌐σ░Åσñoσ░Å.jpg]

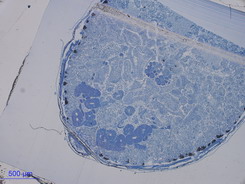

Supplement: S6 Fig — (ZIP) [file pone.0218486.s012.zip › T6055_469/T6085-0058_τ╝⌐σ░Åσñoσ░Å.jpg]

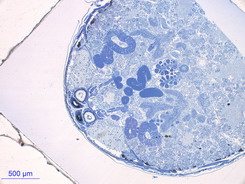

Supplement: S6 Fig — (ZIP) [file pone.0218486.s012.zip › T6055_469/T6085-0405_τ╝⌐σ░Åσñoσ░Å.jpg]

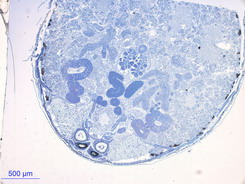

Supplement: S6 Fig — (ZIP) [file pone.0218486.s012.zip › T6055_469/T6085-0404_τ╝⌐σ░Åσñoσ░Å.jpg]

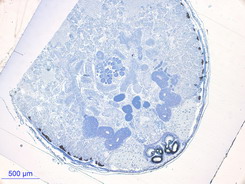

Supplement: S6 Fig — (ZIP) [file pone.0218486.s012.zip › T6055_469/T6085-0337_τ╝⌐σ░Åσñoσ░Å.jpg]

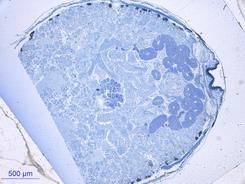

Supplement: S6 Fig — (ZIP) [file pone.0218486.s012.zip › T6055_469/T6085-0151_τ╝⌐σ░Åσñoσ░Å.jpg]

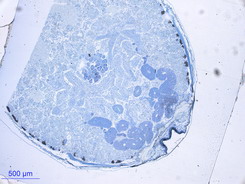

Supplement: S6 Fig — (ZIP) [file pone.0218486.s012.zip › T6055_469/T6085-0150_τ╝⌐σ░Åσñoσ░Å.jpg]

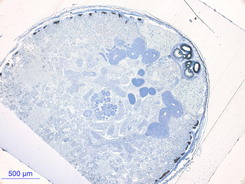

Supplement: S6 Fig — (ZIP) [file pone.0218486.s012.zip › T6055_469/T6085-0336_τ╝⌐σ░Åσñoσ░Å.jpg]

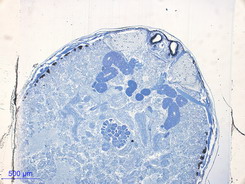

Supplement: S6 Fig — (ZIP) [file pone.0218486.s012.zip › T6055_469/T6085-0276_τ╝⌐σ░Åσñoσ░Å.jpg]

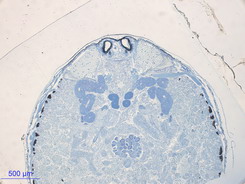

Supplement: S6 Fig — (ZIP) [file pone.0218486.s012.zip › T6055_469/T6085-0277_τ╝⌐σ░Åσñoσ░Å.jpg]

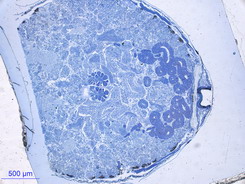

Supplement: S6 Fig — (ZIP) [file pone.0218486.s012.zip › T6055_469/T6085-0118_τ╝⌐σ░Åσñoσ░Å.jpg]

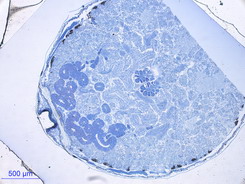

Supplement: S6 Fig — (ZIP) [file pone.0218486.s012.zip › T6055_469/T6085-0119_τ╝⌐σ░Åσñoσ░Å.jpg]

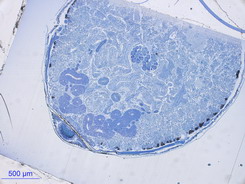

Supplement: S6 Fig — (ZIP) [file pone.0218486.s012.zip › T6055_469/T6085-0082_τ╝⌐σ░Åσñoσ░Å.jpg]

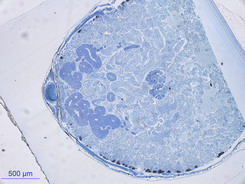

Supplement: S6 Fig — (ZIP) [file pone.0218486.s012.zip › T6055_469/T6085-0083_τ╝⌐σ░Åσñoσ░Å.jpg]

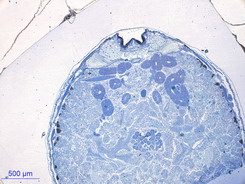

Supplement: S6 Fig — (ZIP) [file pone.0218486.s012.zip › T6055_469/T6085-0201_τ╝⌐σ░Åσñoσ░Å.jpg]

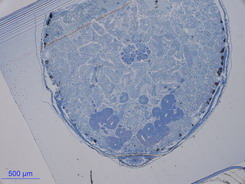

Supplement: S6 Fig — (ZIP) [file pone.0218486.s012.zip › T6055_469/T6085-0067_τ╝⌐σ░Åσñoσ░Å.jpg]

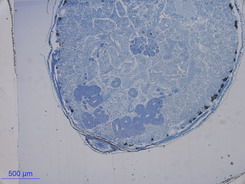

Supplement: S6 Fig — (ZIP) [file pone.0218486.s012.zip › T6055_469/T6085-0066_τ╝⌐σ░Åσñoσ░Å.jpg]

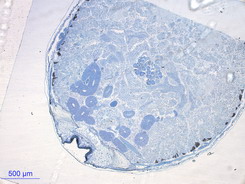

Supplement: S6 Fig — (ZIP) [file pone.0218486.s012.zip › T6055_469/T6085-0200_τ╝⌐σ░Åσñoσ░Å.jpg]

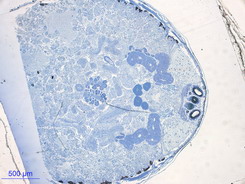

Supplement: S6 Fig — (ZIP) [file pone.0218486.s012.zip › T6055_469/T6085-0309_τ╝⌐σ░Åσñoσ░Å.jpg]

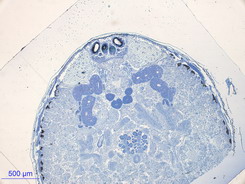

Supplement: S6 Fig — (ZIP) [file pone.0218486.s012.zip › T6055_469/T6085-0308_τ╝⌐σ░Åσñoσ░Å.jpg]

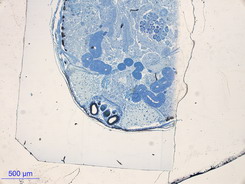

Supplement: S6 Fig — (ZIP) [file pone.0218486.s012.zip › T6055_469/T6085-0293_τ╝⌐σ░Åσñoσ░Å.jpg]

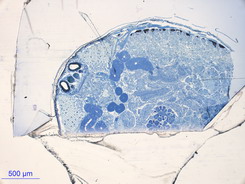

Supplement: S6 Fig — (ZIP) [file pone.0218486.s012.zip › T6055_469/T6085-0292_τ╝⌐σ░Åσñoσ░Å.jpg]

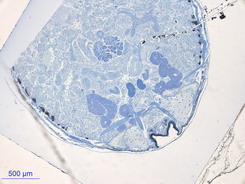

Supplement: S6 Fig — (ZIP) [file pone.0218486.s012.zip › T6055_469/T6085-0248_τ╝⌐σ░Åσñoσ░Å.jpg]

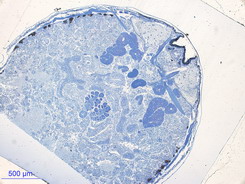

Supplement: S6 Fig — (ZIP) [file pone.0218486.s012.zip › T6055_469/T6085-0249_τ╝⌐σ░Åσñoσ░Å.jpg]

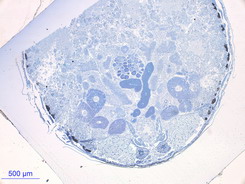

Supplement: S6 Fig — (ZIP) [file pone.0218486.s012.zip › T6055_469/T6085-0472_τ╝⌐σ░Åσñoσ░Å.jpg]

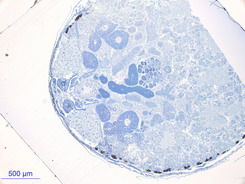

Supplement: S6 Fig — (ZIP) [file pone.0218486.s012.zip › T6055_469/T6085-0473_τ╝⌐σ░Åσñoσ░Å.jpg]

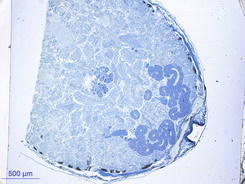

Supplement: S6 Fig — (ZIP) [file pone.0218486.s012.zip › T6055_469/T6085-0126_τ╝⌐σ░Åσñoσ░Å.jpg]

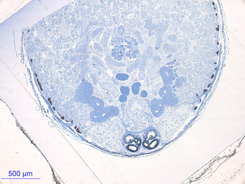

Supplement: S6 Fig — (ZIP) [file pone.0218486.s012.zip › T6055_469/T6085-0340_τ╝⌐σ░Åσñoσ░Å.jpg]

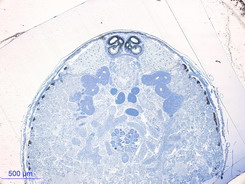

Supplement: S6 Fig — (ZIP) [file pone.0218486.s012.zip › T6055_469/T6085-0341_τ╝⌐σ░Åσñoσ░Å.jpg]

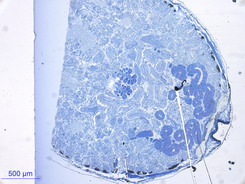

Supplement: S6 Fig — (ZIP) [file pone.0218486.s012.zip › T6055_469/T6085-0127_τ╝⌐σ░Åσñoσ░Å.jpg]

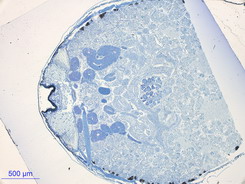

Supplement: S6 Fig — (ZIP) [file pone.0218486.s012.zip › T6055_469/T6085-0222_τ╝⌐σ░Åσñoσ░Å.jpg]

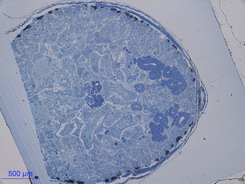

Supplement: S6 Fig — (ZIP) [file pone.0218486.s012.zip › T6055_469/T6085-0044_τ╝⌐σ░Åσñoσ░Å.jpg]

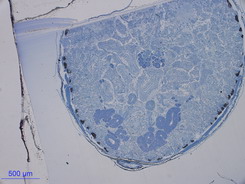

Supplement: S6 Fig — (ZIP) [file pone.0218486.s012.zip › T6055_469/T6085-0045_τ╝⌐σ░Åσñoσ░Å.jpg]

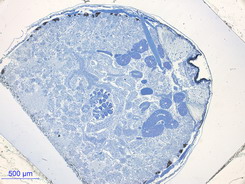

Supplement: S6 Fig — (ZIP) [file pone.0218486.s012.zip › T6055_469/T6085-0223_τ╝⌐σ░Åσñoσ░Å.jpg]

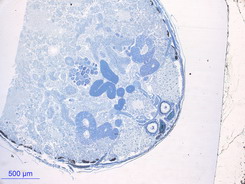

Supplement: S6 Fig — (ZIP) [file pone.0218486.s012.zip › T6055_469/T6085-0418_τ╝⌐σ░Åσñoσ░Å.jpg]

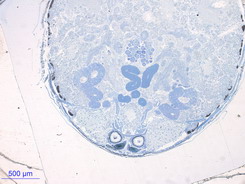

Supplement: S6 Fig — (ZIP) [file pone.0218486.s012.zip › T6055_469/T6085-0419_τ╝⌐σ░Åσñoσ░Å.jpg]

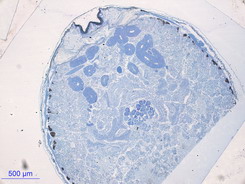

Supplement: S6 Fig — (ZIP) [file pone.0218486.s012.zip › T6055_469/T6085-0197_τ╝⌐σ░Åσñoσ░Å.jpg]

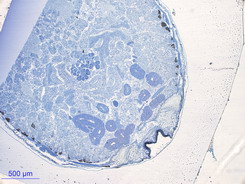

Supplement: S6 Fig — (ZIP) [file pone.0218486.s012.zip › T6055_469/T6085-0196_τ╝⌐σ░Åσñoσ░Å.jpg]

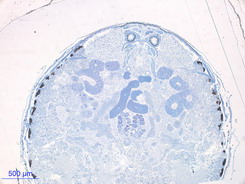

Supplement: S6 Fig — (ZIP) [file pone.0218486.s012.zip › T6055_469/T6085-0451_τ╝⌐σ░Åσñoσ░Å.jpg]

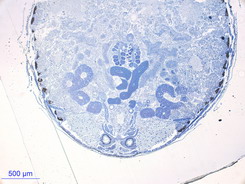

Supplement: S6 Fig — (ZIP) [file pone.0218486.s012.zip › T6055_469/T6085-0450_τ╝⌐σ░Åσñoσ░Å.jpg]

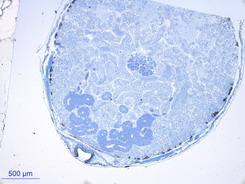

Supplement: S6 Fig — (ZIP) [file pone.0218486.s012.zip › T6055_469/T6085-0105_τ╝⌐σ░Åσñoσ░Å.jpg]

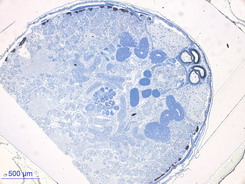

Supplement: S6 Fig — (ZIP) [file pone.0218486.s012.zip › T6055_469/T6085-0363_τ╝⌐σ░Åσñoσ░Å.jpg]

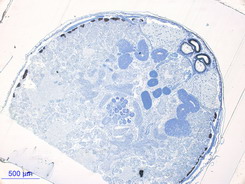

Supplement: S6 Fig — (ZIP) [file pone.0218486.s012.zip › T6055_469/T6085-0362_τ╝⌐σ░Åσñoσ░Å.jpg]

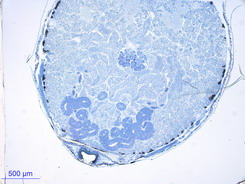

Supplement: S6 Fig — (ZIP) [file pone.0218486.s012.zip › T6055_469/T6085-0104_τ╝⌐σ░Åσñoσ░Å.jpg]

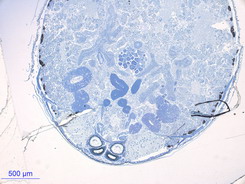

Supplement: S6 Fig — (ZIP) [file pone.0218486.s012.zip › T6055_469/T6085-0386_τ╝⌐σ░Åσñoσ░Å.jpg]

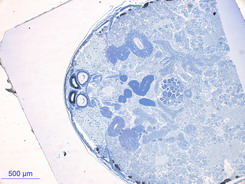

Supplement: S6 Fig — (ZIP) [file pone.0218486.s012.zip › T6055_469/T6085-0387_τ╝⌐σ░Åσñoσ░Å.jpg]

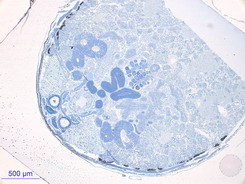

Supplement: S6 Fig — (ZIP) [file pone.0218486.s012.zip › T6055_469/T6085-0426_τ╝⌐σ░Åσñoσ░Å.jpg]
